# Human variability in isoform-specific UDP-glucuronosyltransferases: markers of acute and chronic exposure, polymorphisms and uncertainty factors

**DOI:** 10.1007/s00204-020-02765-8

**Published:** 2020-05-15

**Authors:** E. E. J. Kasteel, K. Darney, N. I. Kramer, J. L. C. M. Dorne, L. S. Lautz

**Affiliations:** 1 Institute for Risk Assessment Sciences (IRAS), Faculty of Veterinary Medicine, Utrecht University, P.O. Box 80.177, 3508 TD Utrecht, The Netherlands; 2 Risk Assessment Department, French Agency for Food, Environmental and Occupational Health and Safety (ANSES), 14 rue Pierre et Marie Curie, 94701 Maisons-Alfort, France; 3 European Food Safety Authority, Scientific Committee and Emerging Risks Unit, Via Carlo Magno 1A, 43126 Parma, Italy

**Keywords:** Human variability, Pharmacokinetics, Uncertainty factor, UGT, Polymorphism

## Abstract

UDP-glucuronosyltransferases (UGTs) are involved in phase II conjugation reactions of xenobiotics and differences in their isoform activities result in interindividual kinetic differences of UGT probe substrates. Here, extensive literature searches were performed to identify probe substrates (14) for various UGT isoforms (UGT1A1, UGT1A3, UGT1A4, UGT1A6, UGT1A9, UGT2B7 and UGT2B15) and frequencies of human polymorphisms. Chemical-specific pharmacokinetic data were collected in a database to quantify interindividual differences in markers of acute (Cmax) and chronic (area under the curve, clearance) exposure. Using this database, UGT-related uncertainty factors were derived and compared to the default factor (i.e. 3.16) allowing for interindividual differences in kinetics. Overall, results show that pharmacokinetic data are predominantly available for Caucasian populations and scarce for other populations of different geographical ancestry. Furthermore, the relationships between UGT polymorphisms and pharmacokinetic parameters are rarely addressed in the included studies. The data show that UGT-related uncertainty factors were mostly below the default toxicokinetic uncertainty factor of 3.16, with the exception of five probe substrates (1-OH-midazolam, ezetimibe, raltegravir, SN38 and trifluoperazine), with three of these substrates being metabolised by the polymorphic isoform 1A1. Data gaps and future work to integrate UGT-related variability distributions with in vitro data to develop quantitative in vitro–in vivo extrapolations in chemical risk assessment are discussed.

**Electronic supplementary material:**

The online version of this article (10.1007/s00204-020-02765-8) contains supplementary material, which is available to authorized users.

## Article Highlights

- Extensive literature search of human kinetic parameters for UGT probe substrates.
- Bayesian meta-analysis quantifying human variability in acute and chronic kinetic parameters.
- UGT isoform-related uncertainty factors were below the 3.16 kinetic default uncertainty factor for most probe substrates.
- Quantifying human variability in UGT polymorphisms.

**Electronic supplementary material:**

The online version of this article (10.1007/s00204-020-02765-8) contains supplementary material, which is available to authorized users.

## Introduction

Glucuronidation is an enzymatic reaction catalysed by UDP-glucuronosyltransferase (UGT) isoforms which involves the conjugation of endogenous substrates (e.g. bilirubin) and xenobiotics [e.g. pharmaceuticals (morphine), dietary chemicals (flavonoids), and environmental contaminants (mycotoxins)] with glucuronic acid (Dong et al. 2012; Lv et al. 2019). In humans, glucuronide conjugates are water soluble and readily excreted in the urine or the faeces resulting in increased elimination and most often inactivation of the compound, thereby contributing to xenobiotic detoxification (Fisher et al. 2001). Multiple UGT isoforms are often involved in xenobiotic metabolism, which, from a toxicological viewpoint, is advantageous as dysfunctionality of an isoform does not necessarily result in the impaired elimination and thus detoxification of chemicals (Lv et al. 2019). Since UGTs are ubiquitous in pharmacokinetic and toxicokinetic processes [absorption, distribution, metabolism and excretion (ADME)], their involvement in human metabolic variability is important.

The superfamily of UGT isoforms has a nomenclature which is based on similar features to that described for the cytochrome P450 (CYP) superfamily (Meech et al. 2019; Rowland et al. 2013). The subfamilies of UGT1A and UGT2B are predominantly expressed in the liver as well as in the intestine and kidney, where they mediate intestinal and hepatic first-pass glucuronidation of many phenolic compounds, including pharmaceuticals and natural phenols (Dong et al. 2012, Fig. 1). The most clinically relevant hepatic UGTs include UGT1A1, 1A3, 1A4, 1A6, 1A9, 2B7 and 2B15 (Rowland et al. 2013; Stingl et al. 2014). Other UGTs from the 2B subfamily are mainly responsible for the metabolism of endogenous compounds rather than xenobiotics, such as steroids (2B4, 2B15 and 2B17) and bile acids (2B4) (Fisher et al. 2001).

**Fig. 1:**
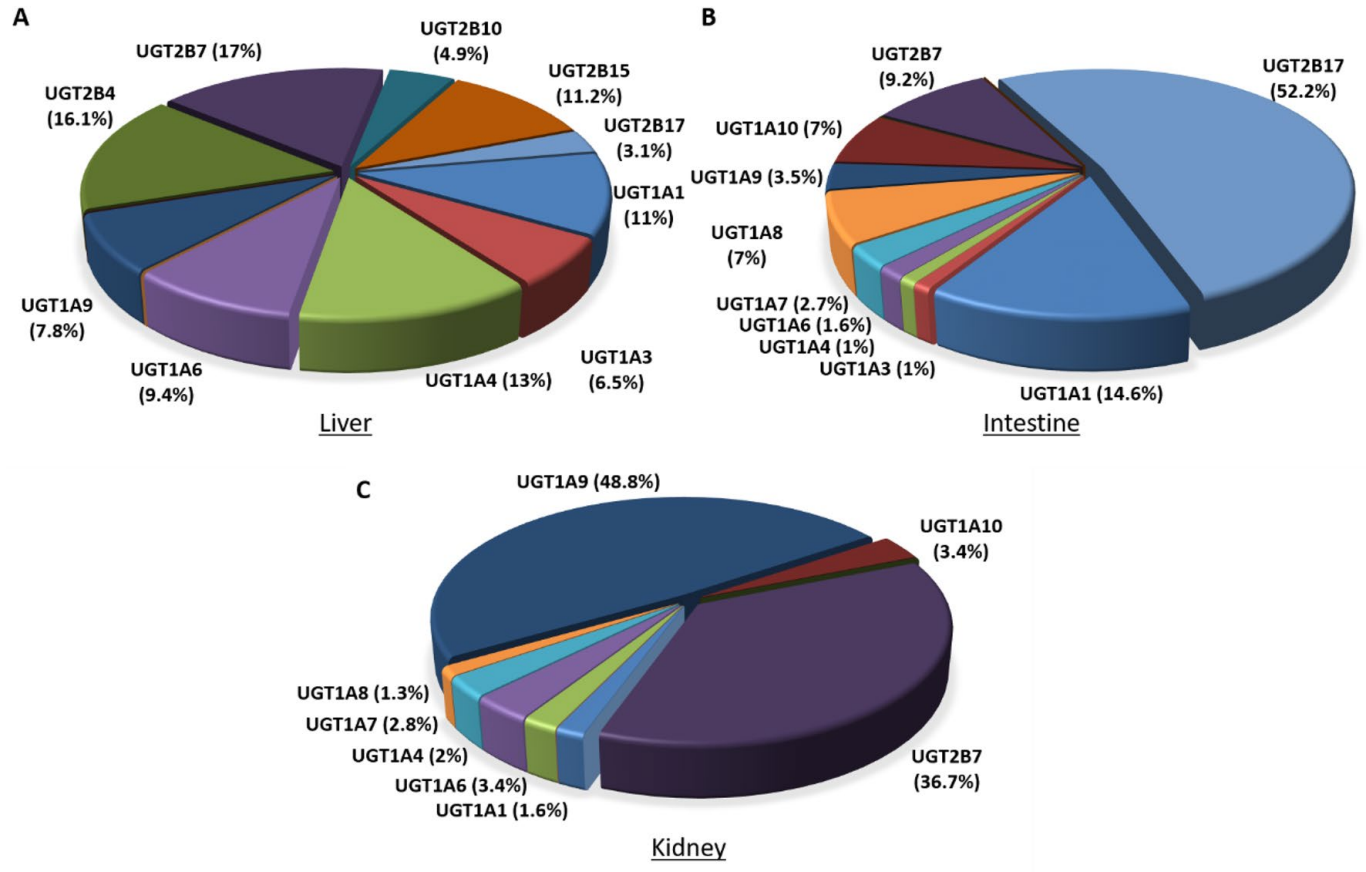
Average distribution of the major UDP-glucuronosyltransferase isoforms in human liver (**a**), intestine (**b**) and kidney (**c**) (Lv et al. 2019)

UGT isoforms are known to be highly polymorphic with more than a hundred variants described (Stingl et al. 2014). In most cases, these polymorphic variants result in lower expression levels and/or lower activity, and in some instances even complete loss of activity (Sim et al. 2013). Because of such changes in expression and/or activity, polymorphic UGT variants may cause higher plasma concentrations of (toxic) metabolites or parent compounds, resulting in chemical-induced toxicity. For example, UGT1A1 polymorphism is associated with irinotecan toxicity, while UGT2B7 polymorphism can affect plasma concentrations of valproic acid (Tsunedomi et al. 2017; Wang et al. 2018b). For other isoforms, comparable impact of UGT polymorphisms on internal drug concentrations has been observed (Stingl et al. 2014).

For the last 70 years, a 100-fold default uncertainty factor (UF) has been applied to derive chronic safe levels of exposure for non-cancer effects of chemicals. This default factor allows for interspecies differences (tenfold) and human variability (tenfold) to chemical exposure. In the 1990s, the tenfold factor allowing for human variability has been refined to a composite value of two factors of 3.16 (10^0.5^), accounting, respectively, for interindividual differences in toxicokinetics and toxicodynamics (Renwick and Lazarus 1998). However, interindividual differences between healthy adults and potentially sensitive subgroups including neonates, elderly and poor metabolisers expressing polymorphic UGT genes may not be covered by the default kinetic factor (Dorne et al. 2001b; Renwick and Lazarus 1998). Under such circumstances, pathway-specific UFs or chemical-specific adjustment factors (CSAFs) have been proposed and can provide an option to replace such default UFs. Pathway-related UFs to account for variability have been described for CYP3A4 as well as efflux and influx transporters (Darney et al. 2019, 2020; Dorne et al. 2001b). Human variability in glucuronidation processes in relation to UFs has been described earlier by Dorne et al. (2001a); however, at the time, information on isoform specificity and genetic polymorphisms was very limited.

**Fig. 2:**
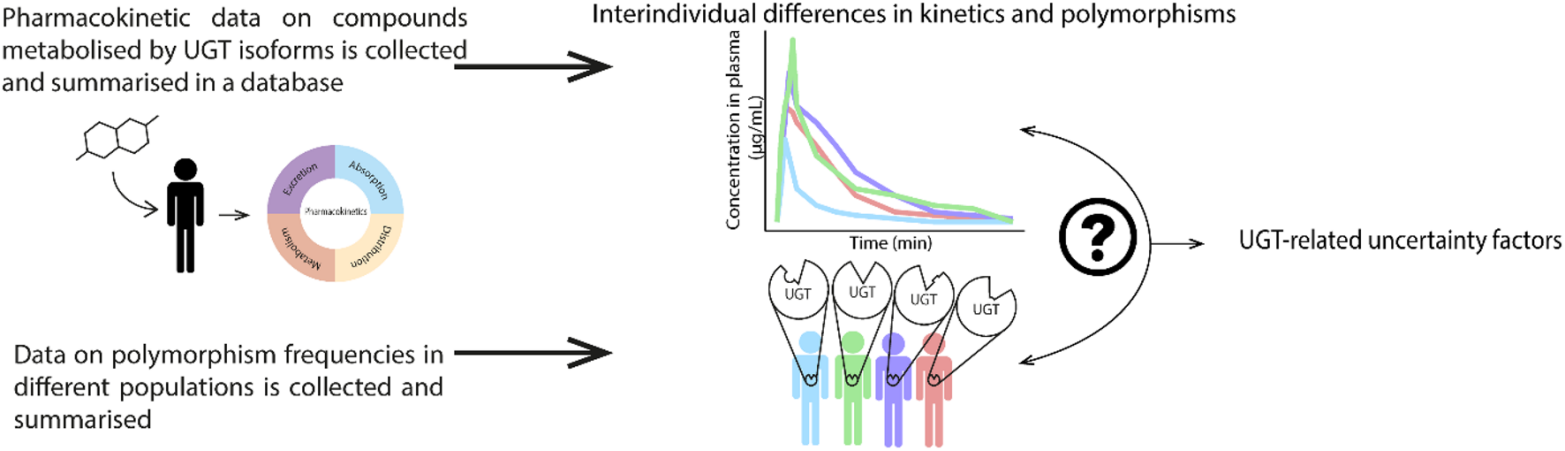
Human variability in the pharmacokinetics of isoform-specific UGT probe substrates, genetic polymorphisms and UGT-related uncertainty factors

The manuscript aims to investigate human variability in UGT activity through (1) identifying isoform-specific UGT probe substrates and collecting pharmacokinetic data for intravenous and oral markers of acute (maximum concentration (Cmax)) and chronic exposure (clearance, area under the curve (AUC)) by means of extensive literature searches and meta-analyses, (2) quantifying interindividual differences in pharmacokinetics by means of hierarchical Bayesian meta-analyses to derive UGT-related variability distributions and UGT-related UFs. Such UGT-related UFs are relevant to refine toxicokinetic UFs for risk assessment of toxicants, nutrients and environmental xenobiotics that are metabolised by UGTs, and (3) unravelling the frequencies and pharmacokinetic consequences of UGT polymorphisms in human populations. A graphical abstract is depicted in Fig. 2.

## Materials and methods

### Extensive literature searches (ELS) and data collection

UGT1A1, 1A3, 1A4, 1A6, 1A9, 2B7 and 2B15 were identified as the most clinically relevant UGT isoforms for xenobiotic metabolism (Rowland et al. 2013; Stingl et al. 2014). Probe substrates for these UGT isoforms were identified from the in vitro and in vivo literature as compounds metabolised by extensive glucuronidation (> 60% of the dose excreted in the urine) (Lv et al. 2019; Rowland et al. 2013; Stingl et al. 2014; Yang et al. 2017).

ELS were performed using two main databases (i.e. Scopus and PubMed) to identify human pharmacokinetic (PK) studies in non-phenotyped adults for isoform-specific UGT probe substrates in adults of different geographical ancestry or ethnic background. A PK database was then computed, including intravenous and oral markers of acute (Cmax) and chronic (clearance and AUC) exposure. Search queries for the probe substrate deferiprone are illustrated in Table 1 and queries for all other substrates are provided in Supplementary Material 1. Data reporting frequencies of UGT polymorphisms distribution and the associated PK parameters in phenotyped individuals were collected using a horizontal literature search in Google Scholar.

**Table 1:** Keyword queries for the extensive literature searches (formatted for Scopus)

General search terms	TITLE-ABS (patient*) OR TITLE-ABS (human) OR TITLE-ABS (adult) OR TITLE-ABS (adults) OR TITLE-ABS (child) OR TITLE-ABS (children) OR TITLE-ABS (infant) OR TITLE-ABS (neonate) OR TITLE-ABS (newborn) OR TITLE-ABS (newborns) OR TITLE-ABS (elderly) OR TITLE-ABS (“pregnant women”) OR TITLE-ABS (men) OR TITLE-ABS (women) OR TITLE-ABS (“ethnic group”) OR TITLE-ABS (caucasian) OR TITLE-ABS (asian) OR TITLE-ABS (african) OR TITLE-ABS (“genetic polymorphism*”) OR TITLE-ABS (“individual susceptibility”) OR TITLE-ABS (“gene environment”) OR TITLE-ABS (“ethnic variability”) OR TITLE-ABS (“Afro American”) OR TITLE-ABS (hispanic) OR TITLE-ABS (“race difference”) OR TITLE-ABS (“age difference”) OR TITLE-ABS (“race differences”) OR TITLE-ABS (“age differences”) OR TITLE-ABS (“gender differences”) OR TITLE-ABS (“gender difference”) OR TITLE-ABS (“sex difference”) OR TITLE-ABS (“sex differences”)
Search terms for probe substrates	(TITLE-ABS (deferiprone) OR TITLE-ABS (ferriprox))
Search terms for pharmacokinetics	TITLE-ABS-KEY (auc) OR TITLE-ABS-KEY (area AND under AND the AND curve) OR TITLE-ABS-KEY (area AND under AND curve) OR TITLE-ABS-KEY (half AND life) OR TITLE-ABS-KEY (half-life) OR TITLE-ABS-KEY (half-lives) OR TITLE-ABS-KEY (clearance) OR TITLE-ABS-KEY (cmax) OR TITLE-ABS-KEY (vmax) OR TITLE-ABS-KEY (km) OR TITLE-ABS-KEY (“michaelis constant”) OR TITLE-ABS-KEY (pharmacokinetic) OR TITLE-ABS-KEY (pharmacokinetics) OR TITLE-ABS-KEY (toxicokinetic) OR TITLE-ABS-KEY (toxicokinetics)
Exclusion	TITLE-ABS-KEY (“cell line*”) OR TITLE-ABS-KEY (“cell culture*”) OR TITLE-ABS-KEY (rat) OR TITLE-ABS-KEY(rats) OR TITLE-ABS-KEY (mouse) OR TITLE-ABS-KEY (mice)

*TITLE-ABS-KEY* term searched in the title, the abstract and the keywords of the paper

A two-step process was conducted to screen the retrieved studies from literature as described previously (Darney et al. 2019). This process was used to assess whether reported PK values were suitable for inclusion in the database. After removing duplicates, the following exclusion criteria were applied: 1. species other than humans, 2. in vitro studies, 3. development of analytical methods, 4. modelling studies, 5. pharmacodynamics investigations only, 6. substrates other than those identified as relevant and/or mixtures of substrates. Articles meeting the exclusion criteria were not considered for further evaluation. Furthermore, articles that were written in any other language than English or did not contain original research data (e.g. reviews) were excluded from analysis. Overall, data on non-phenotyped healthy individuals were collected and included in the meta-analysis (see “Data standardisation and meta-analyses”). The specific selection of subgroups is described in Supplementary Material 1. In a second step, the full text of the included papers was checked for PK parameter values after single-dose exposure. Repeated dosing studies and studies for which multiple formulas were administered to the same group of volunteers were excluded. However, for ethinylestradiol, data were included for both single dose and repeated dosing for 21 days, the standard regimen for anticonception drugs.

### Data standardisation and meta-analyses

Meta-analyses of PK parameter values were performed in non-phenotyped subjects for each probe substrate to derive UGT-related variability distributions and UGT-related UFs. For this purpose, each PK parameter was normalised in a harmonised manner (Cmax expressed in ng/mL; AUC in ng*h/mL and clearance in L/h/kg bw) while applying body weight correction to the applied doses (mg/kg bw). If available, the reported (mean) body weight was used, or continent specific body weights were used to normalise the dose if body weight data were not available (Walpole et al. 2012). For SN38, the dose was normalised to body surface area instead of body weight, as this is the standard measure for this compound. If body surface area data were not available, a default value of 1.79 m^2^ was used (Sacco et al. 2010). Data from these studies were extracted mostly as arithmetic mean (AM) and standard deviation (SD), but sometimes geometric means (GM) and geometric standard deviations (GSD) were reported. Generally, PK data are recognised to follow a lognormal distribution (Dorne et al. 2001b; Naumann et al. 1997; Renwick and Lazarus 1998). Since GM and GSD are more appropriate to summarise a lognormal distribution, all pharmacokinetic data were described as GM and GSD using the following equations:

1 $${\text{GM}} = \frac{X}{{\sqrt {1 + {\text{ CV}}_{N}^{2} } }},$$

2 $${\text{GSD}} = {\exp}\left( {\sqrt {\ln \left( {1 + {\text{CV}}_{N}^{2} } \right)} } \right),$$

where *X* is the arithmetic mean and CV_*N*_ is the coefficient of variation for normally distributed data:

3 $${\text{CV}}_{N} = \frac{{{\text{SD}}}}{X}.$$

In some studies, SD was not reported and was estimated from the standard error (SE, SEM) or CV using equations described previously (Darney et al. 2019).

The objective of the meta-analyses is to provide accurate information regarding interindividual differences in non-phenotyped adults of the PK parameters for a substrate expressed as distributions. Variability related to interstudy, intersubstrate and interindividual differences was analysed for each substrate and parameter and for each UGT isoform, through a decomposition of the PK parameter variance (clearance, AUC or Cmax) using a previously described hierarchical Bayesian model (Darney et al. 2019; Wiecek et al. 2019). For the meta-analysis, non-informative prior data were selected for most compounds, except for zidovudine and oxazepam for which kinetic variability was previously meta-analysed (Dorne et al. 2001a).

Overall, the meta-analyses provided variability and uncertainty distributions describing interindividual differences for each PK parameter using median values and 95% confidence intervals. The coefficient of variation (CV) was also estimated as follows:

4 $${\text{CV}} = \sqrt {{\exp}\left( {\ln \left( {\sqrt {{\exp}(1/{\uptau }_{j} } } \right)} \right)^{2} - 1} ,$$

where ${\uptau }_{j}$ is the interindividual difference of the activity for a substrate ‘*j*’.

UGT isoform-related UFs were calculated as the ratio between the percentile of choice and the median of the distribution for each PK parameter for 95th and 97.5th centiles.

### Software

All statistical analyses were performed in R (version 3.5) and the Bayesian modelling was implemented in Jags (4.2.0) (Plummer 2003). Data processing and graphical display were performed in R (dplyr and ggplot2 packages) (R Development Core Team 2018; Wickham 2016; Wickham et al. 2020). References of the studies used to compile the database were stored and sorted in EndNote X8.

## Results and discussion

### Extensive literature searches and data collection

UGT isoforms can conjugate a wide variety of substrates and show a broad overlapping substrate specificity. This is advantageous when detoxifying chemicals; however, because of such overlap, identifying selective probe substrates for each isoform remains a challenge. Moreover, UGTs are also present in the gastrointestinal tract and pre-systemic conjugation occurs readily for a range of compounds. Here, to quantify isoform-specific variability in UGTs, selective probe substrates with available PK data for each isoform were selected. Moreover, differences in variability between oral and intravenous data were compared to investigate the contribution of bioavailability and pre-systemic conjugation after oral intake. A total of 14 isoform-specific UGT probe substrates covering both the UGT1A and UGT2B subfamilies were identified, namely 1-OH-midazolam (UGT1A4), codeine (UGT2B7), deferiprone (UGT1A6), entacapone (UGT1A9), ethinylestradiol (UGT1A1), ezetimibe (UGT1A1/UGT1A3), mycophenolic Acid (UGT1A9), oxazepam (UGT2B15/UGT1A9), propofol (UGT1A9), raltegravir (UGT1A1), SN38 (UGT1A1), telmisartan (UGT1A3), trifluoperazine (UGT1A4) and zidovudine (UGT2B7).

**Fig. 3:**
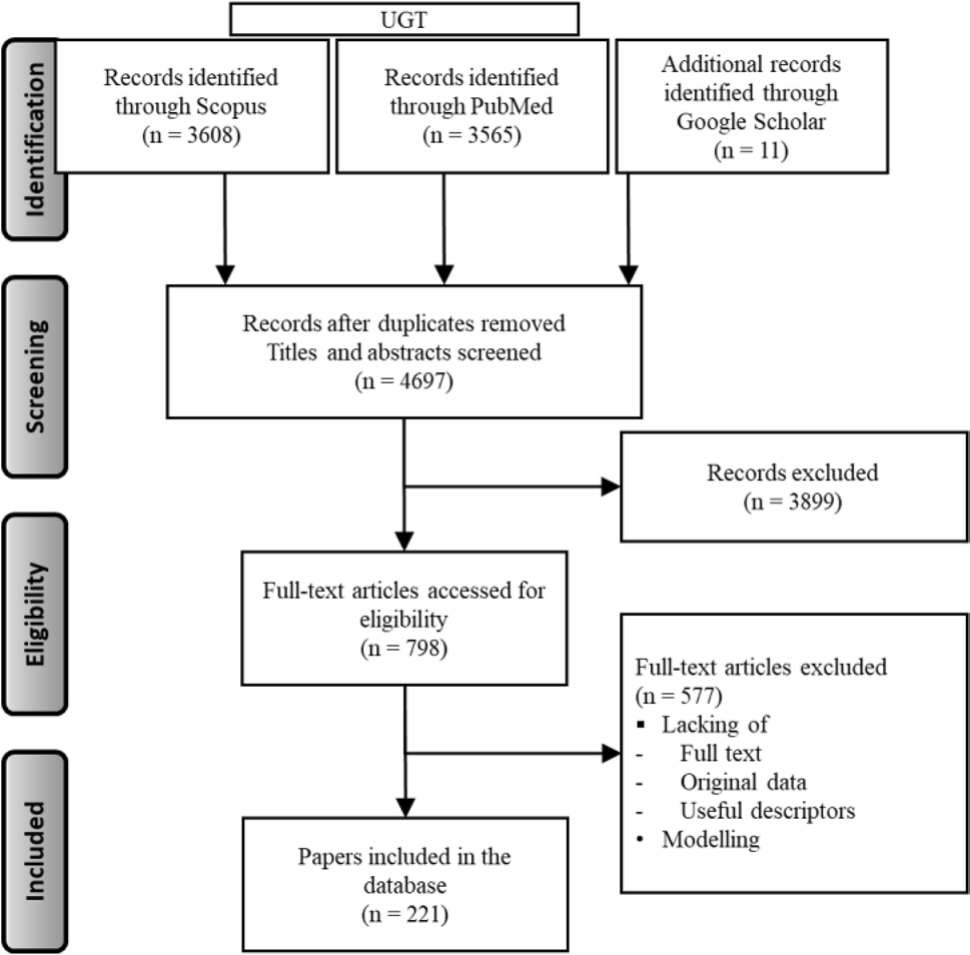
PRISMA diagram illustrating the extensive literature searches performed for the 13 isoform-specific UGT probe substrates (UGT1A and UGT2B subfamilies) and human pharmacokinetic studies

From the ELS, a total of 7173 papers were assessed from Scopus and PubMed (up to August 2019) and an extra 11 papers were retrieved from Google Scholar, for the 13 UGT isoform probe substrates and for zidovudine, 10 studies were included from a previous database (shared by Dr. N. Quinot, collated for EFSA/SCER/2014/06 project). PRISMA flow diagrams for the individual compounds are provided in Supplementary Material 2. Figure 3 provides a summary PRISMA diagram for all papers collected in the ELS (Moher et al. 2009). Overall, a total of 210 peer-reviewed publications were selected from the ELS and included in the database. Supplementary Material 1 provides the search queries for both Scopus and PubMed for the individual compounds. Table 2 illustrates the selected probe substrates, the structure of the compounds, bioavailability, percentage of glucuronidation and their site of glucuronidation.

**Table 2:**
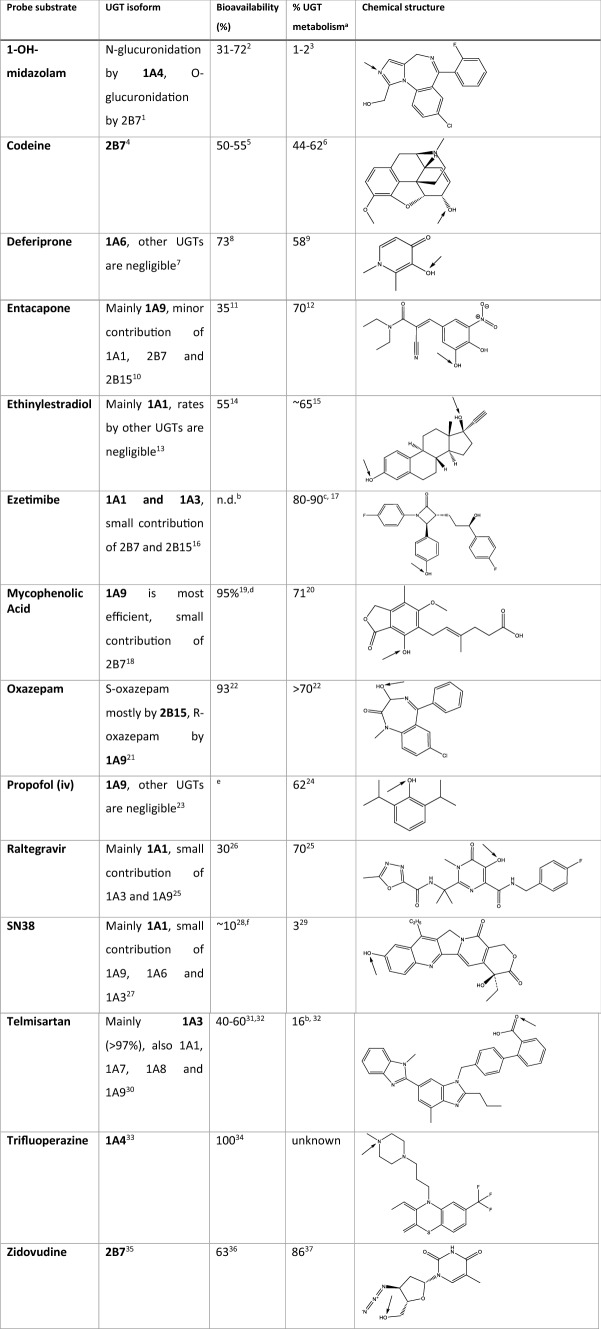
Isoform-specific UGT probe substrates

Name of probe substrate, major UGT isoform involved in glucuronidation (in bold), % bioavailability, % of dose metabolised by UGT and chemical structure are reported. Arrows indicate the main site(s) of glucuronidation

^1^Seo et al. (2010), ^2^Heizmann et al. (1983), ^3^Hyland et al. (2009), ^4^Coffman et al. (1997), ^5^Rogers et al. (1982), ^6^Yue et al. (1989), ^7^Benoit-Biancamano et al. (2009a), ^8^ClinicalTrials.gov (2014), ^9^Rodrat et al. (2012), ^10^Lautala et al. (2000), ^11^Heikkinen et al. (2001), ^12^Wikberg et al. (1993), ^13^Ebner et al. (1993) and Lv et al. (2019),^14^Fotherby (1996), ^15^Williams and Goldzieher (1980), ^16^Ghosal et al. (2004), ^17^Kosoglou et al. (2005), ^18^Picard et al. (2005),^19^Armstrong et al. (2005), ^20^Bullingham et al. (1996), ^21^Court et al. (2002), ^22^Sonne et al. (1988), ^23^Seo et al. (2014), ^24^Favetta et al. (2002), ^25^Kassahun et al. (2007) fraction of dose metabolized by UGT1A1, ^26^Brainard et al. (2011), ^27^Hanioka et al. (2001), ^28^Furman et al. (2009), ^29^Slatter et al. (2000), ^30^Yamada et al. (2011), ^31^Wienen et al. (2000), ^32^Stangier et al. (2000a), remainder is unchanged parent compound, ^33^Seo et al. (2014), ^34^Midha et al. (1984), ^35^Barbier et al. (2000), ^36^Klecker et al. (1987), ^37^Gallicano et al. (1999)

^a^Expressed as % of the dose recovered in urine as the glucuronide, ^b^n.d. = not determined. The bioavailability of ezetimibe cannot be determined, because it is insoluble in aqueous media and cannot be used for intravenous injection (Kosoglou et al. 2005), ^c^Expressed as % of dose as glucuronide in plasma, ^d^Mycophenolic acid is given as a prodrug, mycophenolate mofetil, ^e^No bioavailability is given for propofol, as all studies in the database are intravenous studies, ^f^Bioavailability of irinotecan, the parent drug of SN38

### Interindividual differences in the kinetics of isoform-specific UGT probe substrates and related uncertainty factors in non-phenotyped adults

**Fig. 4:**
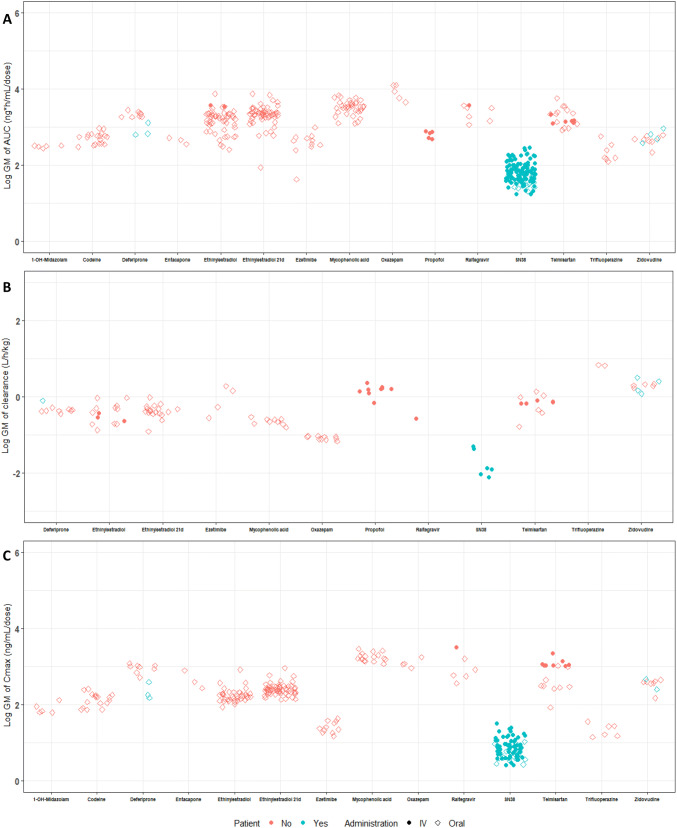
Results of the meta-analyses reporting interindividual differences in non-phenotyped healthy adults for 14 isoform-specific UGT probe substrates. Data are expressed as log geometric means (GM) for markers of acute (Cmax) and chronic (AUC, clearance) exposure. AUC (normalised to dose, **a**), clearance (normalised to body weight, **b**), and Cmax (normalised to dose, **c**). Open squares: oral exposure; solid circles: IV exposure. Red data points: healthy volunteers; blue data points: patients. 21d: repeated dose for 21 days

**Fig. 5:**
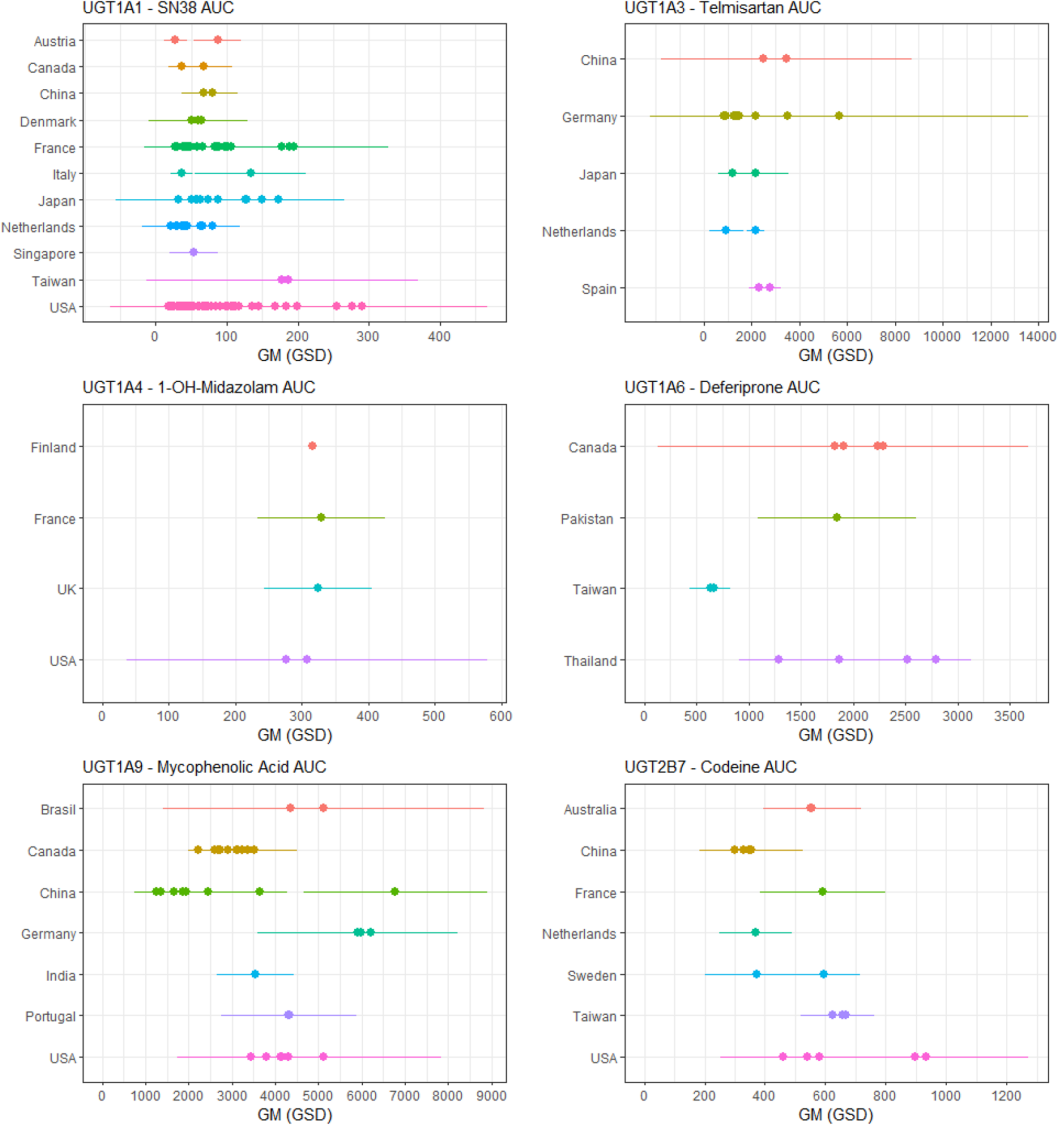
Inter-individual differences in markers of chronic exposure (area under the concentration–time curve (AUC)) for isoform-specific UGT probe substrates across world populations of different geographical ancestry. For each UGT isoform included in the study, one probe substrate is shown. Graphs for other probe substrates and other PK parameters are accessible in Supplementary Material 3

Results of the meta-analyses are expressed as geometric means (normalised to dose and body weight) for the 14 isoform-specific UGT probe substrates and are illustrated for markers of acute (Cmax) and chronic (AUC/clearance) exposure after oral and intravenous administration in Fig. 4. The full dataset of extracted information used can be accessed on EFSA knowledge junction (Kasteel et al. 2020). Data availability was variable for each UGT probe substrate and interstudy differences are reported for each compound. For SN38, only patient data were available and no data on healthy adult individuals were reported in the literature. For deferiprone, no clear distinction could be made between healthy adult data and patient data for all three parameters, suggesting that the condition of the individuals did not influence the pharmacokinetics of this compound. In Fig. 5, isoform-specific interindividual differences in AUC are illustrated for world populations from different geographical ancestry or country of origin (one probe substrate per UGT isoform). These plots indicate that no clear differences in chronic exposure (AUC) can be demonstrated across world populations from different geographical ancestry and, therefore, there is indication of significant interethnic differences for these probe substrates. The same conclusion holds for other PK parameters and other probe substrates, which are illustrated in Supplementary Material 3.

Interindividual differences were higher compared to an earlier study (Dorne et al. 2001a), which included 11 probe substrates compared to 14 here; with an overlap of only two probe substrates (zidovudine (AZT) and oxazepam). In addition, Dorne et al. (2001a) mostly considered UGT2B7 probe substrates while UGT1A1-specific probe substrates were not included since they were not available at that time. Polymorphisms have the highest impact on the PK of UGT1A1 probe substrates, which may explain the larger interindividual differences in this study. Finally, the 2001 study investigated PK data for potentially sensitive subgroups including neonates, infants, children, and the elderly, but little-to-no data for these subgroups were available for the included probe substrates here (Dorne et al. 2001a). It is worth noting that UGT metabolism in neonates is impaired and that they show a low glucuronidation activity (Allegaert et al. 2009). Data for such PK differences in markers of acute and chronic exposure are still very limited for these subgroups, but can reach two- to threefold in comparison with healthy adults so that the default kinetic factor may be inadequate and an extra UF may be required to cover these subgroups (Dorne et al. 2001b, 2005).

#### UGT1A1

For UGT1A1, ethinylestradiol, ezetimibe, raltegravir and SN38 were identified as probe substrates. Besides pharmaceuticals, UGT1A1 is involved in the glucuronidation of several compounds important in (food) toxicology, including the naturally occurring food components resveratrol and several hydroxyflavones, the heterocyclic amine 2-amino-1-methyl-6-phenylimidazo[4,5-b]pyridine (PhIP) and the phytochemical ferulic acid (Brill et al. 2006; Li et al. 2011; Malfatti and Felton 2004; Tang et al. 2010). For ethinylestradiol, only single-dose studies were used to quantify the UGT1A1 variability. PK data were available for Europeans, East Asians, South Asians, Southeast Asians, North Americans, South Americans, North Africans and Middle Eastern adults with the majority of the datasets from North American and European studies. Data gaps were identified for specific groups like Central Americans and Africans. Chemical-specific CVs ranged from 35 to 72%, while isoform-related CVs ranged from 46 to 51% (Table 3). Overall, the UGT1A1 related UFs were most often below or close to the default TK UF of 3.16 for at least 97.5% of the healthy adults when considering the median value. However, our analysis by the Bayesian model takes into account the uncertainty around the estimation of the UF and this shows that, given the available data (number of studies and number of individuals per study), the default factor may not cover all possible cases. Indeed, the upper bound of the confidence interval is higher than 3.16. The chemical-specific data show that SN38, ezetimibe and raltegravir all have an UF_97.5_ higher than 3.16, ranging from 3.2 to 3.6 (Table 3). Ethinylestradiol is the only UGT1A1 probe substrate studied here that did not exceed the default kinetic UF for any of the parameters.

**Table 3:** Pharmacokinetic parameters of UGT1A1 probe substrates in non-phenotyped adults after oral or intravenous administration

Route	Parameter	Compound	nst	*n*	CV	GM	UF95	95% CI	UF97.5	95% CI
Oral	AUC (ng*h/mL/dose)	Ethinylestradiol^a^	60	1236	41	2045	1.9	1.8–2.1	2.2	2.0–2.4
		Ethinylestradiol^b^	50	974	42	1355	1.9	1.8–2.1	2.2	2.0–2.5
		Ezetimibe	11	173	44	356	2.0	1.7–2.4	2.3	1.9–2.9
		Raltegravir	6	67	60	2110	2.5	1.9–3.8	3.0	2.2–4.9
		SN38	20	139	70	8039	2.8	2.2–3.9	3.5	2.6–5.0
		**Overall (*n*** **= 4)**	**147**	**2589**	**50**		**2.2**	**1.7–3.6**	**2.5**	**2.0–4.6**
Oral	Clearance (mL/min/kg)	Ethinylestradiol^a^	19	324	36	6.8	1.8	1.6–2.0	2.0	1.8–2.3
		Ethinylestradiol^b^	11	135	38	6.3	1.8	1.6–2.3	2.1	1.7–2.8
		Ezetimibe	4	55	66	13.9	2.7	2.0–4.7	3.3	2.2–6.2
		**Overall (*n* = 2)**	**34**	**514**	**48**		**2.1**	**1.6–4.2**	**2.5**	**1.7–5.5**
Oral	Cmax (ng/mL/dose)	Ethinylestradiol^a^	39	1295	35	250	1.7	1.6–1.9	1.9	1.8–2.1
		Ethinylestradiol^b^	63	841	38	175	1.8	1.7–2.0	2.1	1.9–2.3
		Ezetimibe	11	173	47	25.8	2.1	1.8–2.5	2.4	2.0–3.0
		Raltegravir	5	56	72	594	2.9	2.1–5.1	3.6	2.4–7.0
		SN38	20	146	64	5.0	2.6	2.1–3.5	3.2	2.5–4.5
		**Overall (*n* = 4)**	**138**	**2511**	**53**		**2.3**	**1.7–4.1**	**2.7**	**1.9–5.3**
Intravenous	AUC (ng*h/mL/dose)	Ethinylestradiol	2	24	39	3585	1.9	1.4–3.4	2.1	1.5–4.3
		Raltegravir	1	3	37	3752	1.8	1.5–2.6	2.0	1.6–3.1
		SN38	109	1407	62	67.4	2.5	2.3–2.8	3.0	2.7–3.5
		**Overall (*n* = 3)**	**112**	**1434**	**46**		**2.1**	**1.5–2.8**	**2.4**	**1.6–3.5**
Intravenous	Clearance (mL/min/kg)	Ethinylestradiol	3	33	39	4.8	1.9	1.5–3.0	2.1	1.6–3.7
		Raltegravir	1	3	38	4.5	1.9	1.2–9.2	2.1	1.2–13
		SN38	6	79	68	0.32	2.8	2.1–4.4	3.4	2.4–5.8
		**Overall (*n* = 3)**	**10**	**115**	**51**		**2.6**	**1.3–5.8**	**2.6**	**1.3–7.7**

*nst* number of studies, *n* number of subjects

^a^Repeated dosing of 21 days

^b^Single dose

#### UGT1A3

UGT1A3 is a UGT isoform involved in the glucuronidation of the flavonoid icaritin and several other flavonoids (Chen et al. 2008; Wang et al. 2018a). In this study, two probe substrates were included for UGT1A3, namely telmisartan and ezetimibe. Isoform-related CVs varied from 37–62%. Highest variability was observed for telmisartan and ezetimibe clearance with CV values of 59 and 66%, respectively. It has been demonstrated previously in the literature that telmisartan shows high variability in PK parameters (Chen et al. 2013; Deppe et al. 2010; Kang et al. 2018; Stangier et al. 2000b). Overall, UGT1A3-related UFs were below the default TK UF of 3.16 (Table 4).

**Table 4:** Pharmacokinetic parameters for UGT1A3 probe substrates in non-phenotyped adults after single-dose oral or intravenous administration

Route	Parameter	Compound	nst	*n*	CV	GM	UF95	95% CI	UF97.5	95% CI
Oral	AUC (ng*h/mL/dose)	Ezetimibe	11	173	44	356	2.0	1.7–2.4	2.3	1.9–2.9
		Telmisartan	13	225	53	2155	2.2	1.9–2.7	2.6	2.2–3.3
		**Overall (*n* = 2)**	**24**	**398**	**49**		**2.1**	**1.8–2.7**	**2.4**	**2.0–3.2**
	Clearance (mL/min/kg)	Ezetimibe	4	55	66	13.9	2.7	2.0–4.7	3.3	2.2–6.2
		Telmisartan	6	103	59	10.1	2.5	2.0–3.5	2.9	2.2–4.4
		**Overall (*n* = 2)**	**10**	**158**	**62**		**2.6**	**2.0–4.2**	**3.1**	**2.2–5.6**
	Cmax (ng/mL/dose)	Ezetimibe	11	173	47	25.8	2.1	1.8–2.5	2.4	2.0–3.0
		Telmisartan	9	144	38	391	1.8	1.6–2.2	2.0	1.7–2.6
		**Overall (*n* = 2)**	**20**	**317**	**43**		**2.0**	**1.6–2.4**	**2.2**	**1.8–2.9**
Intravenous	AUC (ng*h/mL/dose)	Telmisartan^a^	6	41	37	1469	1.8	1.5–2.6	2.0	1.6–3.2
	Clearance (mL/min/kg)	Telmisartan^a^	5	36	39	12.2	1.9	1.5–2.9	2.1	1.6–3.6

*nst* number of studies,* n* number of subjects

^a^1 compound: considered as overall

#### UGT1A4

The ginsenoside 20(S)-protopanaxadiol is one of the naturally occurring probe substrates of the UGT1A4 isoform (Li et al. 2016). UGT1A4 probe substrates selected here included trifluoperazine and 1-OH-midazolam. It is important to note that 1-OH-midazolam is a metabolite of midazolam which is formed by CYP3A4 and then conjugated by UGT1A4. Variability for trifluoperazine was quite extensive, although only a limited number of publications were available, and studies were all from Canada. Large interindividual differences in PK parameters has previously been demonstrated for trifluoperazine, independent of ethnicity (Midha et al. 1988). After oral administration, 1A4 shows the highest variability regarding acute exposure (Cmax) out of all isoforms with a CV of 62%. However, least variability was found for UGT1A4 in mRNA expression levels when compared with mRNA expression levels of UGT1A1, UGT1A3, UGT1A6 and UGT1A9 (Aueviriyavit et al. 2007). Despite this low variability in mRNA expression levels, an exceedance of the default TK UF is observed for the 97.5th percentile for 1-OH-midazolam (UF_97.5_: 3.3, Table 5).

**Table 5:** Pharmacokinetic parameters of UGT1A4 probe substrates in non-phenotyped adults after single-dose oral or intravenous administration

Route	Parameter	Compound	nst	*n*	CV	GM	UF95	95% CI	UF97.5	95% CI
Oral	AUC (ng*h/mL/dose)	1-OH-midazolam	5	67	35	308	1.7	1.5–2.3	1.9	1.6–2.6
		Trifluoperazine	7	75	64	207	2.6	2.0–4.0	3.2	2.3–5.3
		**Overall (*n* = 2)**	**12**	**142**	**47**		**2.1**	**1.5–3.7**	**2.4**	**1.6–4.8**
Oral	Clearance (mL/min/kg)	Trifluoperazine^a^	2	48	57	112	2.4	1.8–4.0	2.8	2.0–5.1
Oral	Cmax (ng/mL/dose)	1-OH-midazolam	5	67	67	76	2.7	2.0–4.3	3.3	2.3–5.7
		Trifluoperazine	6	79	58	18.3	2.4	1.9–3.5	2.9	2.2–4.5
		**Overall (*n* = 2)**	**11**	**146**	**62**		**2.6**	**2.0–4.0**	**3.1**	**2.2–5.3**

*nst* number of studies, *n* number of subjects

^a^1 compound: considered as overall

#### UGT1A6

Of the seven UGT isoforms investigated in this study, UGT1A6 has been recognised as one of the minor isoforms for glucuronidation and drug metabolism (Stingl et al. 2014). However, it is involved in the glucuronidation of several pharmaceuticals, including acetaminophen and aspirin, and the remarkable sensitivity of cats to these analgesics is due to the lack of UGT1A6 expression in the feline liver (Shrestha et al. 2011). The natural occurring compound protocatechuic aldehyde is also metabolised by this UGT isoform (Liu et al. 2008). In this study, deferiprone was included as probe substrate for UGT1A6. Only data after oral administration were available and for all PK parameters, the CVs ranged from 36 to 48% Table 6) with UGT1A6-related UFs all below the default TK UF.

**Table 6:** Pharmacokinetic parameters of UGT1A6 probe substrates in non-phenotyped adults after single-dose oral or intravenous administration

Route	Parameter	Compound	nst	*n*	CV	GM	UF95	95% CI	UF97.5	(95% CI)
Oral	AUC (ng*h/mL/dose)	Deferiprone^a^	11	101	36	1654	1.8	1.5–2.2	2.0	1.7–2.5
Oral	Clearance (mL/min/kg)	Deferiprone^a^	9	89	40	1.9	1.9	1.6–2.4	2.1	1.7–2.9
Oral	Cmax (ng/mL/dose)	Deferiprone^a^	11	101	48	616	2.1	1.7–2.8	2.4	1.9–3.4

*nst* number of studies, *n* number of subjects

^a^1 compound: considered as overall

#### UGT1A9

For the UGT1A9 isoform, several relevant substrates include resveratrol, several flavonols and the natural flavouring agent estragole (Brill et al. 2006; Iyer et al. 2003; Wu et al. 2011). Probe substrates for this isoform included entacapone, mycophenolic acid, oxazepam, and propofol. Overall, isoform-related CVs varied between 23 and 41%. For oxazepam, variability in PK parameters was described previously (Dorne et al. 2001a). Compared to our results, variability in Cmax and AUC was comparable, while the calculated variability was lower for clearance in our study (33% against 51%). UGT1A9-related UFs did not exceed the UF of 3.16 (Table 7).

**Table 7:** Pharmacokinetic parameters of UGT1A9 probe substrates in non-phenotyped adults after single dose oral or intravenous administration

Route	Parameter	Compound	nst	*n*	CV	GM	UF95	95% CI	UF97.5	95% CI
Oral	AUC (ng*h/mL/dose)	Entacapone	3	56	28	442	1.6	1.4–2.0	1.7	1.5–2.3
		Mycophenolic acid	35	837	30	3241	1.6	1.5–1.7	1.8	1.6–1.9
		Oxazepam	5	44	44	8039	2.0	1.6–3.0	2.3	1.7–3.7
		**Overall (*n* = 3)**	**43**	**937**	**31**		**1.6**	**1.4–2.6**	**1.8**	**1.5–3.2**
Oral	Clearance (mL/min/kg)	Mycophenolic acid	10	140	41	3.7	1.9	1.6–2.4	2.2	1.8–2.8
		Oxazepam	10	86	33	1.4	1.7	1.5–2.1	1.9	1.6–2.4
		**Overall (*n* = 2)**	**20**	**226**	**37**		**1.8**	**1.5–2.3**	**2.0**	**1.6–2.7**
Oral	Cmax (ng/mL/dose)	Entacapone	3	56	48	447	2.1	1.7–3.1	2.4	1.9–3.9
		Mycophenolic acid	17	583	43	1818	2.0	1.8–2.2	2.2	2.0–2.5
		Oxazepam	4	35	26	1243	1.5	1.3–2.1	1.6	1.4–2.4
		**Overall (*n* = 3)**	**24**	**674**	**41**		**1.9**	**1.3–2.8**	**2.2**	**1.4–3.3**
Intravenous	AUC (ng*h/mL/dose)	Propofol^a^	5	43	31	635	1.7	1.4–2.3	1.8	1.5–2.7
Intravenous	Clearance (mL/min/kg)	Propofol^a^	9	79	23	24.7	1.5	1.3–1.7	1.6	1.4–1.9

*nst* number of studies, *n* number of subjects

^a^1 compound: considered as overall

#### UGT2B7

UGT2B7 is a UGT isoform which conjugates natural compounds such as emodin, a Chinese traditional medicine, the natural sweetener stevioside and natural compounds from herbs such as andrographolide and estragole (Iyer et al. 2003; Tian et al. 2015; Wang et al. 2014; Wu et al. 2018). Selective pharmaceutical probe substrates included in this study were codeine and zidovudine and isoform-related CVs varied between 26 and 37% (Table 8). The PK database mainly consisted of Caucasians (North America and Europe) for both compounds. For codeine, five studies from the USA and four studies from Europe were available, and the remaining studies were from Asia or Australia. For zidovudine, six studies were available from North America, and three from South America and Europe. The variability as indicated by the CV was 26% for clearance, 28% for AUC, and 43% for the Cmax for zidovudine. While the calculated variability for clearance and Cmax is comparable to Dorne et al. (2001a), the AUC showed less variability (28%, 12 studies against 56%, 2 studies). UGT2B7-related UFs did not exceed the TK default UF.

**Table 8:** Pharmacokinetic parameters of UGT2B7 probe substrates in non-phenotyped adults after single-dose oral or intravenous administration

Route	Parameter	Compound	nst	*n*	CV	GM	UF95	95% CI	UF97.5	95% CI
Oral	AUC (ng*h/mL/dose)	Codeine	18	209	29	510	1.6	1.5–1.8	1.7	1.6–2.0
		Zidovudine	12	107	28	477	1.6	1.4–1.8	1.7	1.5–2.1
		**Overall (*n* = 2)**	**30**	**316**	**28**		**1.6**	**1.4–1.8**	**1.7**	**1.5–2.0**
Oral	Clearance (mL/min/kg)	Zidovudine^a^	9	72	26	33.3	1.5	1.4–1.8	1.7	1.4–2.1
Oral	Cmax (ng/mL/dose)	Codeine	17	192	33	134	1.7	1.5–1.9	1.9	1.6–2.2
		Zidovudine	9	94	43	344	2.0	1.7–2.6	2.3	1.8–3.1
		**Overall (*n* = 2)**	**26**	**286**	**37**		**1.8**	**1.5–2.5**	**20**	**1.7–2.9**

*nst* number of studies, *n* number of subjects

^a^1 compound: considered as overall

#### UGT2B15

UGT2B15 is mostly responsible for the metabolism of endogenous compounds such as steroids (e.g. dihydrotestosterone and 17β-diol) (Chen et al. 1993). Environmental contaminants that are metabolised by UGT2B15 include bisphenol A (Hanioka et al. 2008). Major xenobiotic substrates for UGT2B15 include the pharmaceuticals lorazepam and S-oxazepam, although lorazepam is not recommended as a probe substrate for the isoform because of the involvement of several other UGT isoforms in its glucuronidation (Lv et al. 2019; Rowland et al. 2013). Oxazepam is a benzodiazepine which is administered as a racemic mixture, with R-oxazepam being glucuronidated by UGT1A9 and S-oxazepam being glucuronidated by UGT2B15. Variability in the ratio between the R-glucuronide and the *S*-glucuronide has been characterized particularly for the formation of the *S*-glucuronide (Patel et al. 1995). Table 7 shows that variability in oxazepam is 33% and 44% for markers of chronic exposure and 26% for markers of acute exposure and all calculated UGT2B15-related UFs are below the default TK UF. As oxazepam is the only substrate included for UGT2B15, calculated CVs and UFs for oxazepam are considered the overall isoform-specific CVs and UFs for UGT2B15.

### Frequencies of UGT isoform polymorphisms in world populations and impact on the pharmacokinetics of probe substrates in non-phenotyped subjects

**Fig. 6:**
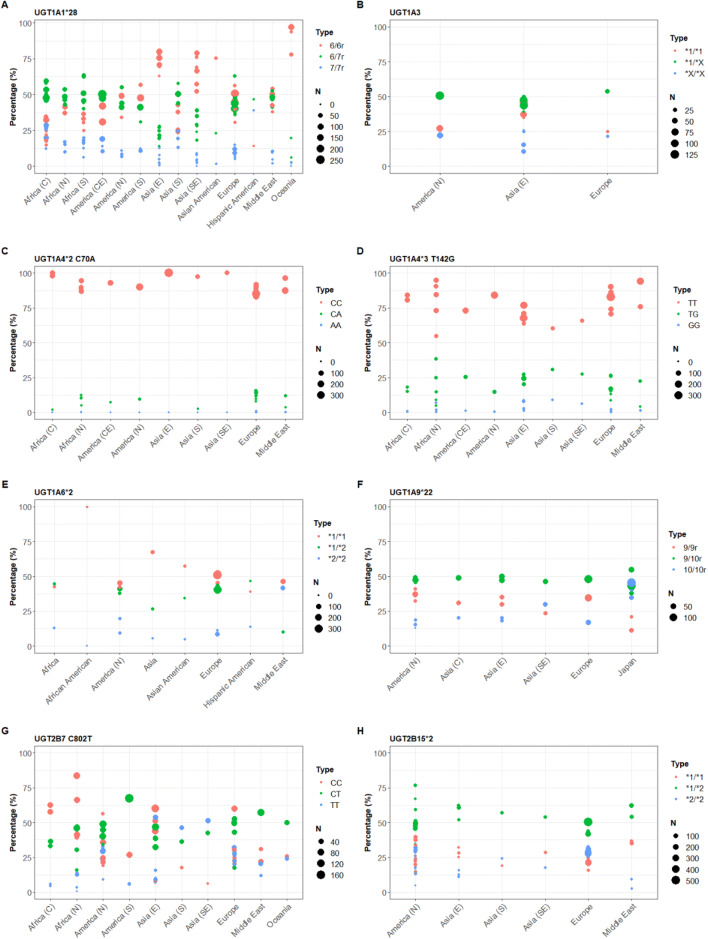
Frequencies of single nucleotide polymorphisms (SNPs) genotypes for UGT1A1*28 (**a**), UGT1A3 (**b**), UGT1A4*2 (C70A) (**c**) UGT1A4*3 (T142G) (**d**), UGT1A6*2 (**e**), UGT1A9*22 (**f**), UGT2B7 (C802T) (**g**), and UGT2B15*2 (**h**) in human populations of different geographical ancestries. *C(E)* central, *N* north, *S* south, *E* east, *SE* southeast

Frequencies of single nucleotide polymorphisms (SNPs) of UGT isoforms, namely UGT1A1*28, UGT1A3, UGT1A4*2 (C70A), UGT1A4*3 (T142G), UGT1A6*2, UGT1A9*22, UGT2B7 C802T and UGT2B15*2 are presented in Fig. 6 for world populations of different geographical ancestry. Data investigating the impact of UGT polymorphisms on in vivo PK parameters are limited and summarised in Table 9 for the probe substrates included in this study. Overall, the limited data show that such an impact still needs to be fully characterised for endogenous substrates and xenobiotics in world populations.

**Table 9:** Impact of UGT isoform polymorphisms on pharmacokinetic markers of chronic exposure

Polymorphism	(Predominant) ethnicity/geographical ancestry	Substrate	Dose	Sample size	AUC ratio to wild type (%)			Comments	References
					wt/wt	wt/var	var/var		
UGT1A1*28	Caucasian	SN38	350 mg/m^2^	30/25/6	100	***136***	***161***		Innocenti et al. (2004)
UGT1A1*28	Japanese	SN38	100 mg/m^2^	10/7/0	100	337			Fukuda et al. (2018)
UGT1A1*28	USA	SN38	125 mg/m^2^	5/8/2	100	176	147		Jaeckle et al. (2010)
UGT1A1*28	Japan	SN38	150 mg/m^2^	41/7/3	100	120	261		Satoh et al. (2011)
UGT1A1*28	Caucasian	SN38	300 mg/m^2^	9/7/4	100	***141***	***259***		Iyer et al. (2002)
UGT1A1*28	Korea	SN38	80 mg/m^2^	69/12/0	100	88		Cisplatin was given as co-medication	Han et al. (2006)
UGT1A1*28	Japan	SN38	100 mg/m^2^	3/3/0	100	***401***			Hazama et al. (2010)
UGT1A1*28	Japan	SN38	50 mg/m^2^	7/1/1	100	219	172	Carboplatin was given as co-medication	Ando et al. (1998)
UGT1A1*28	Caucasian	SN38	600 mg	44/37/5	100	118	118		Paoluzzi et al. (2004)
UGT1A1*28	Italy	SN38	180 mg/m^2^	31/32/8	100	124	140	Patients on FOLFIRI regimen	Toffoli et al. (2006)
UGT1A1*28	USA	SN38	180 mg/m^2^	9/15/5	100	***105***	***209***	Patients on FOLFIRI regimen	Denlinger et al. (2009)
UGT1A1*28	USA	SN38	20 mg/m^2^	11/19/7	100	110	140		Stewart et al. (2007)
UGT1A1*28	Korean	SN38	65 or 80 mg/m^2^	93/14/0	100	85			Han et al. (2009)
UGT1A1*28	USA	SN38	50 mg/m^2^	14/7/0	100	91		Children	Bomgaars et al. (2007)
UGT1A1*28	Korean	Ezetimibe	10 mg	12/0/6	100		***177***		Bae et al. (2011)
UGT1A1*28	Japan	Telmisartan	80 mg	43/14/0	100	***53***			Yamada et al. (2011)
UGT1A1*28	Japan	Telmisartan	80 mg	16/3/4	100	***39***	***49***		Ieiri et al. (2011)
UGT1A1*28	Caucasian	Raltegravir	400 mg	27/0/30	100		141		Wenning et al. (2009)
UGT1A1*6	Japan	SN38	150 mg/m^2^	41/9/9	100	95	214		Satoh et al. (2011)
UGT1A1*6	Japanese	SN38	100 mg/m^2^	10/10/0	100	125			Fukuda et al. (2018)
UGT1A1*6	Korea	SN38	80 mg/m^2^	49/26/6	100	***111***	***176***	Cisplatin was given as co-medication	Han et al. (2006)
UGT1A1*6	Korean	Ezetimibe	10 mg	12/0/4	100		97		Bae et al. (2011)
UGT1A1*6	Japanese	Telmisartan	40 mg	10/2/0	100	114		Renal transplant patients	Miura et al. (2009)
UGT1A1*6	Japan	Telmisartan	80 mg	31/13/2	100	118	153		Yamada et al. (2011)
UGT1A1*6	Japan	Telmisartan	80 mg	16/7/1	100	109	193		Ieiri et al. (2011)
UGT1A3*2a	Japan	Telmisartan	80 mg	17/8/0	100	**57**			Ieiri et al. (2011)
UGT1A6*2	Thailand	Deferiprone	25 mg/kg	10/8/4	100	72	90		Limenta et al. (2008)
UGT1A9*22	Korea	SN38	80 mg/m^2^	11/45/23	100	***83***	***70***	Cisplatin was given as co-medication	Han et al. (2006)
UGT1A9*22	China	Mycophenolic acid	1–2 g	13/21/12	100	106	128	Renal transplant patients, co-medication cyclosporin and prednisolone	Zhang et al. (2008)
UGT2B7*2	Japanese	Telmisartan	40 mg	6/6/0	100	103		Renal transplant patients	Miura et al. (2009)
UGT2B7*2	Japan	Telmisartan	80 mg	24/28/5	100	110	149		Yamada et al. (2011)
UGT2B15*2	USA	Oxazepam	15 mg	6/20/4	100	***147***	***243***		He et al. (2009)

The associated polymorphism, the predominant ethnicity (or, if not given, the country of the study), the substrate, dose, sample size and ratios of the AUC is given, relative to wild type. For the sample size, numbers of wt/wt, wt/var and var/var are given. Ratios that are significantly different from wild type according to the cited study are shown in bold and italic

*Wt* wild type, *var* variant, *FOLFIRI* folinic acid, fluorouracil, irinotecan

Understanding the functional role of UGT SNPs is a key aspect to quantify the relationship between their frequency distributions (Fig. 6) and the pharmacokinetic consequences on UGT conjugation across world populations. Table 9 provides an account of such pharmacokinetic consequences; however, available studies from the literature are still limited. The consequences can be two-sided: an increased UGT activity would result in detoxification and a decreased UGT activity would result in an increase in the concentration of the toxic form (parent compound). Well-known exceptions to this rule include carboxylic acid-containing drugs that are metabolised by UGTs and form acyl glucuronides, like mycophenolic acid and telmisartan. These acyl glucuronides can cause idiosyncratic drug toxicity by binding covalently to proteins (Iwamura et al. 2017). For mycophenolic acid, indeed protein adducts have been found in vivo and these can result in several adverse effects (Shipkova et al. 2002).

#### UGT1A1

UGT1A1 in humans is one of the most important UGT isoforms in terms of glucuronidation and is known to have multiple clinically relevant polymorphisms that can contribute to variability in PK parameters (Mehboob et al. 2017; Miners et al. 2002). Polymorphisms in UGT1A1 are extensively studied and alteration in its activity can result in Gilbert’s syndrome, one of the most common syndromes in humans (Burchell and Hume 1999; Stingl et al. 2014). Gilbert’s syndrome results in hyperbilirubinaemia, as UGT1A1 is responsible for the metabolism of bilirubin. The frequency and type of polymorphisms differ between individuals from different geographical ancestry or ethnic backgrounds (Weber 1999) and this is also apparent from the frequencies of mutations in UGT1A1 that are responsible for Gilbert’s syndrome. A dinucleotide polymorphism in the TATA box promoter (UGT1A1*28) resulting in reduced UGT1A1 expression and Gilbert’s syndrome is detected in only 3% of Asians and ~ 15% in Europeans, while it can be up to 36% in Africans (Beutler et al. 1998). When looking at the frequency distribution of this SNP, clear interindividual differences are indeed detected across populations of different geographical ancestry (Fig. 6a, for references see Supplementary Material 4). As expected, Asian populations (especially East and Southeast Asians), as well as Oceanians, more frequently express the wild-type genotype, whereas other ethnicities predominantly express the heterozygous genotype. In Europe and Middle East, mixed frequencies in wild-type and heterozygous genotypes are observed. Another SNP in this isoform, UGT1A1*6, results in an amino acid substitution at position 71 (G71R). This mutation also causes Gilbert’s syndrome and is more frequently detected in Asians (Burchell and Hume 1999)*.*

As mentioned earlier, data gaps for pharmacokinetics of UGT1A1 probe substrates have been identified especially for Africans and Central Americans. Distribution of genotypes for UGT1A1*28 differs for these populations compared to Europeans and this highlights that PK data in phenotyped individuals from different geographical ancestries are needed to characterise isoform-specific UGT variability and related UFs. Besides, the high variability observed for SN38 may also be rationalised by the fact that only patient data were available and included in the meta-analysis, which may bias the analysis. Moreover, internal concentrations of SN38 can be influenced by the UGT1A1*28 mutation and some PK studies included only patients with the wild-type genotype, which also results in a bias in the calculation of the variability and the UF (Ri et al. 2018).

It is striking that variability in PK for ethinylestradiol is low (35–42%; Table 3) when compared to variability in PK for raltegravir and SN38 (up to 72%, Table 3). One possible explanation is the inclusion of only females as it is used as a contraceptive, and this may reduce variability. Indeed, genetic sex differences are known to have an important effect on interindividual differences in UGT enzymes as well and this aspect is further elaborated further down in the discussion. Another possible rationale may be that the identified SNPs have a larger impact on raltegravir and SN38 metabolism than on ethinylestradiol metabolism, which could be due to differences in docking resulting in different affinities and kinetics. Unfortunately, no studies that investigated the effect of UGT1A1 polymorphisms directly on ethinylestradiol PK in vivo were available. However, both ethinylestradiol and SN38 show significantly lower in vitro rates of metabolism with UGT1A1*28 polymorphic human liver microsomes (Zhang et al. 2007).

For the UGT1A1*28 polymorphism, significantly higher values for the AUCs were reported for SN38 which corresponds with a decrease in glucuronidation capacity (Table 9). For the UGT1A1*6 polymorphism, impact on PK parameters is less clear with only one study showing a significant increase in AUC for SN38. No in vivo PK data were available for the other UGT1A1 probe substrates and the effect of either UGT1A1 polymorphism on their PK parameters.

#### UGT1A3

For UGT1A3 polymorphisms, the frequency distribution is rather uniform across populations of different geographical ancestry. However, data were only available for three populations (North America, East-Asia and Europe) and the heterozygous genotype was the most represented one in all three populations (Fig. 6b). A contrasting exception was the observation of slightly higher frequencies for the wild type in East Asians compared to the other two populations.

UGT1A3 polymorphisms are associated with an increase in glucuronidation rates for a range of compounds. UGT1A3*2 (nucleotide changes T31C, G81A and T140C) polymorphism is correlated with an increase in glucuronidation of atorvastatin (Cho et al. 2012). Moreover, polymorphisms in UGT1A3 have been associated with polymorphisms in UGT1A1, which is due to a linkage disequilibrium within the UGT1A locus (Cho et al. 2012; Riedmaier et al. 2010; Saeki et al. 2006).

A study on telmisartan PK reported a significant influence of the *2a and *4a variants of the UGT1A3 polymorphisms, associated with a decrease and an increase in AUC, respectively (Ieiri et al. 2011, Table 9). Furthermore, a number of studies showed an impact of UGT1A1 and UGT2B7 polymorphisms on PK parameters of telmisartan, indicating that multiple UGT isoforms may be responsible for its glucuronidation and that multiple polymorphisms can, therefore, influence its PK parameters (Ieiri et al. 2011; Miura et al. 2009; Yamada et al. 2011).

#### UGT1A4

For UGT1A4, the *2 and *3 mutations are the two most common SNPs. UGT1A4*2 is a mutation at codon 24, resulting in an amino acid change from proline to threonine (P24T) because of a C70A SNP. UGT1A4*3 is a T142G SNP, resulting in an amino acid change at codon 48, from a leucine to a valine (L48V). In the frequency distribution data, no differences in C70A and T142G genotypes between populations from different geographical ancestries were observed (Fig. 6c/d). Compared to the mutation and the heterozygous genotype, the wild-type genotype is predominantly detected (C70A: > 80%, T142G: > 55%).

Studies on these SNPs show contradictory results. Neither polymorphism is significantly associated with trifluoperazine glucuronidation activity in vitro (Benoit-Biancamano et al. 2009b). However, decreased activities have been reported for benzidine, β-naphthylamine, steroids and tigogenin, but increased glucuronidation has been reported for clozapine and olanzapine with UGT1A4*3 (Ehmer et al. 2004; Ghotbi et al. 2010; Mori et al. 2005). This suggests that the impact of UGT1A4 mutations on PK parameters is substrate dependent, but the associated mechanism remains to be elucidated. The UGT1A4*3 has also been associated with decreased serum levels of lamotrigine which correspond to an increase in glucuronidation rates (Gulcebi et al. 2011; Reimers et al. 2016). For UGT1A4, no studies were encountered that studied effects of polymorphisms in this UGT isoform on in vivo PK parameters of the probe substrates.

#### UGT1A6

For UGT1A6, the most prominent mutation is UGT1A6*2, which is the result of two substitutions: T181A and R184S (Ciotti et al. 1997). The linkage disequilibrium between these polymorphisms is very high, as they are only 11 nucleotides apart (nucleotides 541 and 552) (McGreavey et al. 2005). A linkage disequilibrium between UGT1A6*2 and UGT1A1*28 is also observed (Lampe et al. 1999). No differences are seen across world populations in the frequency distribution of this polymorphism (Fig. 6e).

No impact on deferiprone PK was found in vivo for UGT1A6*2 (Limenta et al. 2008). However, an in vitro study showed that the UGT1A6*2 variant could lead to either a decrease or an increase in glucuronidation capacity for various phenolic compounds (Ciotti et al. 1997; Nagar et al. 2004). Lampe et al. (1999) showed that genetic sex had more influence on the PK parameters of deferiprone whereas polymorphism had no impact. This may be due to both the variation in UGT1A6 content and activity between males and females. Indeed, glucuronidation capacity has been shown to be higher in males with a 50% higher UGT1A6 protein content in males compared to that in females (Bock et al. 1994; Court et al. 2001).

#### UGT1A9

For UGT1A9, SNPs have been associated with a range of impacts on the PK of xenobiotics. T98C (UGT1A9*3) may result in a decrease in glucuronidation activity, although the reported results are contradictory (Girard et al. 2004; Jiao et al. 2008; Villeneuve et al. 2003). The T-275A SNP, which is located in the promotor region, is associated with an increase in glucuronidation rates, while in another study, the glucuronidation rate of mycophenolic acid remained unaffected (Girard et al. 2004; Jiao et al. 2008; Kuypers et al. 2005; Mazidi et al. 2013). Multiple linkage disequilibria are known for polymorphisms in UGT1A9 since SNPs in UGT1A9 are linked to SNPs in UGT1A7 and UGT1A6 (Saeki et al. 2006). The frequency distributions of these genotypes across several populations are described in Supplementary Material 5.

The SNP with the most apparent differences in frequencies between populations is a ‘T’ deleted at position-118 in the promotor region of the gene, UGT1A9*22 (Cecchin et al. 2009). Japanese individuals show a different distribution compared to that in other populations including other Asian populations (Fig. 6f). In other world populations, the heterozygous genotype is the most occurring, while in Japan most prominent frequencies are a mix between the heterozygous genotype and the homozygous mutation. It is shown that combinations of haplotypes differ between Caucasians and Asians and this might explain the large differences in frequencies observed here (Saeki et al. 2006).

The effect of UGT1A9*22 on PK parameters remains unclear since an increased transcriptional activity has been reported, but it was not associated with an impact on mycophenolic acid PK parameters (Jiao et al. 2008; Yamanaka et al. 2004; Zhang et al. 2008). A significant decrease is demonstrated in AUC for SN38 with this mutation, although SN38 is mainly metabolised by UGT1A1 (Han et al. 2006).

#### UGT2B7

For UGT2B7, the frequencies of the C802T mutation are quite comparable for the three world regions (Europe, North America, South America, Fig. 6g) represented in the PK database and indeed, not much variability is observed in the PK parameters of zidovudine. The SNP C802T in UGT2B7 results in an amino acid substitution at residue 268, from histidine to tyrosine (H268Y, UGT2B7*2) at the N-terminal substrate binding site of the enzyme (Yuan et al. 2015). It is demonstrated that this variant form has the same localisation as the wild type. Moreover, it is demonstrated that UGT2B7*2 can form both homodimers and heterodimers with wild-type and other polymorphic enzymes, albeit with a decrease in affinity (Yuan et al. 2015). Coffman et al. (1998) showed that the 268Y form of the UGT was ten times more efficient in the glucuronidation of buprenorphine than the 268H form. However, no differences were detected for some other opioids, like morphine and codeine. In another study of Coffman et al. (2003), it was demonstrated that opioids bind to amino acids 84–118 of the UGT, which implies that mutations at other places are less likely to influence the binding of opioids to UGT. However, also polymorphisms outside the substrate-binding pocket can still influence the dynamics of substrate binding by, for example, altering the packing of the enzyme and thereby influencing the active site (Rutherford et al. 2008).

It is demonstrated that UGT2B7*2 in a hetero-dimer with the wild-type enzyme has an impaired glucuronidation activity for zidovudine (Yuan et al. 2015). For other chemicals including valproic acid, tamoxifen, and lamotrigine, UGT2B7 polymorphism has been shown to affect plasma concentrations (Blevins-Primeau et al. 2009; Du et al. 2016; Petrenaite et al. 2018; Sun et al. 2015; Wang et al. 2018b). Molecular docking would provide an insight into the binding of substrates to UGT2B7 and other UGTs and the effect of polymorphisms hereon. However, a complete crystal structure is not available yet for human UGTs (Dong et al. 2012). The partial crystal structure of UGT2B7 that is available does not include the N-terminal substrate-binding domain and consequently does not provide insight into substrate binding (Miley et al. 2007). No in vivo data exploring the relationship between UGT2B7 polymorphisms and PK parameters of zidovudine or codeine were available. Only two studies investigated the impact of UGT2B7*2 on telmisartan PK in Japanese adults. In both studies, no significant differences in AUC were found (Miura et al. 2009; Yamada et al. 2011).

#### UGT2B15

For UGT2B15, the most common polymorphism is known as UGT2B15*2 and this mutation results in the substitution of an aspartic acid with a tyrosine at position 85 (D85Y). The frequency distribution of this polymorphism is comparable for different populations (Fig. 6h). In one study, different ethnicities (African-American, Hispanic-American, Chinese-American, Japanese-American and Caucasian-American) in North-America were compared and all different ethnicities showed approximately the same distribution, with the heterozygous genotype being the predominant genotype (Riedy et al. 2000).

For this polymorphism, no differences were found in relation to the metabolic and PK profile of tamoxifen (Romero-Lorca et al. 2015; Sutiman et al. 2016). However, acetaminophen total clearance was significantly influenced by this polymorphism (Court et al. 2017). Moreover, in vitro data show lower median activities for S-oxazepam glucuronidation with microsomes containing the UGT2B15*2 polymorphism and a lower intrinsic clearance of bisphenol A with this SNP (Court et al. 2004; Hanioka et al. 2011). Finally, lower systemic clearance of lorazepam is reported in Asian individuals homozygous for UGT2B15*2 and the authors suggested that this polymorphism is a major contributor to interindividual differences in lorazepam PK (Chung et al. 2005). A significant increase in AUC has been observed for UGT2B15*2 for individuals with at least one polymorphic gene. According to the study of He et al. (2009), the polymorphism accounts for 34% of the interindividual differences in oxazepam oral clearance (Table 9).

## Conclusions and future perspectives

This manuscript aimed to quantify interindividual differences in UGT isoform-specific metabolism for probe substrates. Hierarchical Bayesian meta-analyses for pharmacokinetic markers of acute (Cmax) and chronic exposure (AUC/clearance) were performed for a total of 14 probe substrates of the seven clinically most relevant UGT isoforms (UGT1A1, UGT1A3, UGT1A4, UGT1A6, UGT1A9, UGT2B7 and UGT2B15). The resulting variability distributions and the UGT-related UFs showed that the default factor of 3.16 would not be exceeded for at least 97.5% of non-phenotyped healthy adults when considering the median value, with a few exceptions (1-OH-midazolam, ezetimibe, raltegravir, SN38 and trifluoperazine).

Overall, interindividual differences in kinetics for intravenous- and oral routes of administration were comparable. A possible explanation for such similarities lies in the fact that UGTs are more abundant in the liver compared to the intestine, so that the impact of first-pass metabolism for the included probe substrates is low (Lv et al. 2019). In contrast, similar analysis performed for CYP3A4 probe substrates revealed larger interindividual differences for markers of oral chronic exposure compared to their IV counterparts (Darney et al. 2019). Several UGT isoforms are also expressed in the kidney, including UGT1A6, UGT1A9 and UGT2B7 (Ohno and Nakajin 2009). This would have no influence on the first-pass metabolism, but variability estimates are likely to reflect hepatic and renal UGT metabolism for the probe substrates metabolised by these isoforms.

Overall, data gaps have been identified from this human UGT PK database for a range of non-phenotyped and phentoyped populations of different geographical ancestries as well as sensitive subgroups of the population, including neonates, children and the elderly. A typical example is the lack of PK data for the African population which shows broad genetic diversity in the frequency of UGT polymorphisms. Such PK data are needed to integrate genotype frequencies in different populations and to generate distributions to address interphenotypic differences which then allow the derivation of UGT-related UFs as well as chemical-specific adjustment factors (Campbell and Tishkoff 2008; Gaibar et al. 2018; Novillo et al. 2018).

Indeed, different UGT polymorphisms can have (substrate-dependent) impact on interphenotypic differences in PK parameters, particularly for the UGT1A1 isoform while new polymorphisms are still being characterised (Liu et al. 2019). In this light, it is recommended to investigate interphenotypic differences in relation to UGT polymorphisms rather than geographical ancestry since polymorphisms are better predictors of altered PK compared to ethnicity alone (Darney et al. 2019; Wu et al. 2018).

Although isoform-specific variability was investigated here using specific probe substrates, most often several UGT isoforms are involved in the glucuronidation of xenobiotics in a concentration-dependent manner. For example, acetaminophen glucuronidation by human liver microsomes can be mediated by multiple UGTs. Three isoforms are most active, UGT1A1 is the main contributor at toxic concentrations and UGT1A6 is the most active at low concentrations (Court et al. 2001). Besides the contribution of several isoforms to the glucuronidation of one compound, other factors could also contribute to interindividual differences in metabolism by UGTs. For example, correlations have been established between UGT abundances and their activity, and variability in glucuronidation is comparable to variability in UGT protein abundance (Achour et al. 2017). In addition to interphenotypic differences, age differences have been described to impact UGT expression and activities, particularly in neonates and young infants, leading to slower kinetics and elimination through a reduction of PK parameters by several folds compared to that in healthy adults (Bhatt et al. 2019; Court 2010; Dorne et al. 2001b).

UGTs are also involved in the metabolism of large numbers of xenobiotics, other than pharmaceuticals, like environmental contaminants and naturally occurring compounds. However, for these compounds multiple UGT isoforms are often involved in their conjugation. For example, isoflavones are conjugated by multiple UGT isoforms in human liver microsomes (Tang et al. 2009). Besides the involvement of several UGT isoforms in conjugation, human kinetic data for most environmental contaminants and food-relevant chemicals are still scarce in the literature.

Taken all together, investigation of isoform-specific UGT-related age and interphenotypic differences in world populations will allow the characterisation and publication of full variability distributions for human populations in an open source format (as illustrated here with the relatively limited data available). For food-relevant compounds, it is foreseen that such distributions can then be combined with in vitro data characterising the kinetics of UGT isoform-specific metabolism for a whole host of compounds including flavourings, food additives, pesticides, mycotoxins and other contaminants to develop quantitative in vitro–in vivo extrapolation (QIVIVE) models. It is important to note that the isoform-specific distributions and uncertainty factors generated in this study have been drawn from pharmaceutical data and can be applied to a large number of UGT substrates with short half-lives. As variability between the UGT isoforms has been shown to be relatively similar, chemical-specific variability can also be derived, even for compounds conjugated by multiple UGT isoforms. A major data gap is the lack of human in vivo PK data and mechanistically validated in vitro assays in human intestinal, liver, and kidney cells. Further research and validation efforts in these areas would allow to characterise either direct isoform-specific UGT metabolism, cytochrome P450 and/or influx or efflux transport with subsequent UGT conjugation as well as differential renal or bile excretion to further develop such QIVIVE models and gain experience and confidence in their use in daily chemical risk assessment.

## Electronic supplementary material

Below is the link to the electronic supplementary material.

Supplementary file1 (DOCX 30 kb)

Supplementary file2 (DOCX 1169 kb)

Supplementary file3 (DOCX 19508 kb)

Supplementary file4 (DOCX 42 kb)

Supplementary file5 (DOCX 4418 kb)
