# Supplementary material for: Human variability in isoform-specific UDP-glucuronosyltransferases: markers of acute and chronic exposure, polymorphisms and uncertainty factors
Source: Arch Toxicol. 2020 May 15;94(8):2637–61. doi: 10.1007/s00204-020-02765-8 (PMC7395075; doi:10.1007/s00204-020-02765-8)
Supplement: Supplementary file 1 — Supplementary file1 (DOCX 30 kb) [file 204_2020_2765_MOESM1_ESM.docx]

**Supplementary Material 1 –** Search queries for the different compounds for Scopus and PubMed and selection criteria

***1-OH midazolam***

*Scopus*

( TITLE-ABS ( “1-hydroxymidazolam” ) OR TITLE-ABS ( “1’-Hydroxymidazolam” ) OR TITLE-ABS ( “alpha-hydroxymidazolam” ) ) AND ( TITLE-ABS ( patient* ) OR TITLE-ABS ( human ) OR TITLE-ABS ( adult ) OR TITLE-ABS ( adults ) OR TITLE-ABS ( child ) OR TITLE-ABS ( children ) OR TITLE-ABS ( infant ) OR TITLE-ABS ( neonate ) OR TITLE-ABS ( newborn ) OR TITLE-ABS ( newborns ) OR TITLE-ABS ( elderly ) OR TITLE-ABS ( "pregnant women" ) OR TITLE-ABS ( men ) OR TITLE-ABS ( women ) OR TITLE-ABS ( "ethnic group" ) OR TITLE-ABS ( caucasian ) OR TITLE-ABS ( asian ) OR TITLE-ABS ( african ) OR TITLE-ABS ( "genetic polymorphism*" ) OR TITLE-ABS ( "individual susceptibility" ) OR TITLE-ABS ( "gene environment" ) OR TITLE-ABS ( "ethnic variability" ) OR TITLE-ABS ( "Afro American" ) OR TITLE-ABS ( hispanic ) OR TITLE-ABS ( "race difference" ) OR TITLE-ABS ( "age difference" ) OR TITLE-ABS ( "race differences" ) OR TITLE-ABS ( "age differences" ) OR TITLE-ABS ( "gender differences" ) OR TITLE-ABS ( "gender difference" ) OR TITLE-ABS ( "sex difference" ) OR TITLE-ABS ( "sex differences" ) ) AND ( TITLE-ABS-KEY ( auc ) OR TITLE-ABS-KEY ( area AND under AND the AND curve ) OR TITLE-ABS-KEY ( area AND under AND curve ) OR TITLE-ABS-KEY ( half AND life ) OR TITLE-ABS-KEY ( half-life ) OR TITLE-ABS-KEY ( half-lives ) OR TITLE-ABS-KEY ( clearance ) OR TITLE-ABS-KEY ( cmax ) OR TITLE-ABS-KEY ( pharmacokinetic ) OR TITLE-ABS-KEY ( pharmacokinetics ) OR TITLE-ABS-KEY ( toxicokinetic ) OR TITLE-ABS-KEY ( toxicokinetics ) ) AND NOT ( TITLE-ABS-KEY ( "cell line*" ) OR TITLE-ABS-KEY ( "cell culture*" ) OR TITLE-ABS-KEY ( rat ) OR TITLE-ABS-KEY ( rats ) OR TITLE-ABS-KEY ( mouse ) OR TITLE-ABS-KEY ( mice ) ) AND ( EXCLUDE ( DOCTYPE,"re" ) ) AND ( LIMIT-TO ( LANGUAGE,"English" ) )

*PubMed*

(((("1'-hydroxymidazolam"[Title/Abstract] OR alpha-hydroxymidazolam[Title/Abstract] OR "1-hydroxymidazolam"[Title/Abstract])) AND (auc[Title/Abstract] OR "area under the curve"[Title/Abstract] OR "area under curve"[Title/Abstract] OR "half life"[Title/Abstract] OR half-life[Title/Abstract] OR half-lives[Title/Abstract] OR clearance[Title/Abstract] OR cmax[Title/Abstract] OR pharmacokinetic[Title/Abstract] OR pharmacokinetics[Title/Abstract] OR toxicokinetic[Title/Abstract] OR toxicokinetics)) AND (patient[Title/Abstract] OR patients[Title/Abstract] OR human[Title/Abstract] OR (adult[Title/Abstract]) OR adults[Title/Abstract] OR child[Title/Abstract] OR children[Title/Abstract] OR infant[Title/Abstract] OR neonate[Title/Abstract] OR newborn[Title/Abstract] OR newborns[Title/Abstract] OR elderly[Title/Abstract] OR "pregnant women"[Title/Abstract] OR men[Title/Abstract] OR women[Title/Abstract] OR "ethnic group"[Title/Abstract] OR caucasian[Title/Abstract] OR asian[Title/Abstract] OR african[Title/Abstract] OR "genetic polymorphism*"[Title/Abstract] OR "individual susceptibility"[Title/Abstract] OR "gene environment"[Title/Abstract] OR "ethnic variability"[Title/Abstract] OR "Afro American"[Title/Abstract] OR hispanic[Title/Abstract] OR "race difference"[Title/Abstract] OR "age difference"[Title/Abstract] OR "race differences"[Title/Abstract] OR "age differences"[Title/Abstract] OR "gender differences"[Title/Abstract] OR "gender difference"[Title/Abstract] OR "sex difference"[Title/Abstract] OR "sex differences"[Title/Abstract])) NOT ("cell line*"[Title/Abstract] OR "cell culture*"[Title/Abstract] OR rat[Title/Abstract] OR rats[Title/Abstract] OR mouse[Title/Abstract] OR mice[Title/Abstract])

***Codeine***

*Scopus*

( ( TITLE-ABS ( codeine ) ) ) AND ( TITLE-ABS ( volunteer* ) OR TITLE-ABS ( human ) OR TITLE-ABS ( adult ) OR TITLE-ABS ( adults ) OR TITLE-ABS ( men ) OR TITLE-ABS ( women ) OR TITLE-ABS ( "ethnic group" ) OR TITLE-ABS ( caucasian ) OR TITLE-ABS ( asian ) OR TITLE-ABS ( african ) OR TITLE-ABS ( "genetic polymorphism*" ) OR TITLE-ABS ( "individual susceptibility" ) OR TITLE-ABS ( "gene environment" ) OR TITLE-ABS ( "ethnic variability" ) OR TITLE-ABS ( "Afro American" ) OR TITLE-ABS ( hispanic ) OR TITLE-ABS ( "race difference" ) OR TITLE-ABS ( "age difference" ) OR TITLE-ABS ( "race differences" ) OR TITLE-ABS ( "age differences" ) OR TITLE-ABS ( "gender differences" ) OR TITLE-ABS ( "gender difference" ) OR TITLE-ABS ( "sex difference" ) OR TITLE-ABS ( "sex differences" ) ) AND ( TITLE-ABS-KEY ( auc ) OR TITLE-ABS-KEY ( area AND under AND the AND curve ) OR TITLE-ABS-KEY ( area AND under AND curve ) OR TITLE-ABS-KEY ( half AND life ) OR TITLE-ABS-KEY ( half-life ) OR TITLE-ABS-KEY ( half-lives ) OR TITLE-ABS-KEY ( clearance ) OR TITLE-ABS-KEY ( cmax ) OR TITLE-ABS-KEY ( pharmacokinetic ) OR TITLE-ABS-KEY ( pharmacokinetics ) OR TITLE-ABS-KEY ( toxicokinetic ) OR TITLE-ABS-KEY ( toxicokinetics ) ) AND NOT ( TITLE-ABS-KEY ( "cell line*" ) OR TITLE-ABS-KEY ( "cell culture*" ) OR TITLE-ABS-KEY ( rat ) OR TITLE-ABS-KEY ( rats ) OR TITLE-ABS-KEY ( mouse ) OR TITLE-ABS-KEY ( mice ) ) AND ( EXCLUDE ( DOCTYPE , "re" ) ) AND ( LIMIT-TO ( LANGUAGE , "English" ) )

*PubMed*

((((codeine[Title/Abstract])) AND (auc[Title/Abstract] OR "area under the curve"[Title/Abstract] OR "area under curve"[Title/Abstract] OR "half life"[Title/Abstract] OR half-life[Title/Abstract] OR half-lives[Title/Abstract] OR clearance[Title/Abstract] OR cmax[Title/Abstract] OR pharmacokinetic[Title/Abstract] OR pharmacokinetics[Title/Abstract] OR toxicokinetic[Title/Abstract] OR toxicokinetics)) AND (volunteer[Title/Abstract] OR volunteers[Title/Abstract] OR human[Title/Abstract] OR (adult[Title/Abstract]) OR adults[Title/Abstract] OR men[Title/Abstract] OR women[Title/Abstract] OR "ethnic group"[Title/Abstract] OR caucasian[Title/Abstract] OR asian[Title/Abstract] OR african[Title/Abstract] OR "genetic polymorphism*"[Title/Abstract] OR "individual susceptibility"[Title/Abstract] OR "gene environment"[Title/Abstract] OR "ethnic variability"[Title/Abstract] OR "Afro American"[Title/Abstract] OR hispanic[Title/Abstract] OR "race difference"[Title/Abstract] OR "age difference"[Title/Abstract] OR "race differences"[Title/Abstract] OR "age differences"[Title/Abstract] OR "gender differences"[Title/Abstract] OR "gender difference"[Title/Abstract] OR "sex difference"[Title/Abstract] OR "sex differences"[Title/Abstract])) NOT ("cell line*"[Title/Abstract] OR "cell culture*"[Title/Abstract] OR rat[Title/Abstract] OR rats[Title/Abstract] OR mouse[Title/Abstract] OR mice[Title/Abstract])

***Deferiprone***

*Scopus*

( ( TITLE-ABS ( deferiprone ) OR TITLE-ABS ( ferriprox ) ) ) AND ( TITLE-ABS ( patient* ) OR TITLE-ABS ( human ) OR TITLE-ABS ( adult ) OR TITLE-ABS ( adults ) OR TITLE-ABS ( child ) OR TITLE-ABS ( children ) OR TITLE-ABS ( infant ) OR TITLE-ABS ( neonate ) OR TITLE-ABS ( newborn ) OR TITLE-ABS ( newborns ) OR TITLE-ABS ( elderly ) OR TITLE-ABS ( "pregnant women" ) OR TITLE-ABS ( men ) OR TITLE-ABS ( women ) OR TITLE-ABS ( "ethnic group" ) OR TITLE-ABS ( caucasian ) OR TITLE-ABS ( asian ) OR TITLE-ABS ( african ) OR TITLE-ABS ( "genetic polymorphism*" ) OR TITLE-ABS ( "individual susceptibility" ) OR TITLE-ABS ( "gene environment" ) OR TITLE-ABS ( "ethnic variability" ) OR TITLE-ABS ( "Afro American" ) OR TITLE-ABS ( hispanic ) OR TITLE-ABS ( "race difference" ) OR TITLE-ABS ( "age difference" ) OR TITLE-ABS ( "race differences" ) OR TITLE-ABS ( "age differences" ) OR TITLE-ABS ( "gender differences" ) OR TITLE-ABS ( "gender difference" ) OR TITLE-ABS ( "sex difference" ) OR TITLE-ABS ( "sex differences" ) ) AND ( TITLE-ABS-KEY ( auc ) OR TITLE-ABS-KEY ( area AND under AND the AND curve ) OR TITLE-ABS-KEY ( area AND under AND curve ) OR TITLE-ABS-KEY ( half AND life ) OR TITLE-ABS-KEY ( half-life ) OR TITLE-ABS-KEY ( half-lives ) OR TITLE-ABS-KEY ( clearance ) OR TITLE-ABS-KEY ( cmax ) OR TITLE-ABS-KEY ( vmax ) OR TITLE-ABS-KEY ( km ) OR TITLE-ABS-KEY ( "michaelis constant" ) OR TITLE-ABS-KEY ( pharmacokinetic ) OR TITLE-ABS-KEY ( pharmacokinetics ) OR TITLE-ABS-KEY ( toxicokinetic ) OR TITLE-ABS-KEY ( toxicokinetics ) ) AND NOT ( TITLE-ABS-KEY ( "cell line*" ) OR TITLE-ABS-KEY ( "cell culture*" ) OR TITLE-ABS-KEY ( rat ) OR TITLE-ABS-KEY(rats) OR TITLE-ABS-KEY ( mouse ) OR TITLE-ABS-KEY ( mice ) ) AND ( EXCLUDE ( DOCTYPE,"re" ) ) AND ( LIMIT-TO ( LANGUAGE,"English" ) )

*PubMed*

((((deferiprone[Title/Abstract] OR ferriprox[Title/Abstract])) AND (auc[Title/Abstract] OR "area under the curve"[Title/Abstract] OR "area under curve"[Title/Abstract] OR "half life"[Title/Abstract] OR half-life[Title/Abstract] OR half-lives[Title/Abstract] OR clearance[Title/Abstract] OR cmax[Title/Abstract] OR vmax[Title/Abstract] OR km[Title/Abstract] OR "michaelis constant"[Title/Abstract] OR pharmacokinetic[Title/Abstract] OR pharmacokinetics[Title/Abstract] OR toxicokinetic[Title/Abstract] OR toxicokinetics)) AND (patient[Title/Abstract] OR patients[Title/Abstract] OR human[Title/Abstract] OR (adult[Title/Abstract]) OR adults[Title/Abstract] OR child[Title/Abstract] OR children[Title/Abstract] OR infant[Title/Abstract] OR neonate[Title/Abstract] OR newborn[Title/Abstract] OR newborns[Title/Abstract] OR elderly[Title/Abstract] OR "pregnant women"[Title/Abstract] OR men[Title/Abstract] OR women[Title/Abstract] OR "ethnic group"[Title/Abstract] OR caucasian[Title/Abstract] OR asian[Title/Abstract] OR african[Title/Abstract] OR "genetic polymorphism*"[Title/Abstract] OR "individual susceptibility"[Title/Abstract] OR "gene environment"[Title/Abstract] OR "ethnic variability"[Title/Abstract] OR "Afro American"[Title/Abstract] OR hispanic[Title/Abstract] OR "race difference"[Title/Abstract] OR "age difference"[Title/Abstract] OR "race differences"[Title/Abstract] OR "age differences"[Title/Abstract] OR "gender differences"[Title/Abstract] OR "gender difference"[Title/Abstract] OR "sex difference"[Title/Abstract] OR "sex differences"[Title/Abstract])) NOT ("cell line*"[Title/Abstract] OR "cell culture*"[Title/Abstract] OR rat[Title/Abstract] OR rats[Title/Abstract] OR mouse[Title/Abstract] OR mice[Title/Abstract])

***Entacapone***

*Scopus*

( TITLE-ABS ( entacapone ) OR TITLE-ABS ( comtan ) OR TITLE-ABS ( comtess ) ) AND ( TITLE-ABS ( patient* ) OR TITLE-ABS ( human ) OR TITLE-ABS ( adult ) OR TITLE-ABS ( adults ) OR TITLE-ABS ( child ) OR TITLE-ABS ( children ) OR TITLE-ABS ( infant ) OR TITLE-ABS ( neonate ) OR TITLE-ABS ( newborn ) OR TITLE-ABS ( newborns ) OR TITLE-ABS ( elderly ) OR TITLE-ABS ( "pregnant women" ) OR TITLE-ABS ( men ) OR TITLE-ABS ( women ) OR TITLE-ABS ( "ethnic group" ) OR TITLE-ABS ( caucasian ) OR TITLE-ABS ( asian ) OR TITLE-ABS ( african ) OR TITLE-ABS ( "genetic polymorphism*" ) OR TITLE-ABS ( "individual susceptibility" ) OR TITLE-ABS ( "gene environment" ) OR TITLE-ABS ( "ethnic variability" ) OR TITLE-ABS ( "Afro American" ) OR TITLE-ABS ( hispanic ) OR TITLE-ABS ( "race difference" ) OR TITLE-ABS ( "age difference" ) OR TITLE-ABS ( "race differences" ) OR TITLE-ABS ( "age differences" ) OR TITLE-ABS ( "gender differences" ) OR TITLE-ABS ( "gender difference" ) OR TITLE-ABS ( "sex difference" ) OR TITLE-ABS ( "sex differences" ) ) AND ( TITLE-ABS-KEY ( auc ) OR TITLE-ABS-KEY ( area AND under AND the AND curve ) OR TITLE-ABS-KEY ( area AND under AND curve ) OR TITLE-ABS-KEY ( half AND life ) OR TITLE-ABS-KEY ( half-life ) OR TITLE-ABS-KEY ( half-lives ) OR TITLE-ABS-KEY ( clearance ) OR TITLE-ABS-KEY ( cmax ) OR TITLE-ABS-KEY ( vmax ) OR TITLE-ABS-KEY ( km ) OR TITLE-ABS-KEY ( "michaelis constant" ) OR TITLE-ABS-KEY ( pharmacokinetic ) OR TITLE-ABS-KEY ( pharmacokinetics ) OR TITLE-ABS-KEY ( toxicokinetic ) OR TITLE-ABS-KEY ( toxicokinetics ) ) AND NOT ( TITLE-ABS-KEY ( "cell line*" ) OR TITLE-ABS-KEY ( "cell culture*" ) OR TITLE-ABS-KEY ( rat ) OR TITLE-ABS-KEY ( rats ) OR TITLE-ABS-KEY ( mouse ) OR TITLE-ABS-KEY ( mice ) ) AND ( EXCLUDE ( DOCTYPE,"re" ) ) AND ( LIMIT-TO ( LANGUAGE,"English" ) )

*PubMed*

((((entacapone[Title/Abstract] OR comtan[Title/Abstract] OR comtess[Title/Abstract])) AND (auc[Title/Abstract] OR "area under the curve"[Title/Abstract] OR "area under curve"[Title/Abstract] OR "half life"[Title/Abstract] OR half-life[Title/Abstract] OR half-lives[Title/Abstract] OR clearance[Title/Abstract] OR cmax[Title/Abstract] OR vmax[Title/Abstract] OR km[Title/Abstract] OR "michaelis constant"[Title/Abstract] OR pharmacokinetic[Title/Abstract] OR pharmacokinetics[Title/Abstract] OR toxicokinetic[Title/Abstract] OR toxicokinetics)) AND (patient[Title/Abstract] OR patients[Title/Abstract] OR human[Title/Abstract] OR (adult[Title/Abstract]) OR adults[Title/Abstract] OR child[Title/Abstract] OR children[Title/Abstract] OR infant[Title/Abstract] OR neonate[Title/Abstract] OR newborn[Title/Abstract] OR newborns[Title/Abstract] OR elderly[Title/Abstract] OR "pregnant women"[Title/Abstract] OR men[Title/Abstract] OR women[Title/Abstract] OR "ethnic group"[Title/Abstract] OR caucasian[Title/Abstract] OR asian[Title/Abstract] OR african[Title/Abstract] OR "genetic polymorphism*"[Title/Abstract] OR "individual susceptibility"[Title/Abstract] OR "gene environment"[Title/Abstract] OR "ethnic variability"[Title/Abstract] OR "Afro American"[Title/Abstract] OR hispanic[Title/Abstract] OR "race difference"[Title/Abstract] OR "age difference"[Title/Abstract] OR "race differences"[Title/Abstract] OR "age differences"[Title/Abstract] OR "gender differences"[Title/Abstract] OR "gender difference"[Title/Abstract] OR "sex difference"[Title/Abstract] OR "sex differences"[Title/Abstract])) NOT ("cell line*"[Title/Abstract] OR "cell culture*"[Title/Abstract] OR rat[Title/Abstract] OR rats[Title/Abstract] OR mouse[Title/Abstract] OR mice[Title/Abstract])

***Ethinylestradiol***

*Scopus*

( ( TITLE-ABS ( ethinylestradiol )  OR  TITLE-ABS ( "ethinyl estradiol" ) ) )  AND  ( TITLE-ABS ( patient* )  OR  TITLE-ABS ( human )  OR  TITLE-ABS ( adult )  OR  TITLE-ABS ( adults )  OR  TITLE-ABS ( child )  OR  TITLE-ABS ( children )  OR  TITLE-ABS ( infant )  OR  TITLE-ABS ( neonate )  OR  TITLE-ABS ( newborn )  OR  TITLE-ABS ( newborns )  OR  TITLE-ABS ( elderly )  OR  TITLE-ABS ( "pregnant women" )  OR  TITLE-ABS ( men )  OR  TITLE-ABS ( women )  OR  TITLE-ABS ( "ethnic group" )  OR  TITLE-ABS ( caucasian )  OR  TITLE-ABS ( asian )  OR  TITLE-ABS ( african )  OR  TITLE-ABS ( "genetic polymorphism*" )  OR  TITLE-ABS ( "individual susceptibility" )  OR  TITLE-ABS ( "gene environment" )  OR  TITLE-ABS ( "ethnic variability" )  OR  TITLE-ABS ( "Afro American" )  OR  TITLE-ABS ( hispanic )  OR  TITLE-ABS ( "race difference" )  OR  TITLE-ABS ( "age difference" )  OR  TITLE-ABS ( "race differences" )  OR  TITLE-ABS ( "age differences" )  OR  TITLE-ABS ( "gender differences" )  OR  TITLE-ABS ( "gender difference" )  OR  TITLE-ABS ( "sex difference" )  OR  TITLE-ABS ( "sex differences" ) )  AND  ( TITLE-ABS-KEY ( auc )  OR  TITLE-ABS-KEY ( area  AND  under  AND  the  AND  curve )  OR  TITLE-ABS-KEY ( area  AND  under  AND  curve )  OR  TITLE-ABS-KEY ( half  AND  life )  OR  TITLE-ABS-KEY ( half-life )  OR  TITLE-ABS-KEY ( half-lives )  OR  TITLE-ABS-KEY ( clearance )  OR  TITLE-ABS-KEY ( cmax )  OR  TITLE-ABS-KEY ( vmax )  OR  TITLE-ABS-KEY ( km )  OR  TITLE-ABS-KEY ( "michaelis constant" )  OR  TITLE-ABS-KEY ( pharmacokinetic )  OR  TITLE-ABS-KEY ( pharmacokinetics )  OR  TITLE-ABS-KEY ( toxicokinetic )  OR  TITLE-ABS-KEY ( toxicokinetics ) )  AND NOT  ( TITLE-ABS-KEY ( "cell line*" )  OR  TITLE-ABS-KEY ( "cell culture*" )  OR  TITLE-ABS-KEY ( rat )  OR  TITLE-ABS-KEY ( rats )  OR  TITLE-ABS-KEY ( mouse )  OR  TITLE-ABS-KEY ( mice ) )  AND  ( EXCLUDE ( DOCTYPE ,  "re" ) )  AND  ( LIMIT-TO ( LANGUAGE ,  "English" ) )

*PubMed*

((((ethinylestradiol[Title/Abstract] OR "ethinyl estradiol"[Title/Abstract])) AND (auc[Title/Abstract] OR "area under the curve"[Title/Abstract] OR "area under curve"[Title/Abstract] OR "half life"[Title/Abstract] OR half-life[Title/Abstract] OR half-lives[Title/Abstract] OR clearance[Title/Abstract] OR cmax[Title/Abstract] OR vmax[Title/Abstract] OR km[Title/Abstract] OR "michaelis constant"[Title/Abstract] OR pharmacokinetic[Title/Abstract] OR pharmacokinetics[Title/Abstract] OR toxicokinetic[Title/Abstract] OR toxicokinetics)) AND (patient[Title/Abstract] OR patients[Title/Abstract] OR human[Title/Abstract] OR (adult[Title/Abstract]) OR adults[Title/Abstract] OR child[Title/Abstract] OR children[Title/Abstract] OR infant[Title/Abstract] OR neonate[Title/Abstract] OR newborn[Title/Abstract] OR newborns[Title/Abstract] OR elderly[Title/Abstract] OR "pregnant women"[Title/Abstract] OR men[Title/Abstract] OR women[Title/Abstract] OR "ethnic group"[Title/Abstract] OR caucasian[Title/Abstract] OR asian[Title/Abstract] OR african[Title/Abstract] OR "genetic polymorphism*"[Title/Abstract] OR "individual susceptibility"[Title/Abstract] OR "gene environment"[Title/Abstract] OR "ethnic variability"[Title/Abstract] OR "Afro American"[Title/Abstract] OR hispanic[Title/Abstract] OR "race difference"[Title/Abstract] OR "age difference"[Title/Abstract] OR "race differences"[Title/Abstract] OR "age differences"[Title/Abstract] OR "gender differences"[Title/Abstract] OR "gender difference"[Title/Abstract] OR "sex difference"[Title/Abstract] OR "sex differences"[Title/Abstract])) NOT ("cell line*"[Title/Abstract] OR "cell culture*"[Title/Abstract] OR rat[Title/Abstract] OR rats[Title/Abstract] OR mouse[Title/Abstract] OR mice[Title/Abstract])

***Ezetimibe***

*Scopus*

( ( TITLE-ABS ( ezetimibe ) OR TITLE-ABS ( ezetrol ) OR TITLE-ABS ( zetia ) ) ) AND ( TITLE-ABS ( patient* ) OR TITLE-ABS ( human ) OR TITLE-ABS ( adult ) OR TITLE-ABS ( adults ) OR TITLE-ABS ( child ) OR TITLE-ABS ( children ) OR TITLE-ABS ( infant ) OR TITLE-ABS ( neonate ) OR TITLE-ABS ( newborn ) OR TITLE-ABS ( newborns ) OR TITLE-ABS ( elderly ) OR TITLE-ABS ( "pregnant women" ) OR TITLE-ABS ( men ) OR TITLE-ABS ( women ) OR TITLE-ABS ( "ethnic group" ) OR TITLE-ABS ( caucasian ) OR TITLE-ABS ( asian ) OR TITLE-ABS ( african ) OR TITLE-ABS ( "genetic polymorphism*" ) OR TITLE-ABS ( "individual susceptibility" ) OR TITLE-ABS ( "gene environment" ) OR TITLE-ABS ( "ethnic variability" ) OR TITLE-ABS ( "Afro American" ) OR TITLE-ABS ( hispanic ) OR TITLE-ABS ( "race difference" ) OR TITLE-ABS ( "age difference" ) OR TITLE-ABS ( "race differences" ) OR TITLE-ABS ( "age differences" ) OR TITLE-ABS ( "gender differences" ) OR TITLE-ABS ( "gender difference" ) OR TITLE-ABS ( "sex difference" ) OR TITLE-ABS ( "sex differences" ) ) AND ( TITLE-ABS-KEY ( auc ) OR TITLE-ABS-KEY ( area AND under AND the AND curve ) OR TITLE-ABS-KEY ( area AND under AND curve ) OR TITLE-ABS-KEY ( half AND life ) OR TITLE-ABS-KEY ( half-life ) OR TITLE-ABS-KEY ( half-lives ) OR TITLE-ABS-KEY ( clearance ) OR TITLE-ABS-KEY ( cmax ) OR TITLE-ABS-KEY ( vmax ) OR TITLE-ABS-KEY ( km ) OR TITLE-ABS-KEY ( "michaelis constant" ) OR TITLE-ABS-KEY ( pharmacokinetic ) OR TITLE-ABS-KEY ( pharmacokinetics ) OR TITLE-ABS-KEY ( toxicokinetic ) OR TITLE-ABS-KEY ( toxicokinetics ) ) AND NOT ( TITLE-ABS-KEY ( "cell line*" ) OR TITLE-ABS-KEY ( "cell culture*" ) OR TITLE-ABS-KEY ( rat ) OR TITLE-ABS-KEY(rats) OR TITLE-ABS-KEY ( mouse ) OR TITLE-ABS-KEY ( mice ) ) AND ( EXCLUDE ( DOCTYPE,"re" ) ) AND ( LIMIT-TO ( LANGUAGE,"English" ) )

*PubMed*

((((ezetimibe[Title/Abstract] OR ezetrol[Title/Abstract] OR zetia[Title/Abstract])) AND (auc[Title/Abstract] OR "area under the curve"[Title/Abstract] OR "area under curve"[Title/Abstract] OR "half life"[Title/Abstract] OR half-life[Title/Abstract] OR half-lives[Title/Abstract] OR clearance[Title/Abstract] OR cmax[Title/Abstract] OR vmax[Title/Abstract] OR km[Title/Abstract] OR "michaelis constant"[Title/Abstract] OR pharmacokinetic[Title/Abstract] OR pharmacokinetics[Title/Abstract] OR toxicokinetic[Title/Abstract] OR toxicokinetics)) AND (patient[Title/Abstract] OR patients[Title/Abstract] OR human[Title/Abstract] OR (adult[Title/Abstract]) OR adults[Title/Abstract] OR child[Title/Abstract] OR children[Title/Abstract] OR infant[Title/Abstract] OR neonate[Title/Abstract] OR newborn[Title/Abstract] OR newborns[Title/Abstract] OR elderly[Title/Abstract] OR "pregnant women"[Title/Abstract] OR men[Title/Abstract] OR women[Title/Abstract] OR "ethnic group"[Title/Abstract] OR caucasian[Title/Abstract] OR asian[Title/Abstract] OR african[Title/Abstract] OR "genetic polymorphism*"[Title/Abstract] OR "individual susceptibility"[Title/Abstract] OR "gene environment"[Title/Abstract] OR "ethnic variability"[Title/Abstract] OR "Afro American"[Title/Abstract] OR hispanic[Title/Abstract] OR "race difference"[Title/Abstract] OR "age difference"[Title/Abstract] OR "race differences"[Title/Abstract] OR "age differences"[Title/Abstract] OR "gender differences"[Title/Abstract] OR "gender difference"[Title/Abstract] OR "sex difference"[Title/Abstract] OR "sex differences"[Title/Abstract])) NOT ("cell line*"[Title/Abstract] OR "cell culture*"[Title/Abstract] OR rat[Title/Abstract] OR rats[Title/Abstract] OR mouse[Title/Abstract] OR mice[Title/Abstract])

***Mycophenolic acid***

*Scopus*

( TITLE-ABS ( "mycophenolic acid" )  OR  TITLE-ABS ( cellcept )  OR  TITLE-ABS ( mycophenolate )  OR  TITLE-ABS ( myfortic ) ) )  AND  (TITLE-ABS ( human )  OR  TITLE-ABS ( adult )  OR  TITLE-ABS ( adults )  OR  TITLE-ABS ( child )  OR  TITLE-ABS ( children )  OR  TITLE-ABS ( infant )  OR  TITLE-ABS ( neonate )  OR  TITLE-ABS ( newborn )  OR  TITLE-ABS ( newborns )  OR  TITLE-ABS ( elderly )  OR  TITLE-ABS ( "pregnant women" )  OR  TITLE-ABS ( men )  OR  TITLE-ABS ( women )  OR  TITLE-ABS ( "ethnic group" )  OR  TITLE-ABS ( caucasian )  OR  TITLE-ABS ( asian )  OR  TITLE-ABS ( african )  OR  TITLE-ABS ( "genetic polymorphism*" )  OR  TITLE-ABS ( "individual susceptibility" )  OR  TITLE-ABS ( "gene environment" )  OR  TITLE-ABS ( "ethnic variability" )  OR  TITLE-ABS ( "Afro American" )  OR  TITLE-ABS ( hispanic )  OR  TITLE-ABS ( "race difference" )  OR  TITLE-ABS ( "age difference" )  OR  TITLE-ABS ( "race differences" )  OR  TITLE-ABS ( "age differences" )  OR  TITLE-ABS ( "gender differences" )  OR  TITLE-ABS ( "gender difference" )  OR  TITLE-ABS ( "sex difference" )  OR  TITLE-ABS ( "sex differences" ) )  AND  ( TITLE-ABS-KEY ( auc )  OR  TITLE-ABS-KEY ( area  AND  under  AND  the  AND  curve )  OR  TITLE-ABS-KEY ( area  AND  under  AND  curve )  OR  TITLE-ABS-KEY ( half  AND  life )  OR  TITLE-ABS-KEY ( half-life )  OR  TITLE-ABS-KEY ( half-lives )  OR  TITLE-ABS-KEY ( clearance )  OR  TITLE-ABS-KEY ( cmax )  OR  TITLE-ABS-KEY ( pharmacokinetic )  OR  TITLE-ABS-KEY ( pharmacokinetics )  OR  TITLE-ABS-KEY ( toxicokinetic )  OR  TITLE-ABS-KEY ( toxicokinetics ) )  AND NOT  ( TITLE-ABS-KEY ( "cell line*" )  OR  TITLE-ABS-KEY ( "cell culture*" )  OR  TITLE-ABS-KEY ( "rat*" )  OR  TITLE-ABS-KEY ( mouse )  OR  TITLE-ABS-KEY ( mice ) )  AND  ( EXCLUDE ( DOCTYPE ,  "re" ) )  AND  ( LIMIT-TO ( LANGUAGE ,  "English" ) )

*PubMed*

("mycophenolic acid"[Title/Abstract] OR cellcept[Title/Abstract] OR mycophenolate[Title/Abstract] OR myfortic[Title/Abstract]) AND (Human[Title/Abstract] OR adult[Title/Abstract] OR child[Title/Abstract] OR children[Title/Abstract] OR infant[Title/Abstract] OR newborns[Title/Abstract] OR newborn[Title/Abstract] OR neonate[Title/Abstract] OR elderly[Title/Abstract] OR “pregnant women”[Title/Abstract] OR men[Title/Abstract] OR women[Title/Abstract] OR male[Title/Abstract] OR female[Title/Abstract] OR “ethnic groups”[Title/Abstract] OR “genetic polymorphism*"[Title/Abstract] OR "individual susceptibility"[Title/Abstract] OR "gene environment"[Title/Abstract] OR "ethnic variability"[Title/Abstract] OR Caucasian[Title/Abstract] OR Asian[Title/Abstract] OR "Afro American"[Title/Abstract] OR Hispanic[Title/Abstract] OR "race difference"[Title/Abstract] OR "age difference"[Title/Abstract] OR "race differences"[Title/Abstract] OR "age differences"[Title/Abstract] OR "gender differences"[Title/Abstract] OR "gender difference"[Title/Abstract] OR "sex difference"[Title/Abstract] OR "sex differences"[Title/Abstract]) AND (pharmacokinetic[Title/Abstract] OR pharmacokinetics[Title/Abstract] OR toxicokinetic[Title/Abstract] OR toxicokinetics[Title/Abstract] OR AUC[Title/Abstract] OR "area under the curve"[Title/Abstract] OR "area under curve"[Title/Abstract] OR half-life[Title/Abstract] OR "half life"[Title/Abstract] OR half-lives[Title/Abstract] OR clearance[Title/Abstract] OR cmax[Title/Abstract]) NOT ("cell line*"[Title/Abstract] OR "cell culture*"[Title/Abstract] OR rat[Title/Abstract] OR mouse[Title/Abstract]) AND (english[LANGUAGE])

***Oxazepam***

*Scopus*

(TITLE-ABS (oxazepam)) AND (TITLE-ABS(patient*) OR TITLE-ABS ( human ) OR TITLE-ABS ( adult ) OR TITLE-ABS ( adults ) OR TITLE-ABS ( child ) OR TITLE-ABS ( children ) OR TITLE-ABS ( infant ) OR TITLE-ABS ( neonate ) OR TITLE-ABS ( newborn ) OR TITLE-ABS ( newborns ) OR TITLE-ABS ( elderly ) OR TITLE-ABS ( "pregnant women" ) OR TITLE-ABS( men ) OR TITLE-ABS ( women ) OR TITLE-ABS ( "ethnic group" ) OR TITLE-ABS ( caucasian ) OR TITLE-ABS ( asian ) OR TITLE-ABS ( african ) OR TITLE-ABS ( "genetic polymorphism*" ) OR TITLE-ABS ( "individual susceptibility" ) OR TITLE-ABS ( "gene environment" ) OR TITLE-ABS ( "ethnic variability" ) OR TITLE-ABS ( "Afro American" ) OR TITLE-ABS ( hispanic ) OR TITLE-ABS ( "race difference" ) OR TITLE-ABS ( "age difference" ) OR TITLE-ABS ( "race differences" ) OR TITLE-ABS ( "age differences" ) OR TITLE-ABS ( "gender differences" ) OR TITLE-ABS ( "gender difference" ) OR TITLE-ABS ( "sex difference" ) OR TITLE-ABS ( "sex differences" )) AND (TITLE-ABS-KEY ( auc ) OR TITLE-ABS-KEY ( area AND under AND the AND curve ) OR TITLE-ABS-KEY ( area AND under AND curve ) OR TITLE-ABS-KEY ( half AND life ) OR TITLE-ABS-KEY ( half-life ) OR TITLE-ABS-KEY ( half-lives ) OR TITLE-ABS-KEY ( clearance ) OR TITLE-ABS-KEY ( cmax ) OR TITLE-ABS-KEY ( vmax ) OR TITLE-ABS-KEY ( km ) OR TITLE-ABS-KEY ( "michaelis constant" ) OR TITLE-ABS-KEY ( pharmacokinetic ) OR TITLE-ABS-KEY ( pharmacokinetics ) OR TITLE-ABS-KEY ( toxicokinetic ) OR TITLE-ABS-KEY ( toxicokinetics )) AND NOT ( TITLE-ABS-KEY ( "cell line*" ) OR TITLE-ABS-KEY ( "cell culture*" ) OR TITLE-ABS-KEY ( rat ) OR TITLE-ABS-KEY(rats) OR TITLE-ABS-KEY ( mouse ) OR TITLE-ABS-KEY ( mice ) ) AND ( EXCLUDE ( DOCTYPE,"re" ) ) AND ( LIMIT-TO ( LANGUAGE,"English" ) )

*PubMed*

((((oxazepam*[Title/Abstract])) AND (auc[Title/Abstract] OR "area under the curve"[Title/Abstract] OR "area under curve"[Title/Abstract] OR "half life"[Title/Abstract] OR half-life[Title/Abstract] OR half-lives[Title/Abstract] OR clearance[Title/Abstract] OR cmax[Title/Abstract] OR vmax[Title/Abstract] OR km[Title/Abstract] OR "michaelis constant"[Title/Abstract] OR pharmacokinetic[Title/Abstract] OR pharmacokinetics[Title/Abstract] OR toxicokinetic[Title/Abstract] OR toxicokinetics)) AND (patient[Title/Abstract] OR patients[Title/Abstract] OR human[Title/Abstract] OR (adult[Title/Abstract]) OR adults[Title/Abstract] OR child[Title/Abstract] OR children[Title/Abstract] OR infant[Title/Abstract] OR neonate[Title/Abstract] OR newborn[Title/Abstract] OR newborns[Title/Abstract] OR elderly[Title/Abstract] OR "pregnant women"[Title/Abstract] OR men[Title/Abstract] OR women[Title/Abstract] OR "ethnic group"[Title/Abstract] OR caucasian[Title/Abstract] OR asian[Title/Abstract] OR african[Title/Abstract] OR "genetic polymorphism*"[Title/Abstract] OR "individual susceptibility"[Title/Abstract] OR "gene environment"[Title/Abstract] OR "ethnic variability"[Title/Abstract] OR "Afro American"[Title/Abstract] OR hispanic[Title/Abstract] OR "race difference"[Title/Abstract] OR "age difference"[Title/Abstract] OR "race differences"[Title/Abstract] OR "age differences"[Title/Abstract] OR "gender differences"[Title/Abstract] OR "gender difference"[Title/Abstract] OR "sex difference"[Title/Abstract] OR "sex differences"[Title/Abstract])) NOT ("cell line*"[Title/Abstract] OR "cell culture*"[Title/Abstract] OR rat[Title/Abstract] OR rats[Title/Abstract] OR mouse[Title/Abstract] OR mice[Title/Abstract])

***Propofol***

*Scopus*

( TITLE-ABS ( propofol )  OR  TITLE-ABS ( aquafol )  OR  TITLE-ABS ( diprivan )  OR  TITLE-ABS ( disoprivan )  OR  TITLE-ABS ( disoprofol )  OR  TITLE-ABS ( fresofol )  OR  TITLE-ABS ( ivofol )  OR  TITLE-ABS ( recofol ) )  AND  ( TITLE-ABS ( patient* )  OR  TITLE-ABS ( human )  OR  TITLE-ABS ( adult )  OR  TITLE-ABS ( adults )  OR  TITLE-ABS ( child )  OR  TITLE-ABS ( children )  OR  TITLE-ABS ( infant )  OR  TITLE-ABS ( neonate )  OR  TITLE-ABS ( newborn )  OR  TITLE-ABS ( newborns )  OR  TITLE-ABS ( elderly )  OR  TITLE-ABS ( "pregnant women" )  OR  TITLE-ABS ( men )  OR  TITLE-ABS ( women )  OR  TITLE-ABS ( "ethnic group" )  OR  TITLE-ABS ( caucasian )  OR  TITLE-ABS ( asian )  OR  TITLE-ABS ( african )  OR  TITLE-ABS ( "genetic polymorphism*" )  OR  TITLE-ABS ( "individual susceptibility" )  OR  TITLE-ABS ( "gene environment" )  OR  TITLE-ABS ( "ethnic variability" )  OR  TITLE-ABS ( "Afro American" )  OR  TITLE-ABS ( hispanic )  OR  TITLE-ABS ( "race difference" )  OR  TITLE-ABS ( "age difference" )  OR  TITLE-ABS ( "race differences" )  OR  TITLE-ABS ( "age differences" )  OR  TITLE-ABS ( "gender differences" )  OR  TITLE-ABS ( "gender difference" )  OR  TITLE-ABS ( "sex difference" )  OR  TITLE-ABS ( "sex differences" ) )  AND  ( TITLE-ABS-KEY ( auc )  OR  TITLE-ABS-KEY ( area  AND  under  AND  the  AND  curve )  OR  TITLE-ABS-KEY ( area  AND  under  AND  curve )  OR  TITLE-ABS-KEY ( half  AND  life )  OR  TITLE-ABS-KEY ( half-life )  OR  TITLE-ABS-KEY ( half-lives )  OR  TITLE-ABS-KEY ( clearance )  OR  TITLE-ABS-KEY ( cmax )  OR  TITLE-ABS-KEY ( vmax )  OR  TITLE-ABS-KEY ( km )  OR  TITLE-ABS-KEY ( "michaelis constant" )  OR  TITLE-ABS-KEY ( pharmacokinetic )  OR  TITLE-ABS-KEY ( pharmacokinetics )  OR  TITLE-ABS-KEY ( toxicokinetic )  OR  TITLE-ABS-KEY ( toxicokinetics ) )  AND NOT  ( TITLE-ABS-KEY ( "cell line*" )  OR  TITLE-ABS-KEY ( "cell culture*" )  OR  TITLE-ABS-KEY ( rat* )  OR  TITLE-ABS-KEY ( mouse )  OR  TITLE-ABS-KEY ( mice ) )  AND  ( EXCLUDE ( DOCTYPE ,  "re" ) )  AND  ( LIMIT-TO ( LANGUAGE ,  "English" ) )

***Raltegravir***

*Scopus*

( ( TITLE-ABS ( raltegravir ) ) ) AND ( TITLE-ABS ( patient* ) OR TITLE-ABS ( human ) OR TITLE-ABS ( adult ) OR TITLE-ABS ( adults ) OR TITLE-ABS ( child ) OR TITLE-ABS ( children ) OR TITLE-ABS ( infant ) OR TITLE-ABS ( neonate ) OR TITLE-ABS ( newborn ) OR TITLE-ABS ( newborns ) OR TITLE-ABS ( elderly ) OR TITLE-ABS ( "pregnant women" ) OR TITLE-ABS ( men ) OR TITLE-ABS ( women ) OR TITLE-ABS ( "ethnic group" ) OR TITLE-ABS ( caucasian ) OR TITLE-ABS ( asian ) OR TITLE-ABS ( african ) OR TITLE-ABS ( "genetic polymorphism*" ) OR TITLE-ABS ( "individual susceptibility" ) OR TITLE-ABS ( "gene environment" ) OR TITLE-ABS ( "ethnic variability" ) OR TITLE-ABS ( "Afro American" ) OR TITLE-ABS ( hispanic ) OR TITLE-ABS ( "race difference" ) OR TITLE-ABS ( "age difference" ) OR TITLE-ABS ( "race differences" ) OR TITLE-ABS ( "age differences" ) OR TITLE-ABS ( "gender differences" ) OR TITLE-ABS ( "gender difference" ) OR TITLE-ABS ( "sex difference" ) OR TITLE-ABS ( "sex differences" ) ) AND ( TITLE-ABS-KEY ( auc ) OR TITLE-ABS-KEY ( area AND under AND the AND curve ) OR TITLE-ABS-KEY ( area AND under AND curve ) OR TITLE-ABS-KEY ( half AND life ) OR TITLE-ABS-KEY ( half-life ) OR TITLE-ABS-KEY ( half-lives ) OR TITLE-ABS-KEY ( clearance ) OR TITLE-ABS-KEY ( cmax ) OR TITLE-ABS-KEY ( vmax ) OR TITLE-ABS-KEY ( km ) OR TITLE-ABS-KEY ( "michaelis constant" ) OR TITLE-ABS-KEY ( pharmacokinetic ) OR TITLE-ABS-KEY ( pharmacokinetics ) OR TITLE-ABS-KEY ( toxicokinetic ) OR TITLE-ABS-KEY ( toxicokinetics ) ) AND NOT ( TITLE-ABS-KEY ( "cell line*" ) OR TITLE-ABS-KEY ( "cell culture*" ) OR TITLE-ABS-KEY ( rat) OR TITLE-ABS-KEY(rats) OR TITLE-ABS-KEY ( mouse ) OR TITLE-ABS-KEY ( mice ) ) AND ( EXCLUDE ( DOCTYPE,"re" ) ) AND ( LIMIT-TO ( LANGUAGE,"English" ) )

*PubMed*

((((raltegravir[Title/Abstract])) AND (auc[Title/Abstract] OR "area under the curve"[Title/Abstract] OR "area under curve"[Title/Abstract] OR "half life"[Title/Abstract] OR half-life[Title/Abstract] OR half-lives[Title/Abstract] OR clearance[Title/Abstract] OR cmax[Title/Abstract] OR vmax[Title/Abstract] OR km[Title/Abstract] OR "michaelis constant"[Title/Abstract] OR pharmacokinetic[Title/Abstract] OR pharmacokinetics[Title/Abstract] OR toxicokinetic[Title/Abstract] OR toxicokinetics)) AND (patient[Title/Abstract] OR patients[Title/Abstract] OR human[Title/Abstract] OR (adult[Title/Abstract]) OR adults[Title/Abstract] OR child[Title/Abstract] OR children[Title/Abstract] OR infant[Title/Abstract] OR neonate[Title/Abstract] OR newborn[Title/Abstract] OR newborns[Title/Abstract] OR elderly[Title/Abstract] OR "pregnant women"[Title/Abstract] OR men[Title/Abstract] OR women[Title/Abstract] OR "ethnic group"[Title/Abstract] OR caucasian[Title/Abstract] OR asian[Title/Abstract] OR african[Title/Abstract] OR "genetic polymorphism*"[Title/Abstract] OR "individual susceptibility"[Title/Abstract] OR "gene environment"[Title/Abstract] OR "ethnic variability"[Title/Abstract] OR "Afro American"[Title/Abstract] OR hispanic[Title/Abstract] OR "race difference"[Title/Abstract] OR "age difference"[Title/Abstract] OR "race differences"[Title/Abstract] OR "age differences"[Title/Abstract] OR "gender differences"[Title/Abstract] OR "gender difference"[Title/Abstract] OR "sex difference"[Title/Abstract] OR "sex differences"[Title/Abstract])) NOT ("cell line*"[Title/Abstract] OR "cell culture*"[Title/Abstract] OR rat[Title/Abstract] OR rats[Title/Abstract] OR mouse[Title/Abstract] OR mice[Title/Abstract])

***SN38***

*Scopus*

( ( TITLE-ABS ( sn38 )  OR  TITLE-ABS ( 7-ethyl-10-hydroxycamptothecin )  OR  TITLE-ABS ( "SN 38" )  OR  TITLE-ABS ( sn-38 ) ) )  AND  ( TITLE-ABS ( patient* )  OR  TITLE-ABS ( human )  OR  TITLE-ABS ( adult )  OR  TITLE-ABS ( adults )  OR  TITLE-ABS ( child )  OR  TITLE-ABS ( children )  OR  TITLE-ABS ( infant )  OR  TITLE-ABS ( neonate )  OR  TITLE-ABS ( newborn )  OR  TITLE-ABS ( newborns )  OR  TITLE-ABS ( elderly )  OR  TITLE-ABS ( "pregnant women" )  OR  TITLE-ABS ( men )  OR  TITLE-ABS ( women )  OR  TITLE-ABS ( "ethnic group" )  OR  TITLE-ABS ( caucasian )  OR  TITLE-ABS ( asian )  OR  TITLE-ABS ( african )  OR  TITLE-ABS ( "genetic polymorphism*" )  OR  TITLE-ABS ( "individual susceptibility" )  OR  TITLE-ABS ( "gene environment" )  OR  TITLE-ABS ( "ethnic variability" )  OR  TITLE-ABS ( "Afro American" )  OR  TITLE-ABS ( hispanic )  OR  TITLE-ABS ( "race difference" )  OR  TITLE-ABS ( "age difference" )  OR  TITLE-ABS ( "race differences" )  OR  TITLE-ABS ( "age differences" )  OR  TITLE-ABS ( "gender differences" )  OR  TITLE-ABS ( "gender difference" )  OR  TITLE-ABS ( "sex difference" )  OR  TITLE-ABS ( "sex differences" ) )  AND  ( TITLE-ABS-KEY ( auc )  OR  TITLE-ABS-KEY ( area  AND  under  AND  the  AND  curve )  OR  TITLE-ABS-KEY ( area  AND  under  AND  curve )  OR  TITLE-ABS-KEY ( half  AND  life )  OR  TITLE-ABS-KEY ( half-life )  OR  TITLE-ABS-KEY ( half-lives )  OR  TITLE-ABS-KEY ( clearance )  OR  TITLE-ABS-KEY ( cmax )  OR  TITLE-ABS-KEY ( vmax )  OR  TITLE-ABS-KEY ( km )  OR  TITLE-ABS-KEY ( "michaelis constant" )  OR  TITLE-ABS-KEY ( pharmacokinetic )  OR  TITLE-ABS-KEY ( pharmacokinetics )  OR  TITLE-ABS-KEY ( toxicokinetic )  OR  TITLE-ABS-KEY ( toxicokinetics ) )  AND NOT  ( TITLE-ABS-KEY ( "cell line*" )  OR  TITLE-ABS-KEY ( "cell culture*" )  OR  TITLE-ABS-KEY ( rat )  OR  TITLE-ABS-KEY ( rats )  OR  TITLE-ABS-KEY ( mouse )  OR  TITLE-ABS-KEY ( mice ) )  AND  ( EXCLUDE ( DOCTYPE ,  "re" ) )  AND  ( LIMIT-TO ( LANGUAGE ,  "English" ) )

*PubMed*

((((sn38[title/abstract] or 7-ethyl-10-hydroxycamptothecin[title/abstract] or "sn 38"[title/abstract] or sn-38[title/abstract])) and (auc[title/abstract] or "area under the curve"[title/abstract] or "area under curve"[title/abstract] or "half life"[title/abstract] or half-life[title/abstract] or half-lives[title/abstract] or clearance[title/abstract] or cmax[title/abstract] or vmax[title/abstract] or km[title/abstract] or "michaelis constant"[title/abstract] or pharmacokinetic[title/abstract] or pharmacokinetics[title/abstract] or toxicokinetic[title/abstract] or toxicokinetics)) and (patient[title/abstract] or patients[title/abstract] or human[title/abstract] or (adult[title/abstract]) or adults[title/abstract] or child[title/abstract] or children[title/abstract] or infant[title/abstract] or neonate[title/abstract] or newborn[title/abstract] or newborns[title/abstract] or elderly[title/abstract] or "pregnant women"[title/abstract] or men[title/abstract] or women[title/abstract] or "ethnic group"[title/abstract] or caucasian[title/abstract] or asian[title/abstract] or african[title/abstract] or "genetic polymorphism*"[title/abstract] or "individual susceptibility"[title/abstract] or "gene environment"[title/abstract] or "ethnic variability"[title/abstract] or "afro american"[title/abstract] or hispanic[title/abstract] or "race difference"[title/abstract] or "age difference"[title/abstract] or "race differences"[title/abstract] or "age differences"[title/abstract] or "gender differences"[title/abstract] or "gender difference"[title/abstract] or "sex difference"[title/abstract] or "sex differences"[title/abstract])) not ("cell line*"[title/abstract] or "cell culture*"[title/abstract] or rat[title/abstract] or rats[title/abstract] or mouse[title/abstract] or mice[title/abstract]) and (english[language]) and not (review[PT])

***Telmisartan***

*Scopus*

( ( TITLE-ABS ( telmisartan )  OR  TITLE-ABS ( micardis )  OR  TITLE-ABS ( pritor ) ) )  AND  ( TITLE-ABS ( patient* )  OR  TITLE-ABS ( human )  OR  TITLE-ABS ( adult )  OR  TITLE-ABS ( adults )  OR  TITLE-ABS ( child )  OR  TITLE-ABS ( children )  OR  TITLE-ABS ( infant )  OR  TITLE-ABS ( neonate )  OR  TITLE-ABS ( newborn )  OR  TITLE-ABS ( newborns )  OR  TITLE-ABS ( elderly )  OR  TITLE-ABS ( "pregnant women" )  OR  TITLE-ABS ( men )  OR  TITLE-ABS ( women )  OR  TITLE-ABS ( "ethnic group" )  OR  TITLE-ABS ( caucasian )  OR  TITLE-ABS ( asian )  OR  TITLE-ABS ( african )  OR  TITLE-ABS ( "genetic polymorphism*" )  OR  TITLE-ABS ( "individual susceptibility" )  OR  TITLE-ABS ( "gene environment" )  OR  TITLE-ABS ( "ethnic variability" )  OR  TITLE-ABS ( "Afro American" )  OR  TITLE-ABS ( hispanic )  OR  TITLE-ABS ( "race difference" )  OR  TITLE-ABS ( "age difference" )  OR  TITLE-ABS ( "race differences" )  OR  TITLE-ABS ( "age differences" )  OR  TITLE-ABS ( "gender differences" )  OR  TITLE-ABS ( "gender difference" )  OR  TITLE-ABS ( "sex difference" )  OR  TITLE-ABS ( "sex differences" ) )  AND  ( TITLE-ABS-KEY ( auc )  OR  TITLE-ABS-KEY ( area  AND  under  AND  the  AND  curve )  OR  TITLE-ABS-KEY ( area  AND  under  AND  curve )  OR  TITLE-ABS-KEY ( half  AND  life )  OR  TITLE-ABS-KEY ( half-life )  OR  TITLE-ABS-KEY ( half-lives )  OR  TITLE-ABS-KEY ( clearance )  OR  TITLE-ABS-KEY ( cmax )  OR  TITLE-ABS-KEY ( vmax )  OR  TITLE-ABS-KEY ( km )  OR  TITLE-ABS-KEY ( "michaelis constant" )  OR  TITLE-ABS-KEY ( pharmacokinetic )  OR  TITLE-ABS-KEY ( pharmacokinetics )  OR  TITLE-ABS-KEY ( toxicokinetic )  OR  TITLE-ABS-KEY ( toxicokinetics ) )  AND NOT  ( TITLE-ABS-KEY ( "cell line*" )  OR  TITLE-ABS-KEY ( "cell culture*" )  OR  TITLE-ABS-KEY ( rat* )  OR  TITLE-ABS-KEY ( mouse )  OR  TITLE-ABS-KEY ( mice ) )  AND  ( EXCLUDE ( DOCTYPE ,  "re" ) )  AND  ( LIMIT-TO ( LANGUAGE ,  "English" ) )

*PubMed*

((((telmisartan[Title/Abstract] OR micardis[Title/Abstract] OR pritor[Title/Abstract])) AND (auc[Title/Abstract] OR "area under the curve"[Title/Abstract] OR "area under curve"[Title/Abstract] OR "half life"[Title/Abstract] OR half-life[Title/Abstract] OR half-lives[Title/Abstract] OR clearance[Title/Abstract] OR cmax[Title/Abstract] OR vmax[Title/Abstract] OR km[Title/Abstract] OR "michaelis constant"[Title/Abstract] OR pharmacokinetic[Title/Abstract] OR pharmacokinetics[Title/Abstract] OR toxicokinetic[Title/Abstract] OR toxicokinetics)) AND (patient[Title/Abstract] OR patients[Title/Abstract] OR human[Title/Abstract] OR (adult[Title/Abstract]) OR adults[Title/Abstract] OR child[Title/Abstract] OR children[Title/Abstract] OR infant[Title/Abstract] OR neonate[Title/Abstract] OR newborn[Title/Abstract] OR newborns[Title/Abstract] OR elderly[Title/Abstract] OR "pregnant women"[Title/Abstract] OR men[Title/Abstract] OR women[Title/Abstract] OR "ethnic group"[Title/Abstract] OR caucasian[Title/Abstract] OR asian[Title/Abstract] OR african[Title/Abstract] OR "genetic polymorphism*"[Title/Abstract] OR "individual susceptibility"[Title/Abstract] OR "gene environment"[Title/Abstract] OR "ethnic variability"[Title/Abstract] OR "Afro American"[Title/Abstract] OR hispanic[Title/Abstract] OR "race difference"[Title/Abstract] OR "age difference"[Title/Abstract] OR "race differences"[Title/Abstract] OR "age differences"[Title/Abstract] OR "gender differences"[Title/Abstract] OR "gender difference"[Title/Abstract] OR "sex difference"[Title/Abstract] OR "sex differences"[Title/Abstract])) NOT ("cell line*"[Title/Abstract] OR "cell culture*"[Title/Abstract] OR rat[Title/Abstract] OR rats[Title/Abstract] OR mouse[Title/Abstract] OR mice[Title/Abstract])

***Trifluoperazine***

*Scopus*

( TITLE-ABS ( trifluoperazine ) OR TITLE-ABS ( eskazine ) OR TITLE-ABS ( flupazine ) OR TITLE-ABS ( stelazine ) OR TITLE-ABS ( terfluzine ) OR TITLE-ABS ( trifluperazine ) OR TITLE-ABS ( triftazin ) ) AND ( TITLE-ABS ( patient* ) OR TITLE-ABS ( human ) OR TITLE-ABS ( adult ) OR TITLE-ABS ( adults ) OR TITLE-ABS ( child ) OR TITLE-ABS ( children ) OR TITLE-ABS ( infant ) OR TITLE-ABS ( neonate ) OR TITLE-ABS ( newborn ) OR TITLE-ABS ( newborns ) OR TITLE-ABS ( elderly ) OR TITLE-ABS ( "pregnant women" ) OR TITLE-ABS ( men ) OR TITLE-ABS ( women ) OR TITLE-ABS ( "ethnic group" ) OR TITLE-ABS ( caucasian ) OR TITLE-ABS ( asian ) OR TITLE-ABS ( african ) OR TITLE-ABS ( "genetic polymorphism*" ) OR TITLE-ABS ( "individual susceptibility" ) OR TITLE-ABS ( "gene environment" ) OR TITLE-ABS ( "ethnic variability" ) OR TITLE-ABS ( "Afro American" ) OR TITLE-ABS ( hispanic ) OR TITLE-ABS ( "race difference" ) OR TITLE-ABS ( "age difference" ) OR TITLE-ABS ( "race differences" ) OR TITLE-ABS ( "age differences" ) OR TITLE-ABS ( "gender differences" ) OR TITLE-ABS ( "gender difference" ) OR TITLE-ABS ( "sex difference" ) OR TITLE-ABS ( "sex differences" ) ) AND ( TITLE-ABS-KEY ( auc ) OR TITLE-ABS-KEY ( area AND under AND the AND curve ) OR TITLE-ABS-KEY ( area AND under AND curve ) OR TITLE-ABS-KEY ( half AND life ) OR TITLE-ABS-KEY ( half-life ) OR TITLE-ABS-KEY ( half-lives ) OR TITLE-ABS-KEY ( clearance ) OR TITLE-ABS-KEY ( cmax ) OR TITLE-ABS-KEY ( vmax ) OR TITLE-ABS-KEY ( km ) OR TITLE-ABS-KEY ( "michaelis constant" ) OR TITLE-ABS-KEY ( pharmacokinetic ) OR TITLE-ABS-KEY ( pharmacokinetics ) OR TITLE-ABS-KEY ( toxicokinetic ) OR TITLE-ABS-KEY ( toxicokinetics ) ) AND NOT ( TITLE-ABS-KEY ( "cell line*" ) OR TITLE-ABS-KEY ( "cell culture*" ) OR TITLE-ABS-KEY ( rat) OR TITLE-ABS-KEY(rats) OR TITLE-ABS-KEY ( mouse ) OR TITLE-ABS-KEY ( mice ) ) AND ( EXCLUDE ( DOCTYPE,"re" ) ) AND ( LIMIT-TO ( LANGUAGE,"English" ) )

*PubMed*

((((trifluoperazine[Title/Abstract] OR eskazine[Title/Abstract] OR flupazine[Title/Abstract] OR stelazine[Title/Abstract] OR terfluzine[Title/Abstract] OR trifluperazine[Title/Abstract] OR triftazin[Title/Abstract])) AND (auc[Title/Abstract] OR "area under the curve"[Title/Abstract] OR "area under curve"[Title/Abstract] OR "half life"[Title/Abstract] OR half-life[Title/Abstract] OR half-lives[Title/Abstract] OR clearance[Title/Abstract] OR cmax[Title/Abstract] OR vmax[Title/Abstract] OR km[Title/Abstract] OR "michaelis constant"[Title/Abstract] OR pharmacokinetic[Title/Abstract] OR pharmacokinetics[Title/Abstract] OR toxicokinetic[Title/Abstract] OR toxicokinetics)) AND (patient[Title/Abstract] OR patients[Title/Abstract] OR human[Title/Abstract] OR (adult[Title/Abstract]) OR adults[Title/Abstract] OR child[Title/Abstract] OR children[Title/Abstract] OR infant[Title/Abstract] OR neonate[Title/Abstract] OR newborn[Title/Abstract] OR newborns[Title/Abstract] OR elderly[Title/Abstract] OR "pregnant women"[Title/Abstract] OR men[Title/Abstract] OR women[Title/Abstract] OR "ethnic group"[Title/Abstract] OR caucasian[Title/Abstract] OR asian[Title/Abstract] OR african[Title/Abstract] OR "genetic polymorphism*"[Title/Abstract] OR "individual susceptibility"[Title/Abstract] OR "gene environment"[Title/Abstract] OR "ethnic variability"[Title/Abstract] OR "Afro American"[Title/Abstract] OR hispanic[Title/Abstract] OR "race difference"[Title/Abstract] OR "age difference"[Title/Abstract] OR "race differences"[Title/Abstract] OR "age differences"[Title/Abstract] OR "gender differences"[Title/Abstract] OR "gender difference"[Title/Abstract] OR "sex difference"[Title/Abstract] OR "sex differences"[Title/Abstract])) NOT ("cell line*"[Title/Abstract] OR "cell culture*"[Title/Abstract] OR rat[Title/Abstract] OR rats[Title/Abstract] OR mouse[Title/Abstract] OR mice[Title/Abstract])

***Selection criteria***

For some compounds, data on non-healthy individuals were available and these data were included in the meta-analysis. For raltegravir and zidovudine, human immunodeficiency virus (HIV) patients were included, for SN38, cancer patients were included, and for deferiprone, thalassemia patients were included. For all compounds which included PK data from patients, liver and kidney values such as creatinine, ALT and AST levels, had to be within normal range or clinically accepted range. Volunteers wild mild or moderate impairment (as indicated by the specific study) were still included. For ethinylestradiol, combinations with other hormones that are commonly found in anticonception drugs were not excluded from the analysis, assuming that these other compounds do not influence the kinetics of ethinylestradiol. For all other compounds, studies where subjects were administered combinations of substrates were excluded.
