# Supplementary material for: Human variability in isoform-specific UDP-glucuronosyltransferases: markers of acute and chronic exposure, polymorphisms and uncertainty factors
Source: Arch Toxicol. 2020 May 15;94(8):2637–61. doi: 10.1007/s00204-020-02765-8 (PMC7395075; doi:10.1007/s00204-020-02765-8)

**Supplementary Material 2 –** *PRISMA Flow Diagrams for compounds included in this study*^1^

^1^No PRISMA diagram is included for Zidovudine, because data was extracted mostly from another study, see Methods section.

An additional search in Google Scholar was performed for the compounds with keywords ‘pharmacokinetics’, ‘healthy’, ‘[compound]’, to retrieve more pharmacokinetic studies in humans.


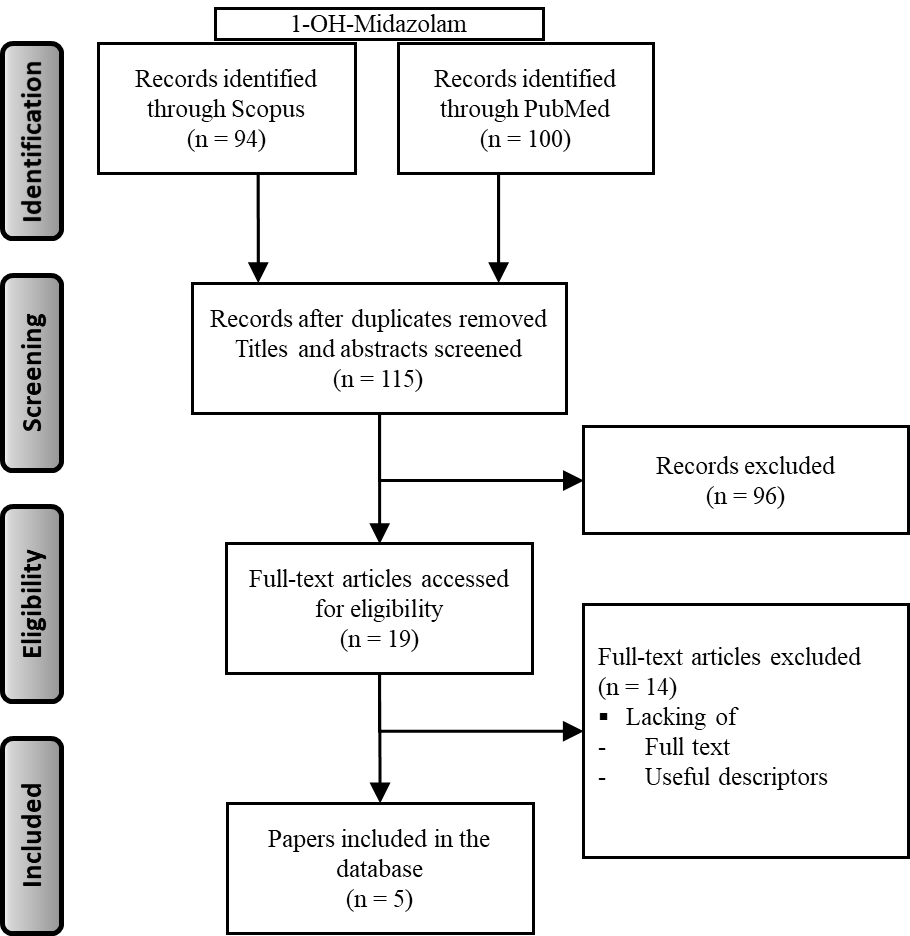


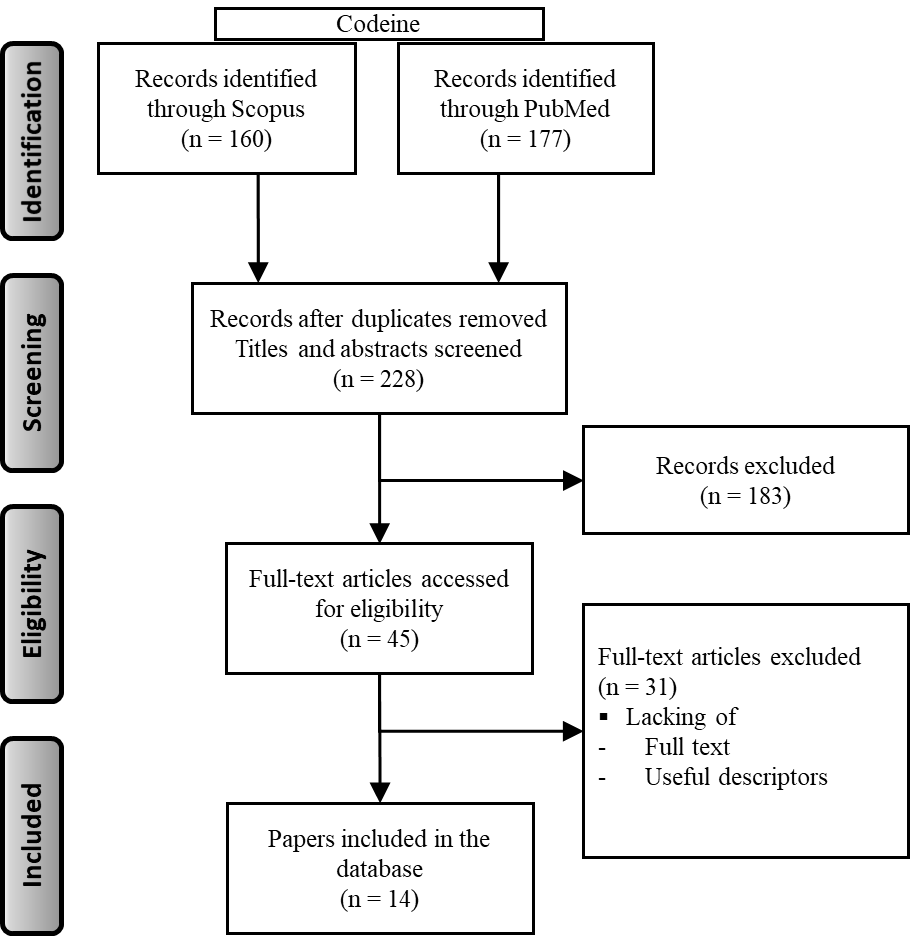


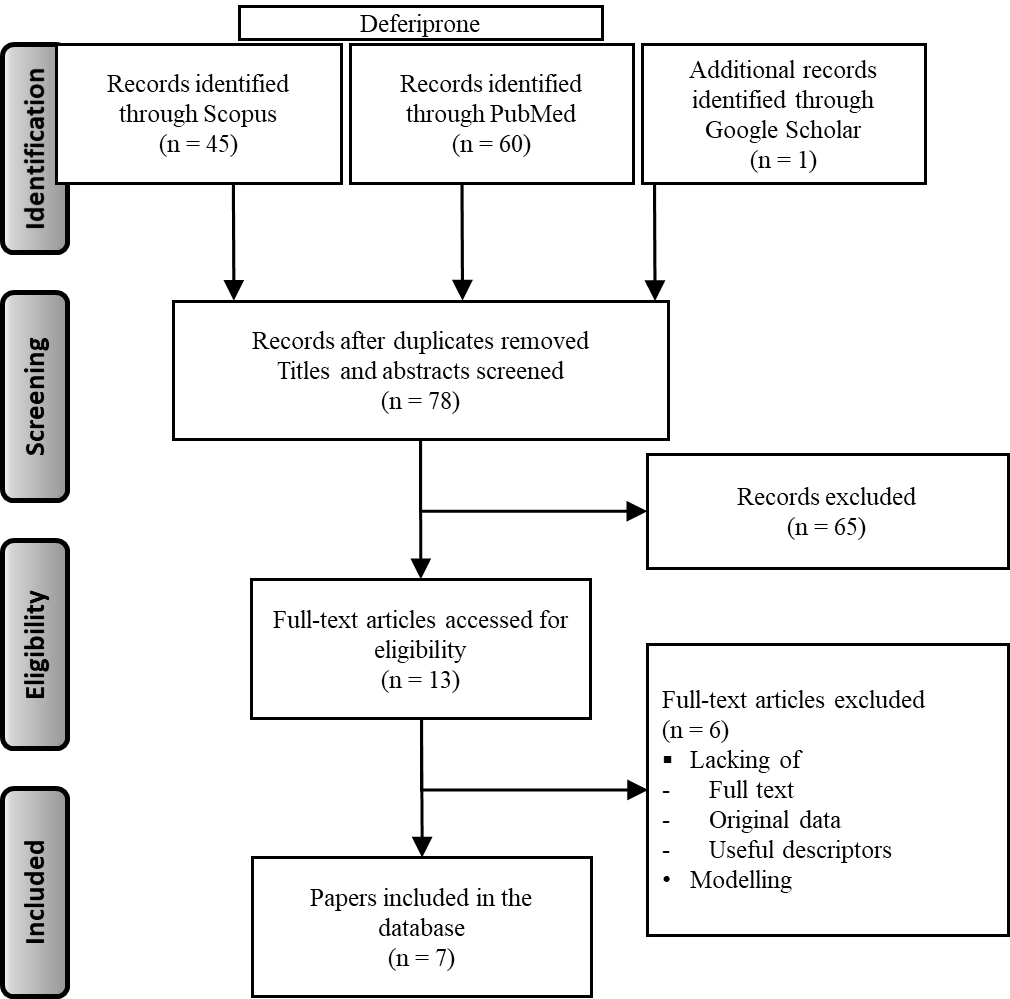


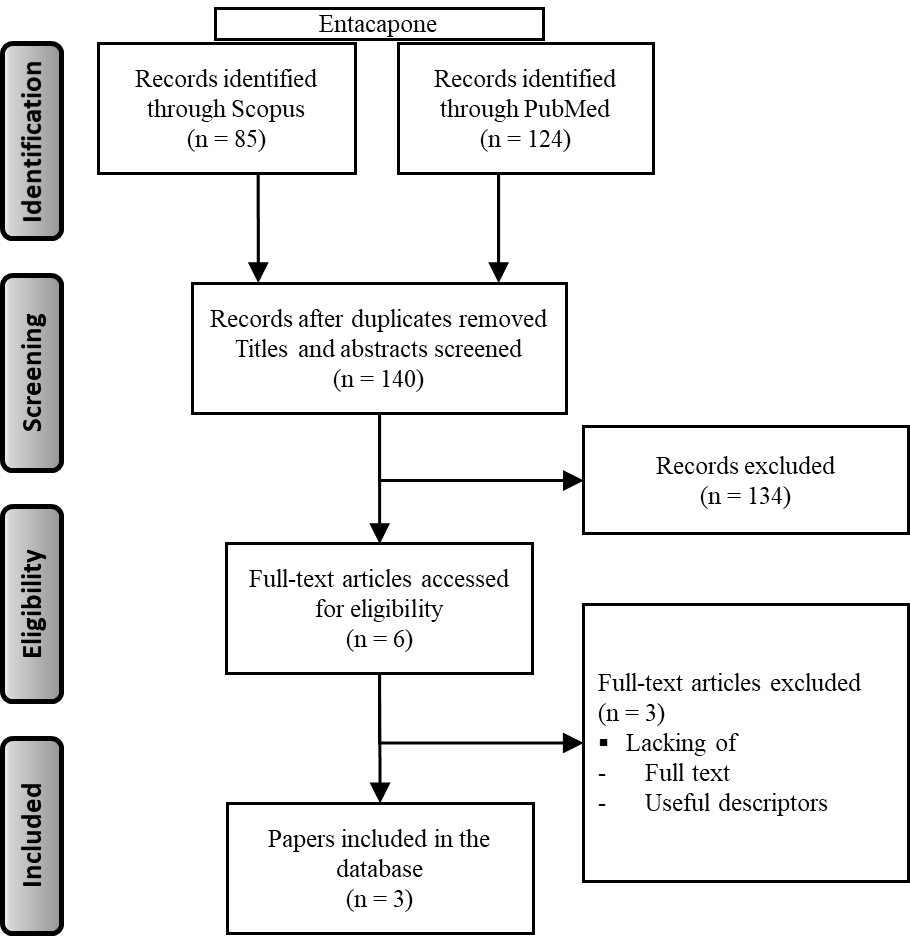


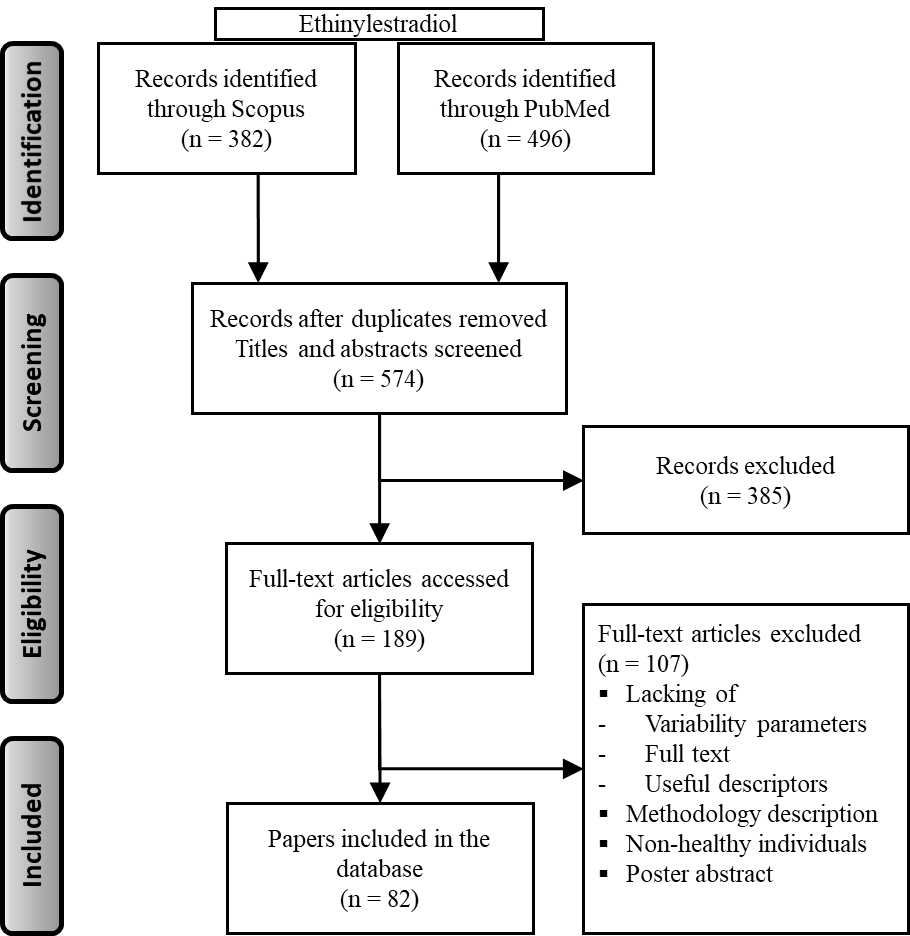


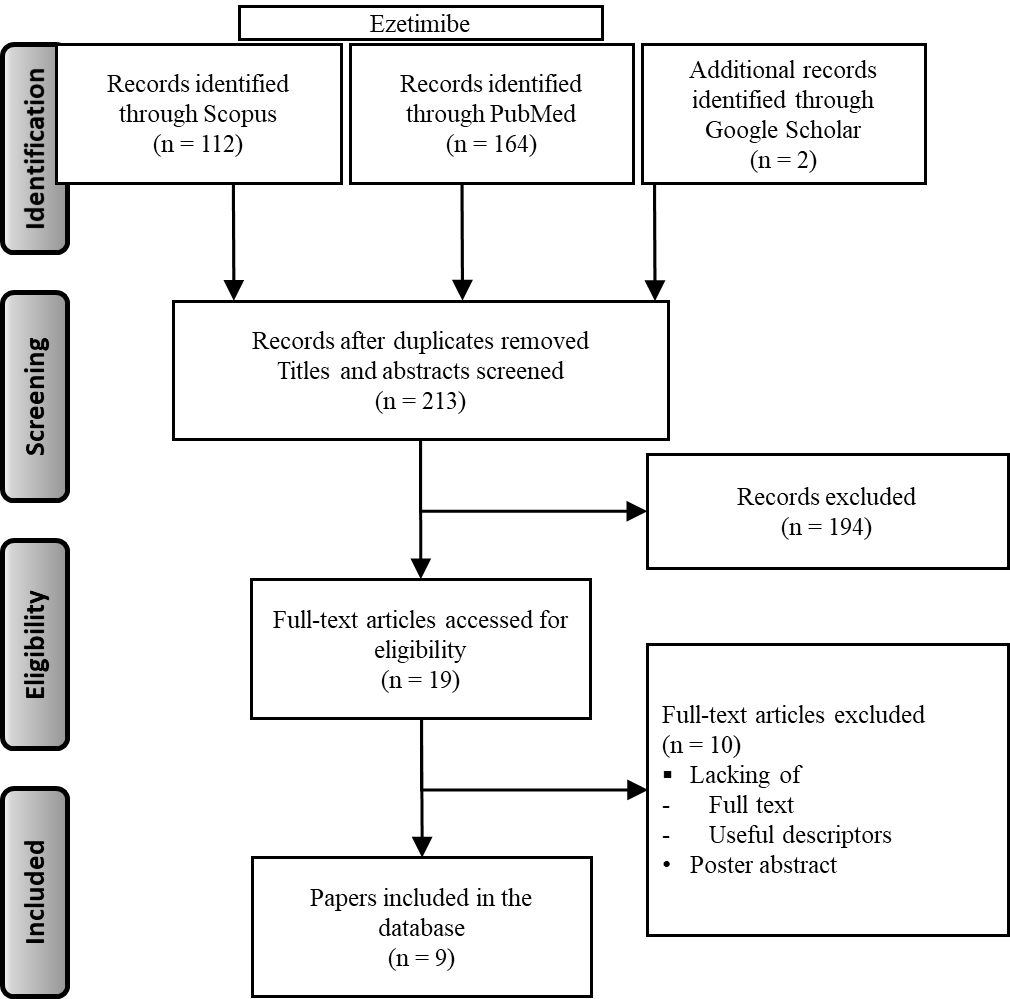


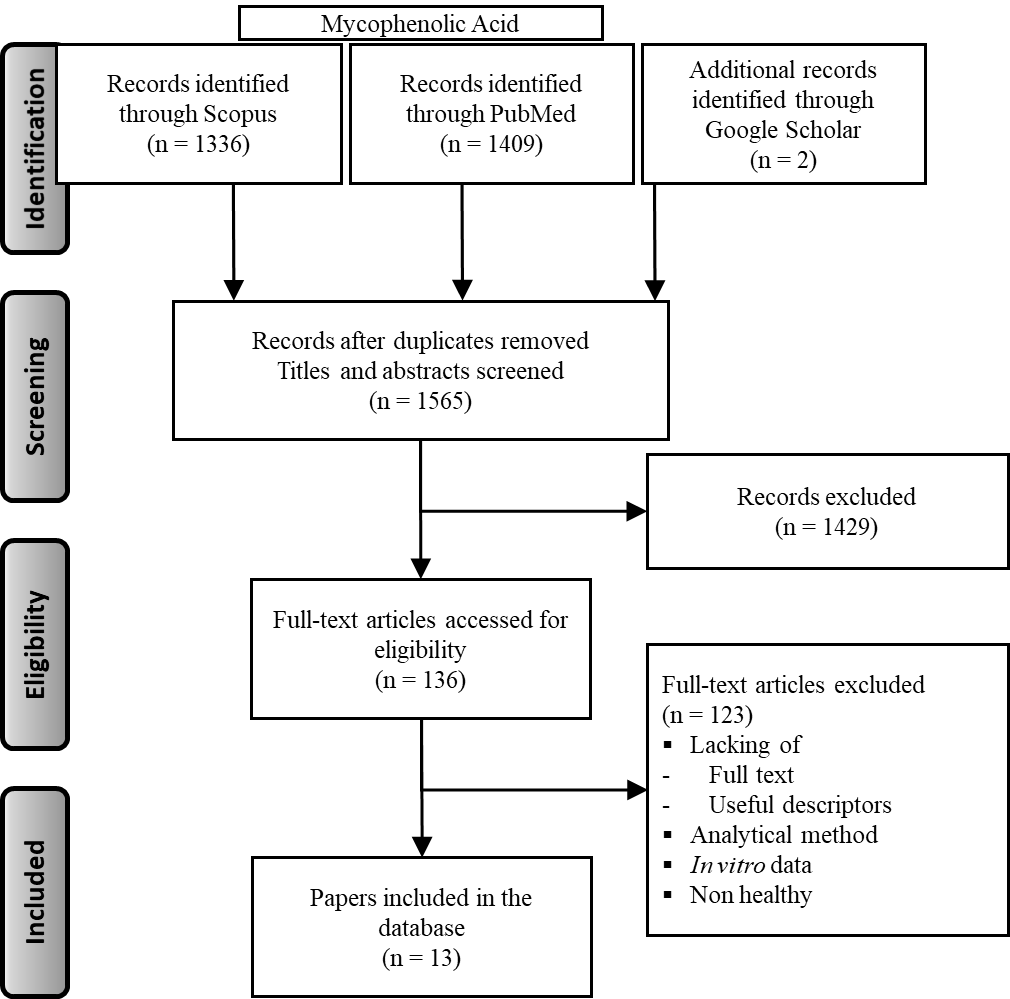


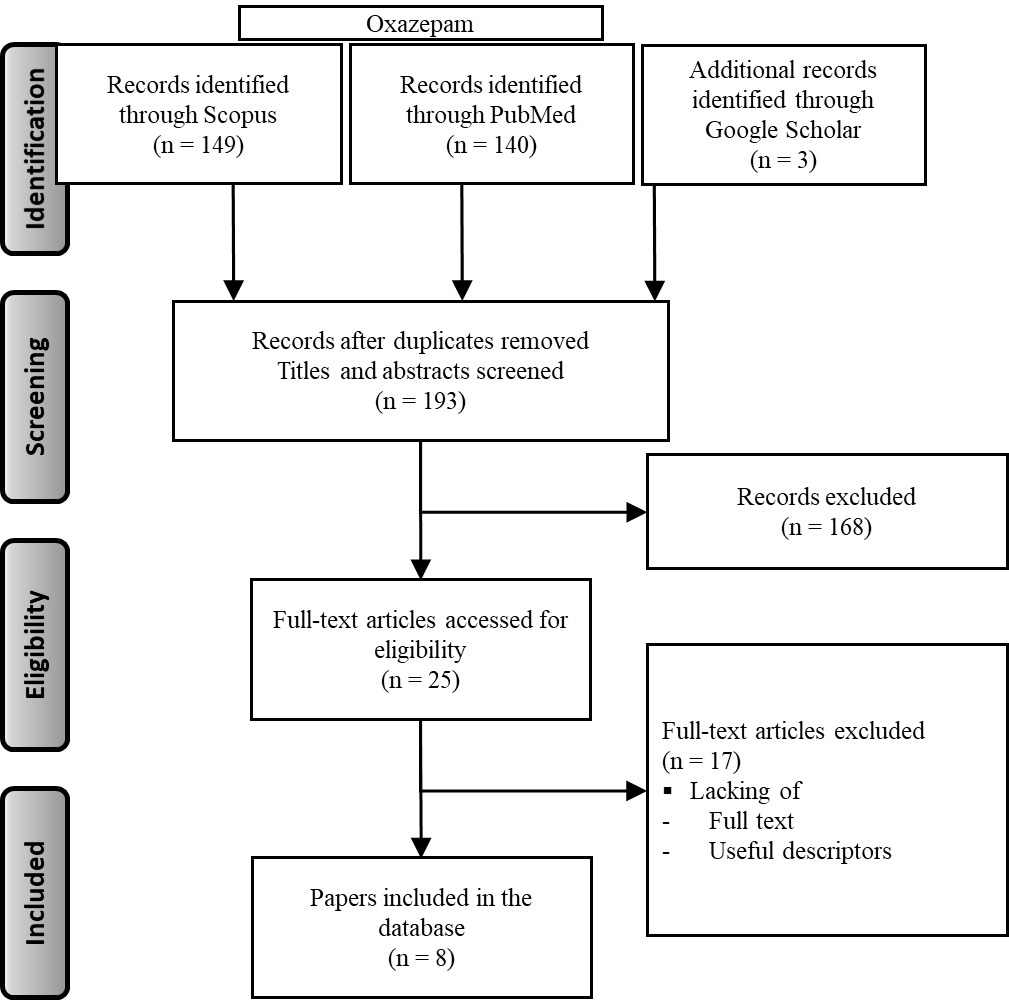


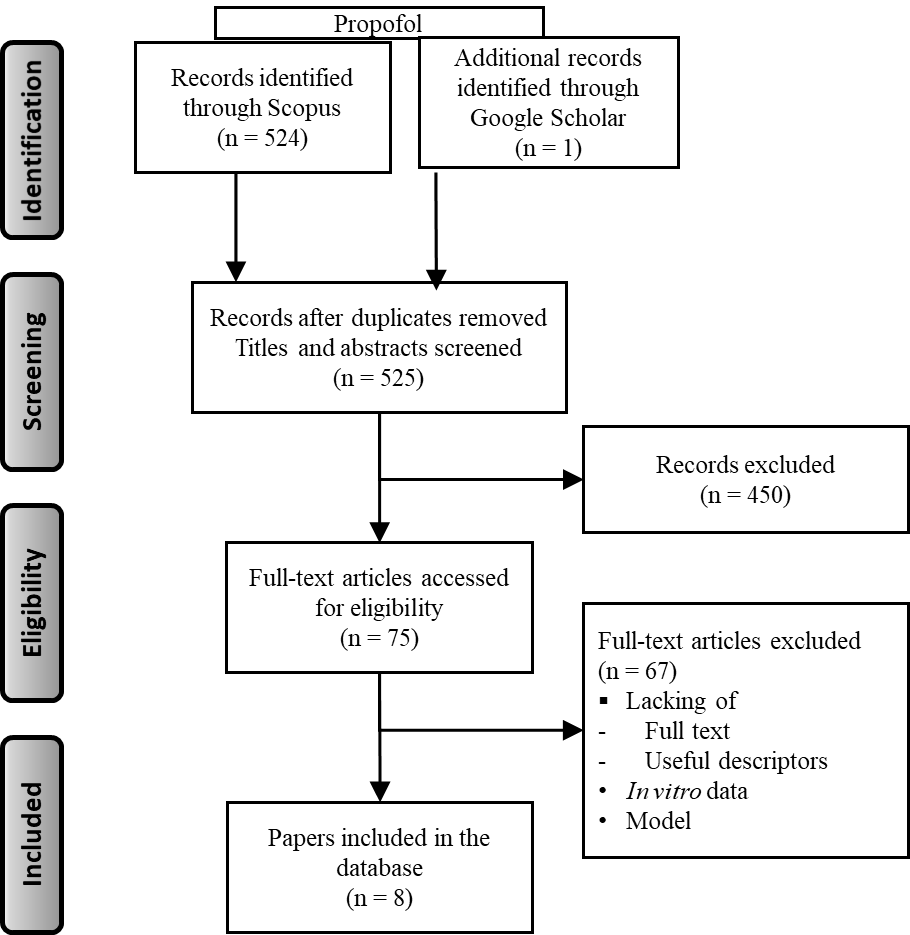


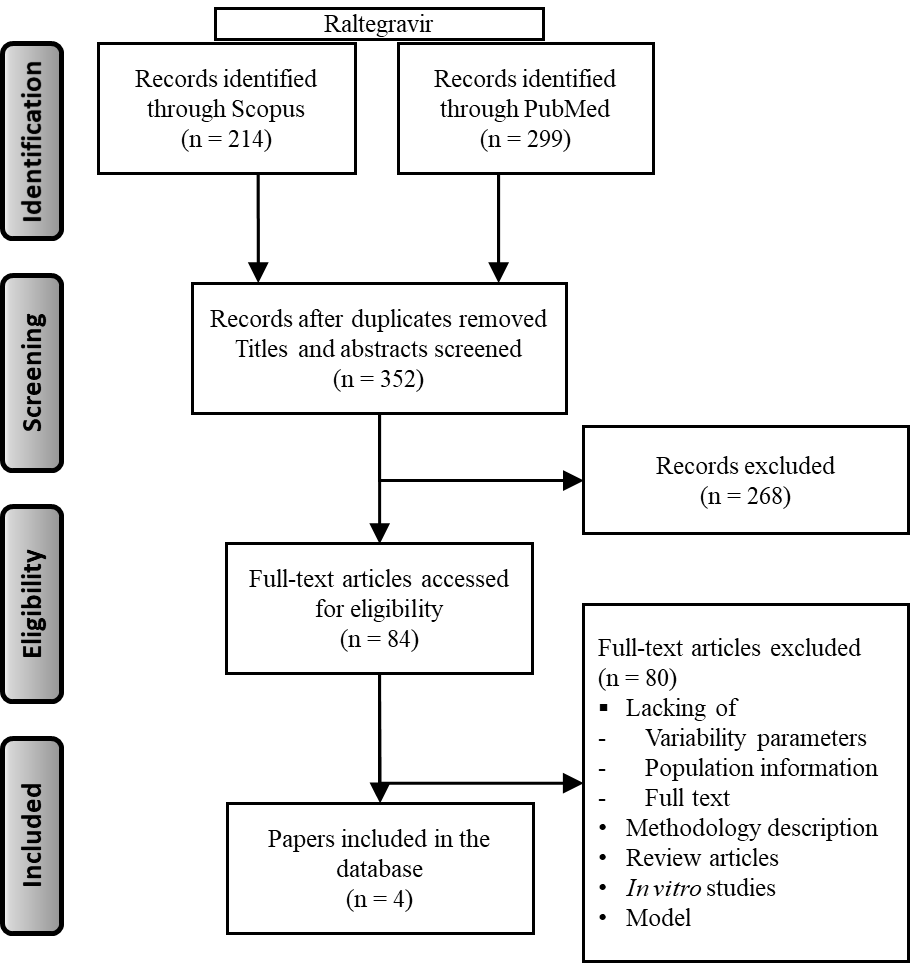


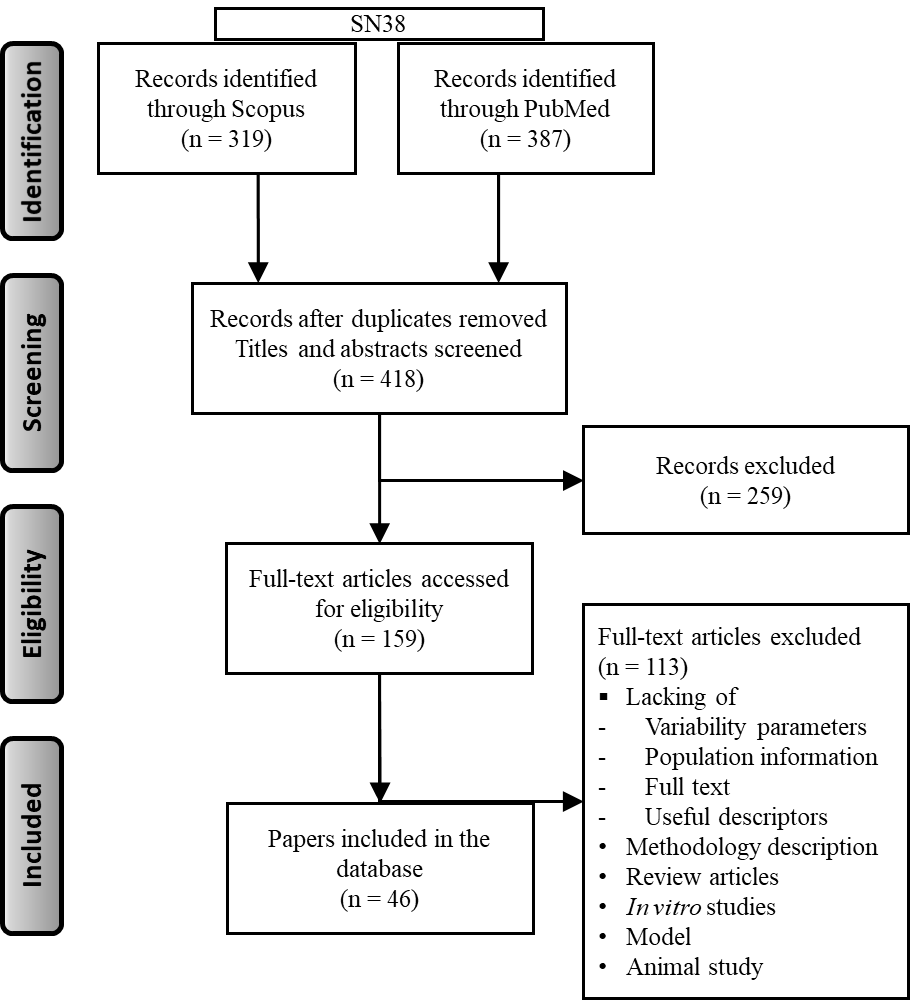


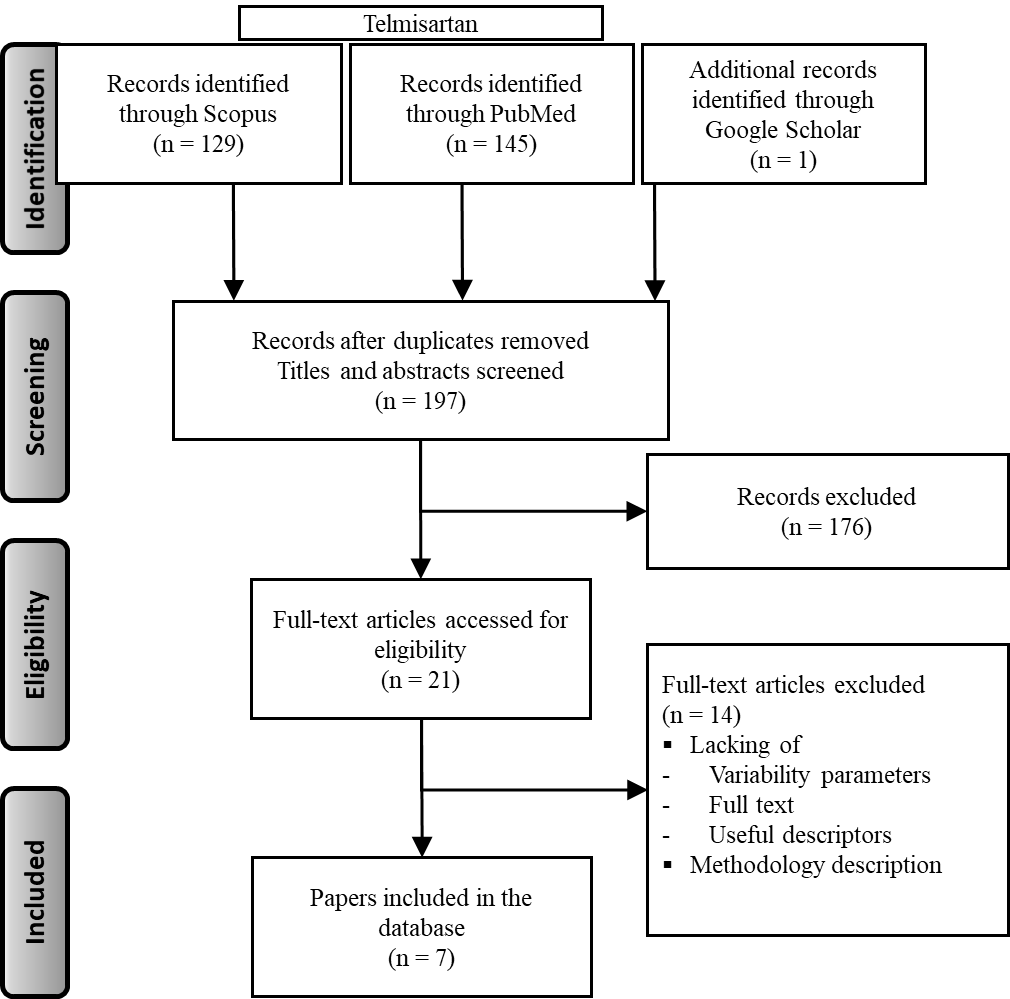


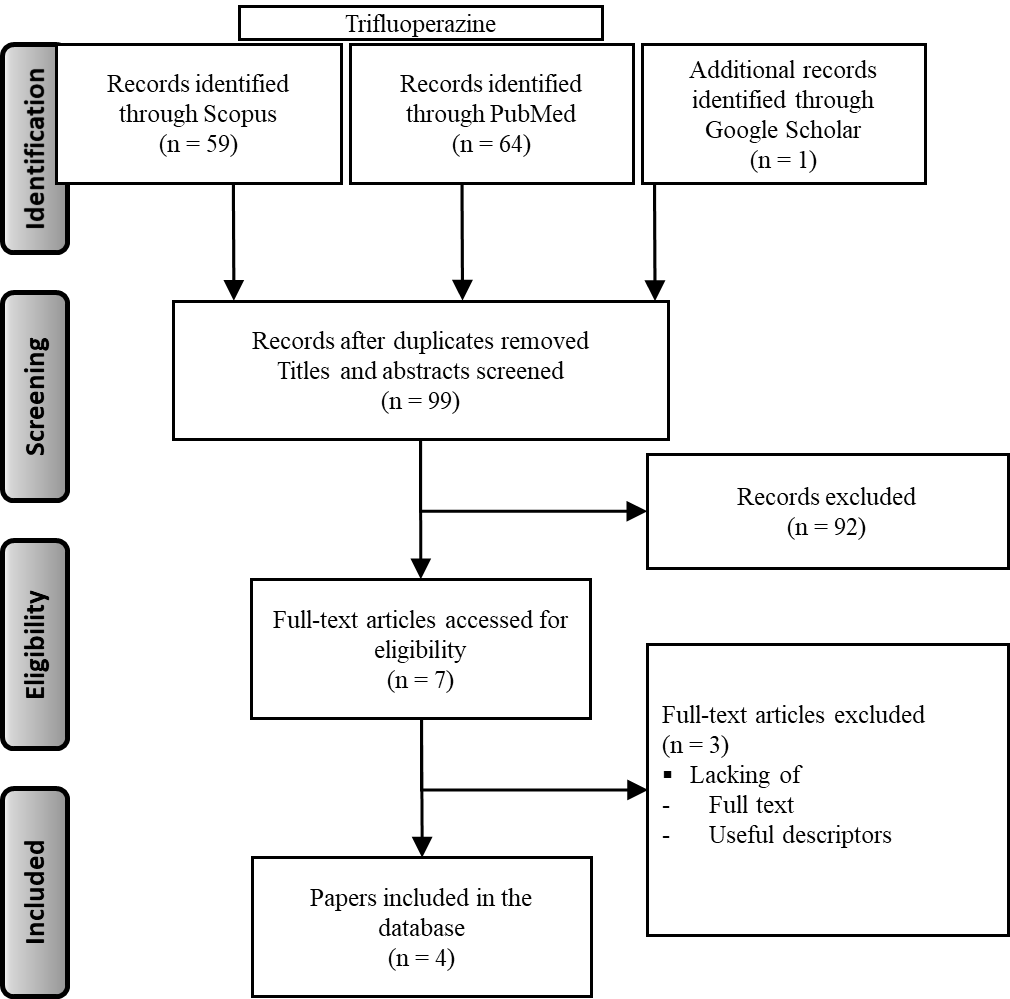

Supplement: Supplementary file 2 — Supplementary file2 (DOCX 1169 kb) [file 204_2020_2765_MOESM2_ESM.docx]
