# Supplementary material for: Human variability in isoform-specific UDP-glucuronosyltransferases: markers of acute and chronic exposure, polymorphisms and uncertainty factors
Source: Arch Toxicol. 2020 May 15;94(8):2637–61. doi: 10.1007/s00204-020-02765-8 (PMC7395075; doi:10.1007/s00204-020-02765-8)
Supplement: Supplementary file 3 — Supplementary file3 (DOCX 19508 kb) [file 204_2020_2765_MOESM3_ESM.docx]

**Supplementary Material 3 –** *Pharmacokinetic data shown per geographical ancestry to check differences between world populations*


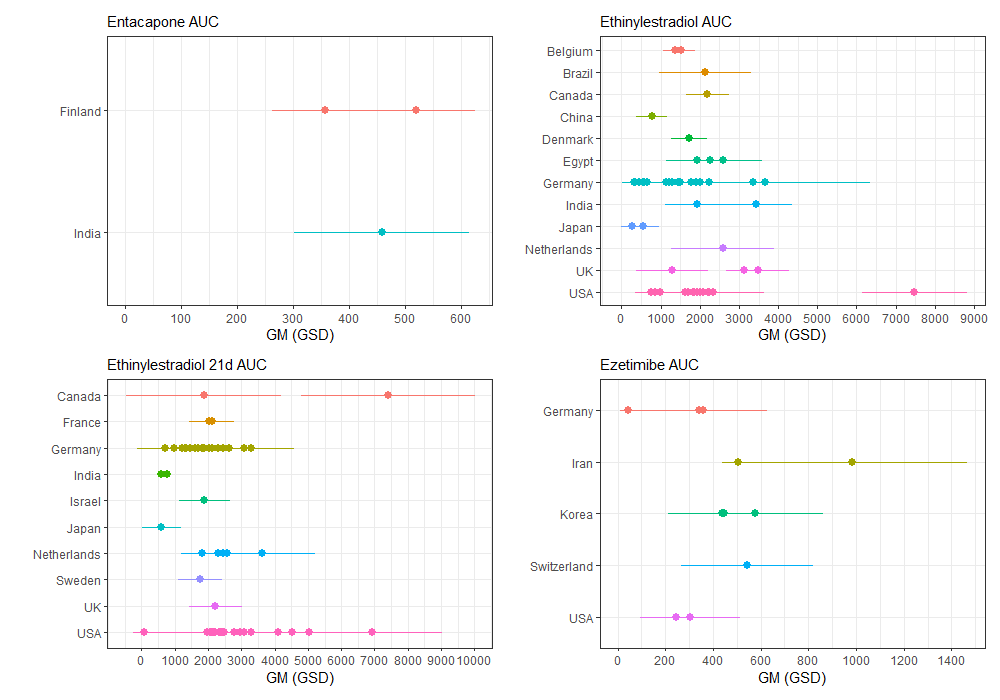


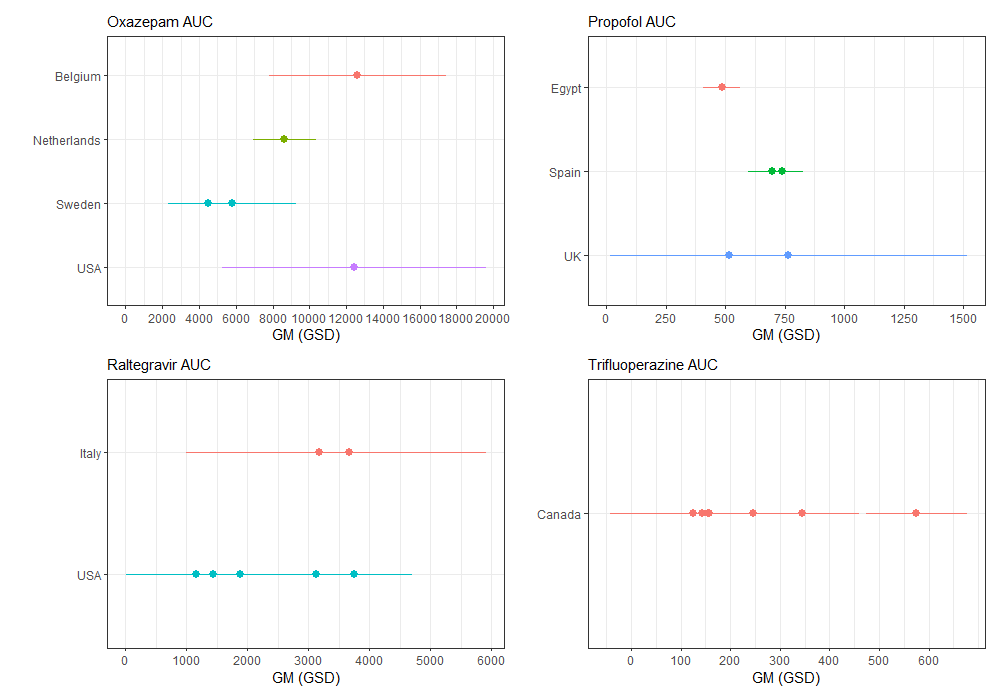


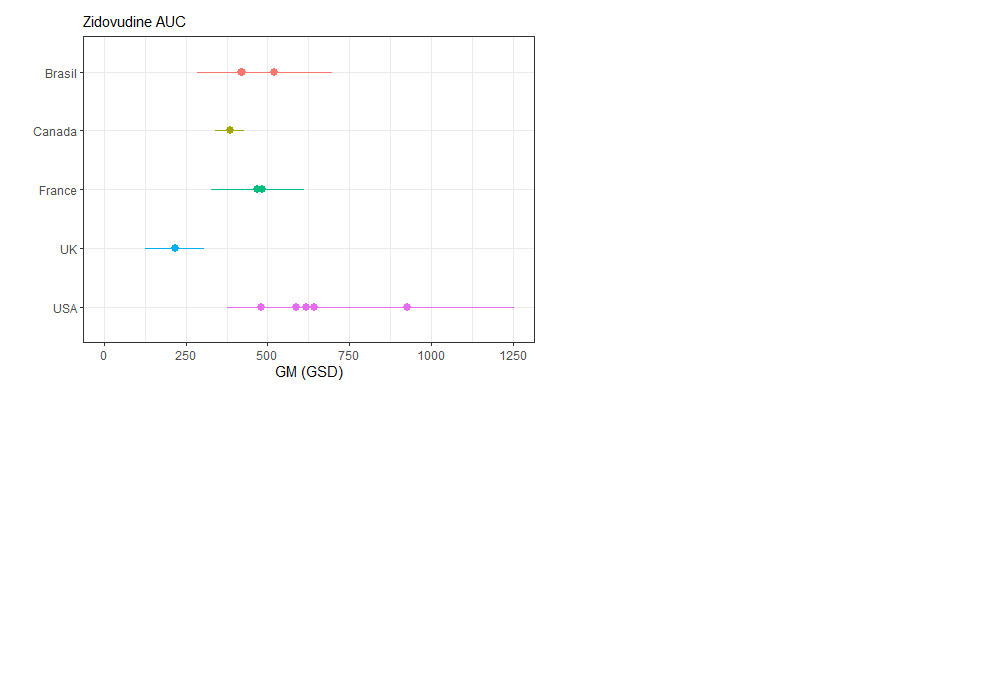


*Figure S*3.1 *Data on AUC (in ng*h/ml/dose) expressed as geometric mean (GM) and geometric standard deviation (GSD), split per country to check if there are any differences between countries that may point towards interethnic differences. Data after oral administration and intravenous administration are not distinguished here.*


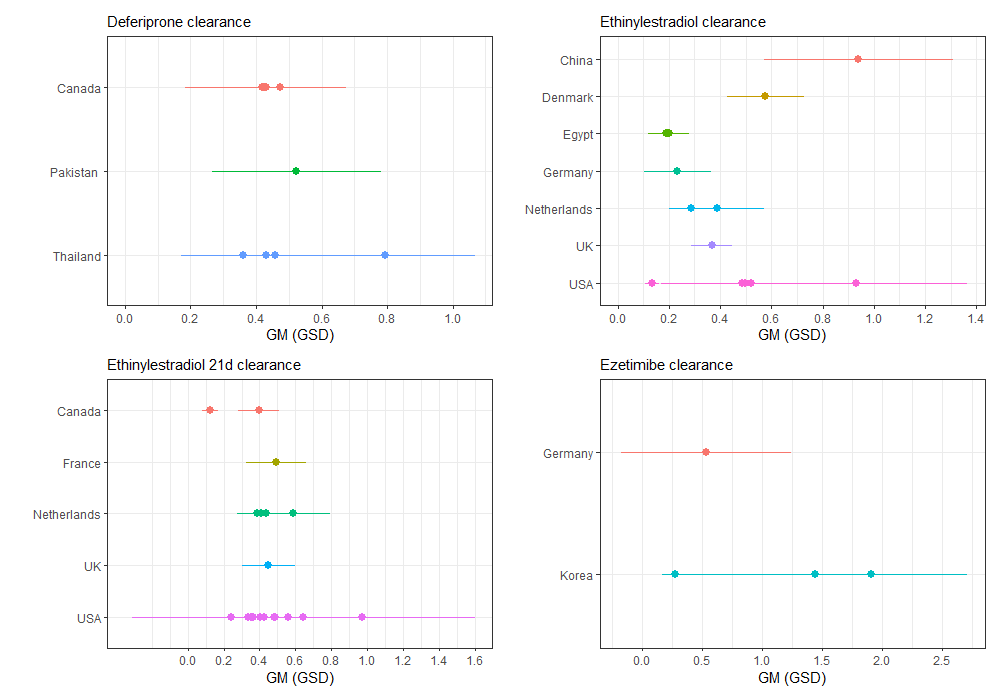

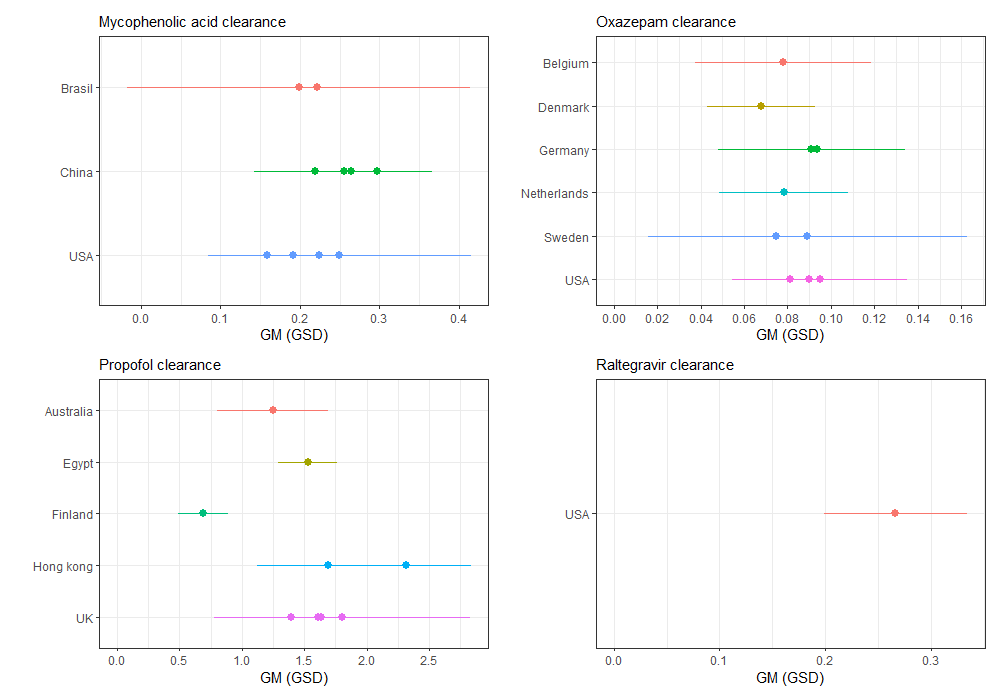

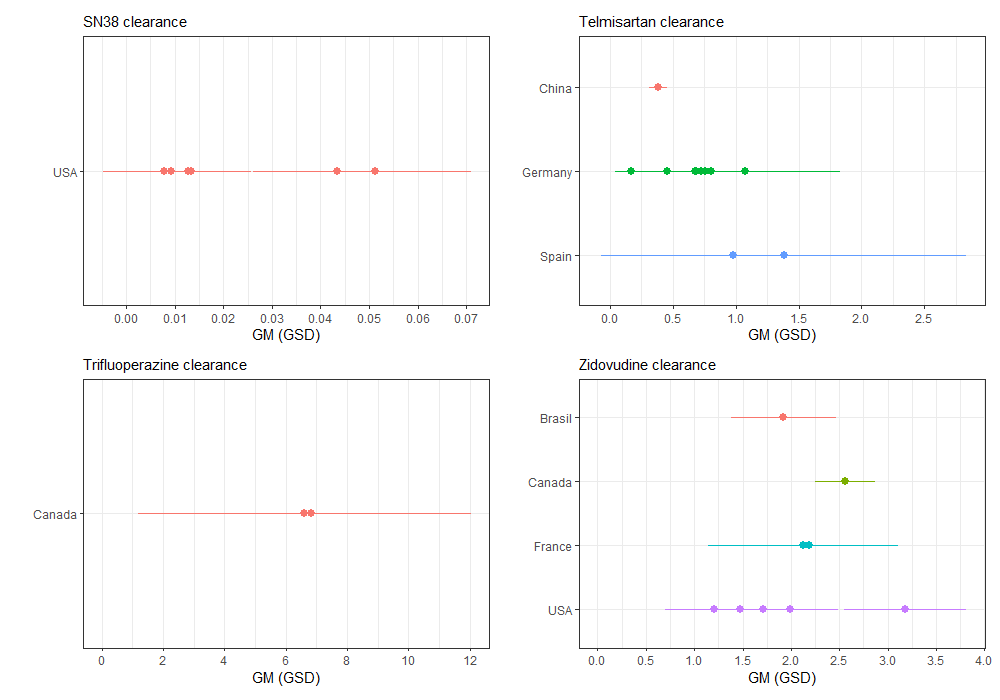


*Figure S*3.2 *Data on clearance (in L/h/kg) expressed as geometric mean (GM) and geometric standard deviation (GSD), split per country to check if there are any differences between countries that may point towards interethnic differences. Data after oral administration and intravenous administration are not distinguished here.*


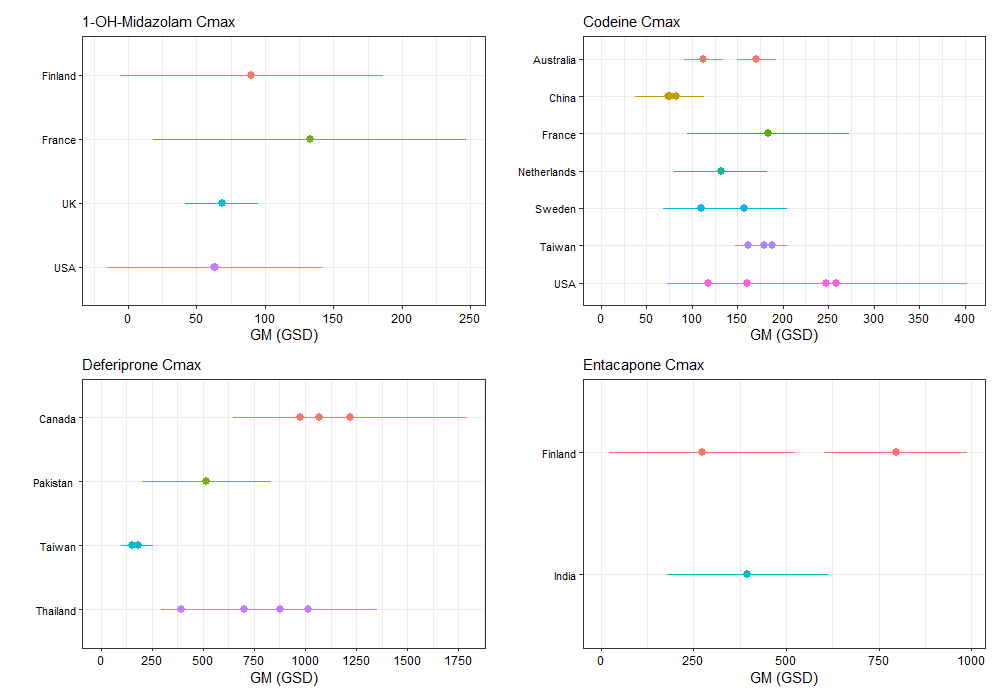


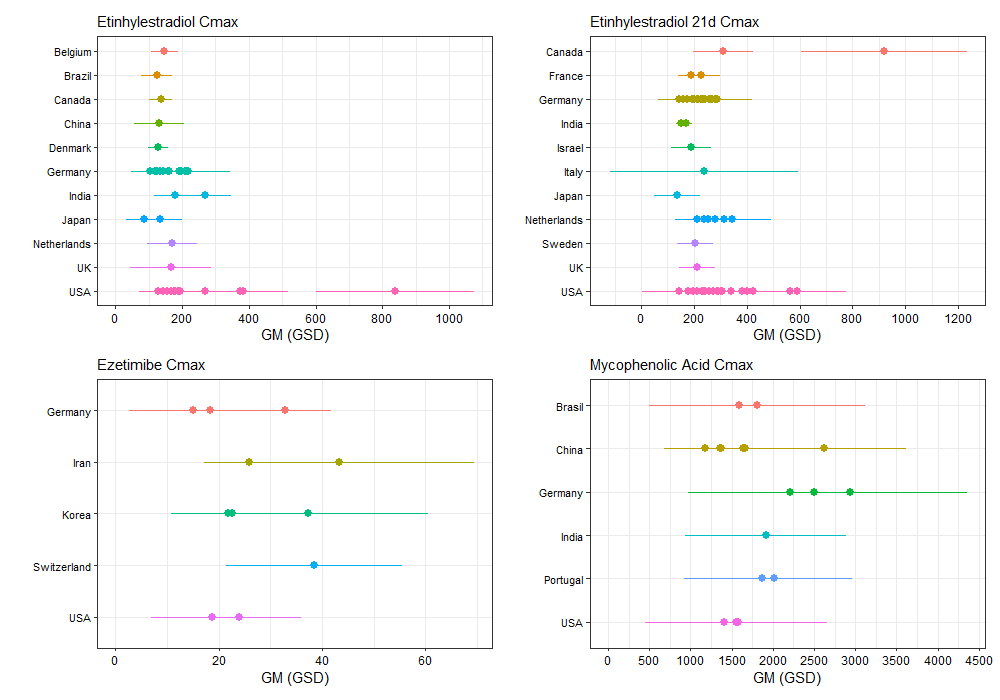


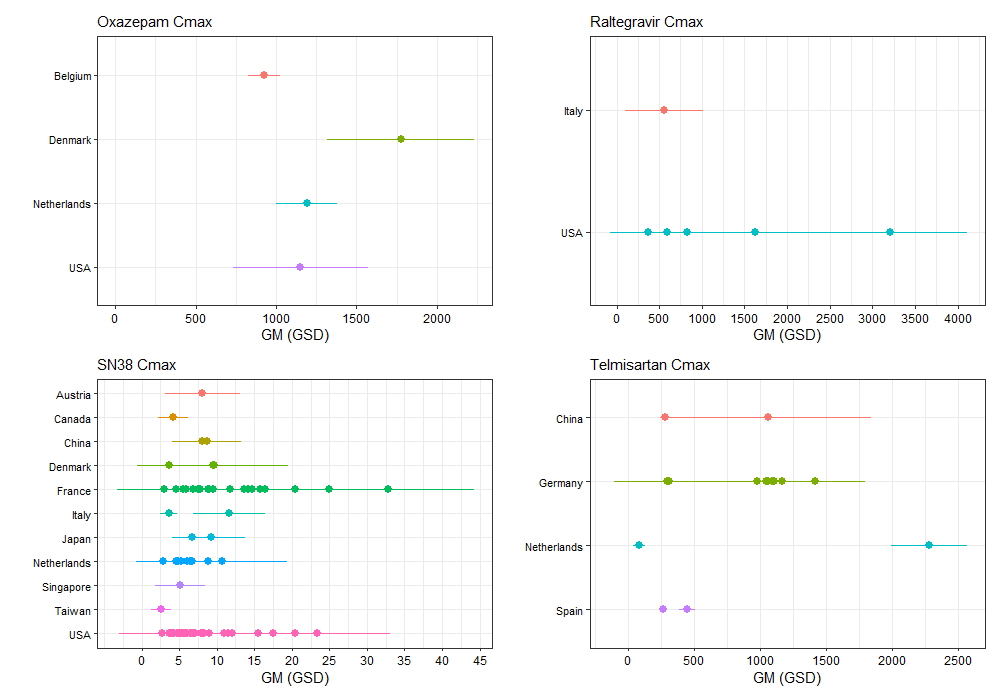


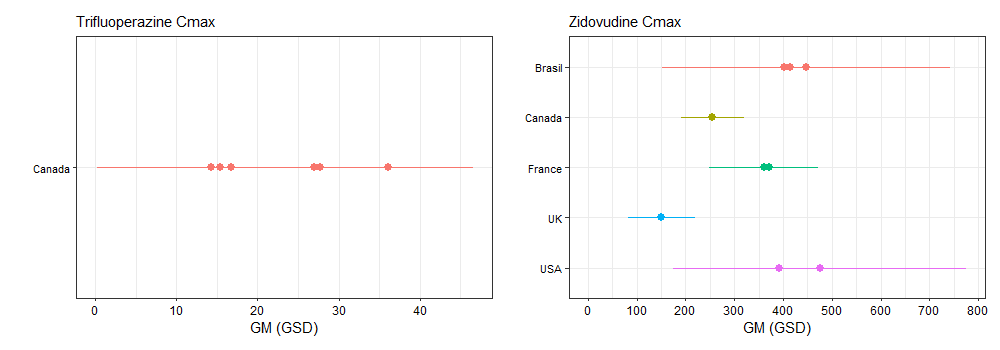


*Figure S*3.3 *Data on Cmax (in ng/ml/dose) expressed as geometric mean (GM) and geometric standard deviation (GSD), split per country to check if there are any differences between countries that may point towards interethnic differences. Data after oral administration and intravenous administration are not distinguished here.*
