# Supplementary material for: Human variability in isoform-specific UDP-glucuronosyltransferases: markers of acute and chronic exposure, polymorphisms and uncertainty factors
Source: Arch Toxicol. 2020 May 15;94(8):2637–61. doi: 10.1007/s00204-020-02765-8 (PMC7395075; doi:10.1007/s00204-020-02765-8)
Supplement: Supplementary file 4 — Supplementary file4 (DOCX 42 kb) [file 204_2020_2765_MOESM4_ESM.docx]

**Supplementary Material 4 -** *References Polymorphism Frequencies*

**1A1**

Alkharfy KM et al. (2013) Distribution of selected gene polymorphisms of UGT1A1 in a Saudi population Arch Med Sci 9:731-738 doi:10.5114/aoms.2013.37012

Arambula E, Vaca G (2002) Genotyping by"cold single-strand conformation polymorphism" of the UGT1A1 promoter polymorphism in Mexican mestizos Blood cells, molecules & diseases 28:86-90

Beutler E, Gelbart T, Demina A (1998) Racial variability in the UDP-glucuronosyltransferase 1 (UGT1A1) promoter: a balanced polymorphism for regulation of bilirubin metabolism? Proceedings of the National Academy of Sciences of the United States of America 95:8170-8174 doi:10.1073/pnas.95.14.8170

Borlak J, Thum T, Landt O, Erb K, Hermann R (2000) Molecular diagnosis of a familial nonhemolytic hyperbilirubinemia (Gilbert's syndrome) in healthy subjects Hepatology (Baltimore, Md) 32:792-795 doi:10.1053/jhep.2000.18193

Brureau L et al. (2016) Polymorphisms of Estrogen Metabolism-Related Genes and Prostate Cancer Risk in Two Populations of African Ancestry PLOS ONE 11:e0153609 doi:10.1371/journal.pone.0153609

Chaouch L et al. (2013) Early complication in Sickle Cell Anemia children due to A(TA)<formula>_n</formula> TAA polymorphism at the promoter of UGT1A1 gene Disease markers doi:10.3233/dma-130992

Horsfall LJ, Zeitlyn D, Tarekegn A, Bekele E, Thomas MG, Bradman N, Swallow DM (2011) Prevalence of clinically relevant UGT1A alleles and haplotypes in African populations Annals of human genetics 75:236-246 doi:10.1111/j.1469-1809.2010.00638.x

Jada SR et al. (2007) Role of UGT1A1*6, UGT1A1*28 and ABCG2 c.421C>A polymorphisms in irinotecan-induced neutropenia in Asian cancer patients Cancer science 98:1461-1467 doi:10.1111/j.1349-7006.2007.00541.x

Kobayashi M et al. (2012) Is there diversity among UGT1A1 polymorphism in Japan? World J Gastrointest Oncol 4:170-175 doi:10.4251/wjgo.v4.i7.170

Köhle C, Möhrle B, Münzel PA, Schwab M, Wernet D, Badary OA, Bock KW (2003) Frequent co-occurrence of the TATA box mutation associated with Gilbert’s syndrome (UGT1A1*28) with other polymorphisms of the UDP-glucuronosyltransferase-1 locus (UGT1A6*2 and UGT1A7*3) in Caucasians and Egyptians Biochemical Pharmacology 65:1521-1527 doi:https://doi.org/10.1016/S0006-2952(03)00074-1

Lampe JW, Bigler J, Horner NK, Potter JD (1999) UDP-glucuronosyltransferase (UGT1A1*28 and UGT1A6*2) polymorphisms in Caucasians and Asians: relationships to serum bilirubin concentrations Pharmacogenetics and genomics 9:341-350

Liu JY, Qu K, Sferruzza AD, Bender RA (2007) Distribution of the UGT1A1*28 polymorphism in Caucasian and Asian populations in the US: a genomic analysis of 138 healthy individuals Anti-cancer drugs 18:693-696 doi:10.1097/CAD.0b013e32803a46fe

Pacheco PR et al. (2009) UGT1A1, UGT1A6 and UGT1A7 genetic analysis: repercussion for irinotecan pharmacogenetics in the Sao Miguel Island Population (Azores, Portugal) Molecular diagnosis & therapy 13:261-268 doi:10.2165/11317170-000000000-00000

Park WB et al. (2010) Genetic factors influencing severe atazanavir-associated hyperbilirubinemia in a population with low UDP-glucuronosyltransferase 1A1*28 allele frequency Clinical infectious diseases : an official publication of the Infectious Diseases Society of America 51:101-106 doi:10.1086/653427

Premawardhena A et al. (2003) The global distribution of length polymorphisms of the promoters of the glucuronosyltransferase 1 gene (UGT1A1): hematologic and evolutionary implications Blood cells, molecules & diseases 31:98-101

Te Morsche RH, Zusterzeel PL, Raijmakers MT, Roes EM, Steegers EA, Peters WH (2001) Polymorphism in the promoter region of the bilirubin UDP-glucuronosyltransferase (Gilbert's syndrome) in healthy Dutch subjects Hepatology (Baltimore, Md) 33:765 doi:10.1053/jhep.2001.0103303le03

Teh LK, Hashim H, Zakaria ZA, Salleh MZ (2012) Polymorphisms of UGT1A1*6, UGT1A1*27 & UGT1A1*28 in three major ethnic groups from Malaysia The Indian journal of medical research 136:249-259

Turatti L et al. (2012) Short communication: UGT1A1*28 variant allele is a predictor of severe hyperbilirubinemia in HIV-infected patients on HAART in southern Brazil AIDS research and human retroviruses 28:1015-1018 doi:10.1089/aid.2011.0261

**1A3**

Caillier B et al. (2007) A pharmacogenomics study of the human estrogen glucuronosyltransferase UGT1A3 Pharmacogenetics and genomics 17:481-495 doi:10.1097/FPC.0b013e32806d87a4

Chen Y, Chen S, Li X, Wang X, Zeng S (2006) Genetic variants of human UGT1A3: functional characterization and frequency distribution in a Chinese Han population Drug metabolism and disposition: the biological fate of chemicals 34:1462-1467 doi:10.1124/dmd.106.009761

Cho SK, Oh ES, Park K, Park MS, Chung JY (2012) The UGT1A3*2 polymorphism affects atorvastatin lactonization and lipid-lowering effect in healthy volunteers Pharmacogenetics and genomics 22:598-605 doi:10.1097/FPC.0b013e3283544085

Iwai M, Maruo Y, Ito M, Yamamoto K, Sato H, Takeuchi Y (2004) Six novel UDP-glucuronosyltransferase (UGT1A3) polymorphisms with varying activity Journal of human genetics 49:123-128 doi:10.1007/s10038-003-0119-y

Lin M et al. (2017) Effects of UDP-glucuronosyltransferase (UGT) polymorphisms on the pharmacokinetics of febuxostat in healthy Chinese volunteers Drug metabolism and pharmacokinetics 32:77-84 doi:10.1016/j.dmpk.2016.08.003

Riedmaier S et al. (2010) UDP-glucuronosyltransferase (UGT) polymorphisms affect atorvastatin lactonization in vitro and in vivo Clinical pharmacology and therapeutics 87:65-73 doi:10.1038/clpt.2009.181

**1A4**

Chang Y, Yang L-y, Zhang M-c, Liu S-Y (2014) Correlation of the UGT1A4 gene polymorphism with serum concentration and therapeutic efficacy of lamotrigine in Han Chinese of Northern China European Journal of Clinical Pharmacology 70:941-946 doi:10.1007/s00228-014-1690-1

Ehmer U, Vogel A, Schutte JK, Krone B, Manns MP, Strassburg CP (2004) Variation of hepatic glucuronidation: Novel functional polymorphisms of the UDP-glucuronosyltransferase UGT1A4 Hepatology (Baltimore, Md) 39:970-977 doi:10.1002/hep.20131

Gaibar M, Novillo A, Romero-Lorca A, Esteban ME, Fernández-Santander A (2018) Pharmacogenetics of ugt genes in North African populations The pharmacogenomics journal 18:609-612 doi:10.1038/s41397-018-0034-4

Ghotbi R, Mannheimer B, Aklillu E, Suda A, Bertilsson L, Eliasson E, Osby U (2010) Carriers of the UGT1A4 142T>G gene variant are predisposed to reduced olanzapine exposure--an impact similar to male gender or smoking in schizophrenic patients Eur J Clin Pharmacol 66:465-474 doi:10.1007/s00228-009-0783-8

Gulcebi MI, Ozkaynakci A, Goren MZ, Aker RG, Ozkara C, Onat FY (2011) The relationship between UGT1A4 polymorphism and serum concentration of lamotrigine in patients with epilepsy Epilepsy research 95:1-8 doi:10.1016/j.eplepsyres.2011.01.016

Hakooz N, Alzubiedi S, Yousef AM, Arafat T, Dajani R, Ababneh N, Ismail S (2012) UDP-glucuronosyltransferase 1A4 (UGT1A4) polymorphisms in a Jordanian population Molecular biology reports 39:7763-7768 doi:10.1007/s11033-012-1615-y

Kim DW, Kim M, Lee SK, Kang R, Lee SY (2006) Lack of association between L48V polymorphism in the UGT1A4 gene and lamotrigine-induced rash J Korean Epilepsy Soc 10:31-34

Lopez M et al. (2013) Interethnic differences in UGT1A4 genetic polymorphisms between Mexican Mestizo and Spanish populations Molecular biology reports 40:3187-3192 doi:10.1007/s11033-012-2393-2

Menard V, Girard H, Harvey M, Perusse L, Guillemette C (2009) Analysis of inherited genetic variations at the UGT1 locus in the French-Canadian population Human mutation 30:677-687 doi:10.1002/humu.20946

Mori A, Maruo Y, Iwai M, Sato H, Takeuchi Y (2005) UDP-glucuronosyltransferase 1A4 polymorphisms in a Japanese population and kinetics of clozapine glucuronidation Drug metabolism and disposition: the biological fate of chemicals 33:672-675 doi:10.1124/dmd.104.002576

Reimers A, Sjursen W, Helde G, Brodtkorb E (2016) Frequencies of UGT1A4*2 (P24T) and *3 (L48V) and their effects on serum concentrations of lamotrigine European journal of drug metabolism and pharmacokinetics 41:149-155 doi:10.1007/s13318-014-0247-0

Romero-Lorca A, Novillo A, Gaibar M, Bandres F, Fernandez-Santander A (2015) Impacts of the Glucuronidase Genotypes UGT1A4, UGT2B7, UGT2B15 and UGT2B17 on Tamoxifen Metabolism in Breast Cancer Patients PLoS One 10:e0132269 doi:10.1371/journal.pone.0132269

Saeki M et al. (2005) Genetic Variations and Haplotypes of UGT1A4 in a Japanese Population Drug metabolism and pharmacokinetics 20:144-151 doi:https://doi.org/10.2133/dmpk.20.144

Shirzadi M, Reimers A, Helde G, Sjursen W, Brodtkorb E (2017) No association between non-bullous skin reactions from lamotrigine and heterozygosity of UGT1A4 genetic variants *2(P24T) or *3(L48V) in Norwegian patients Seizure 45:169-171 doi:10.1016/j.seizure.2016.12.015

Suh HJ et al. (2018) The Genetic Polymorphism UGT1A4*3 Is Associated with Low Posaconazole Plasma Concentrations in Hematological Malignancy Patients Receiving the Oral Suspension Antimicrobial agents and chemotherapy 62 doi:10.1128/aac.02230-17

Sutiman N et al. (2016) Pharmacogenetics of UGT1A4, UGT2B7 and UGT2B15 and Their Influence on Tamoxifen Disposition in Asian Breast Cancer Patients Clinical Pharmacokinetics 55:1239-1250 doi:10.1007/s40262-016-0402-7

**1A6**

Alkharfy KM et al. (2017) Prevalence of UDP-glucuronosyltransferase polymorphisms (UGT1A6 *2, 1A7 *12, 1A8 *3, 1A9 *3, 2B7 *2, and 2B15 *2) in a Saudi population Saudi pharmaceutical journal : SPJ : the official publication of the Saudi Pharmaceutical Society 25:224-230 doi:10.1016/j.jsps.2016.05.009

Köhle C, Möhrle B, Münzel PA, Schwab M, Wernet D, Badary OA, Bock KW (2003) Frequent co-occurrence of the TATA box mutation associated with Gilbert’s syndrome (UGT1A1*28) with other polymorphisms of the UDP-glucuronosyltransferase-1 locus (UGT1A6*2 and UGT1A7*3) in Caucasians and Egyptians Biochemical Pharmacology 65:1521-1527 doi:https://doi.org/10.1016/S0006-2952(03)00074-1

Lampe JW, Bigler J, Horner NK, Potter JD (1999) UDP-glucuronosyltransferase (UGT1A1*28 and UGT1A6*2) polymorphisms in Caucasians and Asians: relationships to serum bilirubin concentrations Pharmacogenetics and genomics 9:341-350

Limenta LMG et al. (2008) UGT1A6 genotype-related pharmacokinetics of deferiprone (L1) in healthy volunteers British journal of clinical pharmacology 65:908-916 doi:10.1111/j.1365-2125.2008.03103.x

McGreavey LE et al. (2005) No evidence that polymorphisms in CYP2C8, CYP2C9, UGT1A6, PPARδ and PPARγ act as modifiers of the protective effect of regular NSAID use on the risk of colorectal carcinoma Pharmacogenetics and genomics 15:713-721 doi:10.1097/01.fpc.0000174786.85238.63

Navarro SL et al. (2011) <em>UGT1A6</em> and <em>UGT2B15</em> Polymorphisms and Acetaminophen Conjugation in Response to a Randomized, Controlled Diet of Select Fruits and Vegetables Drug Metabolism and Disposition 39:1650-1657 doi:10.1124/dmd.111.039149

Pacheco PR et al. (2009) UGT1A1, UGT1A6 and UGT1A7 genetic analysis: repercussion for irinotecan pharmacogenetics in the Sao Miguel Island Population (Azores, Portugal) Molecular diagnosis & therapy 13:261-268 doi:10.2165/11317170-000000000-00000

Saeki M et al. (2005) Genetic polymorphisms of UGT1A6 in a Japanese population Drug metabolism and pharmacokinetics 20:85-90

**1A9**

Alkharfy KM et al. (2017) Prevalence of UDP-glucuronosyltransferase polymorphisms (UGT1A6 *2, 1A7 *12, 1A8 *3, 1A9 *3, 2B7 *2, and 2B15 *2) in a Saudi population Saudi pharmaceutical journal : SPJ : the official publication of the Saudi Pharmaceutical Society 25:224-230 doi:10.1016/j.jsps.2016.05.009

Carlini LE et al. (2005) UGT1A7 and UGT1A9 polymorphisms predict response and toxicity in colorectal cancer patients treated with capecitabine/irinotecan Clinical cancer research : an official journal of the American Association for Cancer Research 11:1226-1236

Cecchin E et al. (2009) Predictive role of the UGT1A1, UGT1A7, and UGT1A9 genetic variants and their haplotypes on the outcome of metastatic colorectal cancer patients treated with fluorouracil, leucovorin, and irinotecan Journal of clinical oncology : official journal of the American Society of Clinical Oncology 27:2457-2465 doi:10.1200/jco.2008.19.0314

Fujita K et al. (2007) Genetic linkage of UGT1A7 and UGT1A9 polymorphisms to UGT1A1*6 is associated with reduced activity for SN-38 in Japanese patients with cancer Cancer chemotherapy and pharmacology 60:515-522 doi:10.1007/s00280-006-0396-1

Girard H, Butler LM, Villeneuve L, Millikan RC, Sinha R, Sandler RS, Guillemette C (2008) UGT1A1 and UGT1A9 functional variants, meat intake, and colon cancer, among Caucasians and African-Americans Mutation research 644:56-63 doi:10.1016/j.mrfmmm.2008.07.002

Girard H et al. (2004) Identification of common polymorphisms in the promoter of the UGT1A9 gene: evidence that UGT1A9 protein and activity levels are strongly genetically controlled in the liver Pharmacogenetics 14:501-515

Innocenti F, Liu W, Chen P, Desai AA, Das S, Ratain MJ (2005) Haplotypes of variants in the UDP-glucuronosyltransferase1A9 and 1A1 genes Pharmacogenetics and genomics 15:295-301

Jiao Z et al. (2008) Population pharmacokinetic modelling for enterohepatic circulation of mycophenolic acid in healthy Chinese and the influence of polymorphisms in UGT1A9 British journal of clinical pharmacology 65:893-907 doi:10.1111/j.1365-2125.2008.03109.x

Korprasertthaworn P, Udomuksorn W, Yoovathaworn K (2009) Three Novel Single Nucleotide Polymorphisms of UGT1A9 in a Thai Population Drug metabolism and pharmacokinetics 24:482-485 doi:https://doi.org/10.2133/dmpk.24.482

Kuypers DR, Naesens M, Vermeire S, Vanrenterghem Y (2005) The impact of uridine diphosphate-glucuronosyltransferase 1A9 (UGT1A9) gene promoter region single-nucleotide polymorphisms T-275A and C-2152T on early mycophenolic acid dose-interval exposure in de novo renal allograft recipients Clinical pharmacology and therapeutics 78:351-361 doi:10.1016/j.clpt.2005.06.007

Maeda H et al. (2014) Differences in UGT1A1, UGT1A7, and UGT1A9 polymorphisms between Uzbek and Japanese populations Molecular diagnosis & therapy 18:333-342 doi:10.1007/s40291-014-0083-6

Mazidi T et al. (2013) Impact of UGT1A9 Polymorphism on Mycophenolic Acid Pharmacokinetic Parameters in Stable Renal Transplant Patients Iran J Pharm Res 12:547-556

Mehlotra RK, Bockarie MJ, Zimmerman PA (2007) Prevalence of UGT1A9 and UGT2B7 nonsynonymous single nucleotide polymorphisms in West African, Papua New Guinean, and North American populations Eur J Clin Pharmacol 63:1-8 doi:10.1007/s00228-006-0206-z

Menard V, Girard H, Harvey M, Perusse L, Guillemette C (2009) Analysis of inherited genetic variations at the UGT1 locus in the French-Canadian population Human mutation 30:677-687 doi:10.1002/humu.20946

Paoluzzi L et al. (2004) Influence of genetic variants in UGT1A1 and UGT1A9 on the in vivo glucuronidation of SN-38 Journal of clinical pharmacology 44:854-860 doi:10.1177/0091270004267159

Saeki M et al. (2006) Haplotype structures of the UGT1A gene complex in a Japanese population The pharmacogenomics journal 6:63-75 doi:10.1038/sj.tpj.6500335

Villeneuve L, Girard H, Fortier LC, Gagne JF, Guillemette C (2003) Novel functional polymorphisms in the UGT1A7 and UGT1A9 glucuronidating enzymes in Caucasian and African-American subjects and their impact on the metabolism of 7-ethyl-10-hydroxycamptothecin and flavopiridol anticancer drugs The Journal of pharmacology and experimental therapeutics 307:117-128 doi:10.1124/jpet.103.054072

Wang Y et al. (2012) Variants, haplotypes and htSNPs of UDP-glucuronosyltransferase 1A9, 1A7 and 1A1 genes in Chinese Tibetan Population vol 18.

Yamanaka H et al. (2004) A novel polymorphism in the promoter region of human UGT1A9 gene (UGT1A9*22) and its effects on the transcriptional activity Pharmacogenetics 14:329-332

Zakerska O, Skrzypczak-Zielinska M, Mikstacki A, Tamowicz B, Malengowska B, Szalata M, Slomski R (2013) Genotype and allele frequencies of polymorphic UGT1A9 in the Polish population European journal of drug metabolism and pharmacokinetics 38:217-221 doi:10.1007/s13318-012-0110-0

Zhang X et al. (2012) Genetic variants and haplotypes of the UGT1A9, 1A7 and 1A1 genes in Chinese Han Genet Mol Biol 35:428-434 doi:10.1590/S1415-47572012005000036

**2B7**

Alkharfy KM et al. (2017) Prevalence of UDP-glucuronosyltransferase polymorphisms (UGT1A6 *2, 1A7 *12, 1A8 *3, 1A9 *3, 2B7 *2, and 2B15 *2) in a Saudi population Saudi pharmaceutical journal : SPJ : the official publication of the Saudi Pharmaceutical Society 25:224-230 doi:10.1016/j.jsps.2016.05.009

Bhasker CR, McKinnon W, Stone A, Lo AC, Kubota T, Ishizaki T, Miners JO (2000) Genetic polymorphism of UDP-glucuronosyltransferase 2B7 (UGT2B7) at amino acid 268: ethnic diversity of alleles and potential clinical significance Pharmacogenetics 10:679-685

Chung CJ et al. (2013) 4-(Methylnitrosamino)-1-(3-pyridyl)-1-butanone (NNK) metabolism-related enzymes gene polymorphisms, NNK metabolites levels and urothelial carcinoma Toxicology letters 216:16-22 doi:10.1016/j.toxlet.2012.11.002

Court MH, Krishnaswamy S, Hao Q, Duan SX, Patten CJ, Von Moltke LL, Greenblatt DJ (2003) Evaluation of 3'-azido-3'-deoxythymidine, morphine, and codeine as probe substrates for UDP-glucuronosyltransferase 2B7 (UGT2B7) in human liver microsomes: specificity and influence of the UGT2B7*2 polymorphism Drug metabolism and disposition: the biological fate of chemicals 31:1125-1133 doi:10.1124/dmd.31.9.1125

Daly AK, Aithal GP, Leathart JB, Swainsbury RA, Dang TS, Day CP (2007) Genetic susceptibility to diclofenac-induced hepatotoxicity: contribution of UGT2B7, CYP2C8, and ABCC2 genotypes Gastroenterology 132:272-281 doi:10.1053/j.gastro.2006.11.023

Deng XY, Wang CX, Wang XD, Bi HC, Chen X, Li JL, Huang M (2013) Genetic polymorphisms of UGT1A8, UGT1A9, UGT2B7 and ABCC2 in Chinese renal transplant recipients and a comparison with other ethnic populations Die Pharmazie 68:240-244

Gaibar M, Novillo A, Romero-Lorca A, Esteban ME, Fernandez-Santander A (2018) Pharmacogenetics of ugt genes in North African populations The pharmacogenomics journal 18:609-612 doi:10.1038/s41397-018-0034-4

Hwang MS, Lee SJ, Jeong HE, Lee S, Yoo MA, Shin JG (2010) Genetic variations in UDP-glucuronosyltransferase 2B7 gene (UGT2B7) in a Korean population Drug metabolism and pharmacokinetics 25:398-402

Jarrar Y, F K, A O, Hamadneh L, Albawab aq (2016) The Frequency of UGT2B7*2 (802C>T) Allele among Healthy Unrelated Jordanian Volunteers vol 7. doi:10.4172/2157-7609.1000218

Kelly LE et al. (2013) A clinical tool for reducing central nervous system depression among neonates exposed to codeine through breast milk PLoS One 8:e70073 doi:10.1371/journal.pone.0070073

Lampe JW, Bigler J, Bush AC, Potter JD (2000) Prevalence of Polymorphisms in the Human UDP-Glucuronosyltransferase 2B Family: <em>UGT2B4(D</em><sup>458</sup><em>E)</em>, <em>UGT2B7(H</em><sup>268</sup><em>Y)</em>, and <em>UGT2B15(D</em><sup>85</sup><em>Y)</em> Cancer Epidemiology Biomarkers &amp; Prevention 9:329-333

Levesque E, Delage R, Benoit-Biancamano MO, Caron P, Bernard O, Couture F, Guillemette C (2007) The impact of UGT1A8, UGT1A9, and UGT2B7 genetic polymorphisms on the pharmacokinetic profile of mycophenolic acid after a single oral dose in healthy volunteers Clinical pharmacology and therapeutics 81:392-400 doi:10.1038/sj.clpt.6100073

Lin GF et al. (2005) An association of UDP-glucuronosyltransferase 2B7 C802T (His268Tyr) polymorphism with bladder cancer in benzidine-exposed workers in China Toxicological sciences : an official journal of the Society of Toxicology 85:502-506 doi:10.1093/toxsci/kfi068

Roco A et al. (2012) Frequencies of 23 functionally significant variant alleles related with metabolism of antineoplastic drugs in the chilean population: comparison with caucasian and asian populations Frontiers in genetics 3:229 doi:10.3389/fgene.2012.00229

Romero-Lorca A, Novillo A, Gaibar M, Bandres F, Fernandez-Santander A (2015) Impacts of the Glucuronidase Genotypes UGT1A4, UGT2B7, UGT2B15 and UGT2B17 on Tamoxifen Metabolism in Breast Cancer Patients PLoS One 10:e0132269 doi:10.1371/journal.pone.0132269

Sastre JA, Varela G, Lopez M, Muriel C, Gonzalez-Sarmiento R (2015) Influence of uridine diphosphate-glucuronyltransferase 2B7 (UGT2B7) variants on postoperative buprenorphine analgesia Pain practice : the official journal of World Institute of Pain 15:22-30 doi:10.1111/papr.12152

Sparks R et al. (2004) UDP-glucuronosyltransferase and sulfotransferase polymorphisms, sex hormone concentrations, and tumor receptor status in breast cancer patients Breast Cancer Research 6:R488 doi:10.1186/bcr818

Sutiman N et al. (2016) Pharmacogenetics of UGT1A4, UGT2B7 and UGT2B15 and Their Influence on Tamoxifen Disposition in Asian Breast Cancer Patients Clinical Pharmacokinetics 55:1239-1250 doi:10.1007/s40262-016-0402-7

**2B15**

Alkharfy KM et al. (2017) Prevalence of UDP-glucuronosyltransferase polymorphisms (UGT1A6 *2, 1A7 *12, 1A8 *3, 1A9 *3, 2B7 *2, and 2B15 *2) in a Saudi population Saudi pharmaceutical journal : SPJ : the official publication of the Saudi Pharmaceutical Society 25:224-230 doi:10.1016/j.jsps.2016.05.009

Court MH, Hao Q, Krishnaswamy S, Bekaii-Saab T, Al-Rohaimi A, von Moltke LL, Greenblatt DJ (2004) UDP-Glucuronosyltransferase (UGT) 2B15 Pharmacogenetics: UGT2B15 D85Y Genotype and Gender Are Major Determinants of Oxazepam Glucuronidation by Human Liver Journal of Pharmacology and Experimental Therapeutics 310:656-665 doi:10.1124/jpet.104.067660

Gsur A et al. (2002) A Polymorphism in the <strong><em>UDP-Glucuronosyltransferase 2B15</em></strong> Gene (D<sup>85</sup>Y) Is Not Associated with Prostate Cancer Risk Cancer Epidemiology Biomarkers &amp; Prevention 11:497-498

Habibi M, Mirfakhraie R, Khani M, Rakhshan A, Azargashb E, Pouresmaeili F (2017) Genetic variations in UGT2B28, UGT2B17, UGT2B15 genes and the risk of prostate cancer: A case-control study Gene 634:47-52 doi:https://doi.org/10.1016/j.gene.2017.08.038

Hajdinjak T, Zagradisnik B (2004) Prostate cancer and polymorphism D85Y in gene for dihydrotestosterone degrading enzyme UGT2B15: Frequency of DD homozygotes increases with Gleason Score The Prostate 59:436-439 doi:10.1002/pros.20024

He X, Hesse LM, Hazarika S, Masse G, Harmatz JS, Greenblatt DJ, Court MH (2009) Evidence for oxazepam as an in vivo probe of UGT2B15: oxazepam clearance is reduced by UGT2B15 D85Y polymorphism but unaffected by UGT2B17 deletion British journal of clinical pharmacology 68:721-730 doi:10.1111/j.1365-2125.2009.03519.x

Hwang M-S, Lee S-J, Kim W-Y, Jeong H-E, Shin J-G (2014) Genetic Variations in UDP-glucuronosyltransferase 2B15 in a Korean Population Drug metabolism and pharmacokinetics 29:105-109 doi:https://doi.org/10.2133/dmpk.DMPK-13-SC-054

Lampe JW, Bigler J, Bush AC, Potter JD (2000) Prevalence of Polymorphisms in the Human UDP-Glucuronosyltransferase 2B Family: <em>UGT2B4(D</em><sup>458</sup><em>E)</em>, <em>UGT2B7(H</em><sup>268</sup><em>Y)</em>, and <em>UGT2B15(D</em><sup>85</sup><em>Y)</em> Cancer Epidemiology Biomarkers &amp; Prevention 9:329-333

MacLeod SL, Nowell S, Plaxco J, Lang NP (2000) An Allele-Specific Polymerase Chain Reaction Method for the Determination of the D85Y Polymorphism in the Human UDPGlucuronosyltransferase 2B15 Gene in a Case-Control Study of Prostate Cancer Annals of Surgical Oncology 7:777-782 doi:10.1007/s10434-000-0777-3

Menard V, Eap O, Harvey M, Guillemette C, Levesque E (2009) Copy-number variations (CNVs) of the human sex steroid metabolizing genes UGT2B17 and UGT2B28 and their associations with a UGT2B15 functional polymorphism Human mutation 30:1310-1319 doi:10.1002/humu.21054

Mijderwijk H, Klimek M, van Beek S, van Schaik RHN, Duivenvoorden HJ, Stolker RJ (2016) Implication of UGT2B15 Genotype Polymorphism on Postoperative Anxiety Levels in Patients Receiving Lorazepam Premedication Anesthesia & Analgesia 123:1109-1115 doi:10.1213/ane.0000000000001508

Navarro SL et al. (2011) <em>UGT1A6</em> and <em>UGT2B15</em> Polymorphisms and Acetaminophen Conjugation in Response to a Randomized, Controlled Diet of Select Fruits and Vegetables Drug Metabolism and Disposition 39:1650-1657 doi:10.1124/dmd.111.039149

Okugi H, Nakazato H, Matsui H, Ohtake N, Nakata S, Suzuki K (2006) Association of the polymorphisms of genes involved in androgen metabolism and signaling pathways with familial prostate cancer risk in a Japanese population Cancer Detection and Prevention 30:262-268 doi:https://doi.org/10.1016/j.cdp.2006.04.004

Riedy M, Wang J-Y, Miller AP, Buckler A, Hall J, Guida M (2000) Genomic organization of the UGT2b gene cluster on human chromosome 4q13 Pharmacogenetics and genomics 10:251-260

Romero-Lorca A, Novillo A, Gaibar M, Bandres F, Fernandez-Santander A (2015) Impacts of the Glucuronidase Genotypes UGT1A4, UGT2B7, UGT2B15 and UGT2B17 on Tamoxifen Metabolism in Breast Cancer Patients PLoS One 10:e0132269 doi:10.1371/journal.pone.0132269

Sparks R et al. (2004) UDP-glucuronosyltransferase and sulfotransferase polymorphisms, sex hormone concentrations, and tumor receptor status in breast cancer patients Breast Cancer Research 6:R488 doi:10.1186/bcr818

Stringer F, Scott G, Valbuena M, Kinley J, Nishihara M, Urquhart R (2013) The effect of genetic polymorphisms in UGT2B15 on the pharmacokinetic profile of sipoglitazar, a novel anti-diabetic agent European Journal of Clinical Pharmacology 69:423-430 doi:10.1007/s00228-012-1382-7

Sutiman N et al. (2016) Pharmacogenetics of UGT1A4, UGT2B7 and UGT2B15 and Their Influence on Tamoxifen Disposition in Asian Breast Cancer Patients Clinical Pharmacokinetics 55:1239-1250 doi:10.1007/s40262-016-0402-7

Swanson C et al. (2007) The Uridine Diphosphate Glucuronosyltransferase 2B15 D85Y and 2B17 Deletion Polymorphisms Predict the Glucuronidation Pattern of Androgens and Fat Mass in Men The Journal of Clinical Endocrinology & Metabolism 92:4878-4882 doi:10.1210/jc.2007-0359

Wegman P, Elingarami S, Carstensen J, Stål O, Nordenskjöld B, Wingren S (2007) Genetic variants of CYP3A5, CYP2D6, SULT1A1, UGT2B15 and tamoxifen response in postmenopausal patients with breast cancer Breast Cancer Research 9:R7 doi:10.1186/bcr1640
