# Supplementary material for: Human variability in isoform-specific UDP-glucuronosyltransferases: markers of acute and chronic exposure, polymorphisms and uncertainty factors
Source: Arch Toxicol. 2020 May 15;94(8):2637–61. doi: 10.1007/s00204-020-02765-8 (PMC7395075; doi:10.1007/s00204-020-02765-8)
Supplement: Supplementary file 5 — Supplementary file5 (DOCX 4418 kb) [file 204_2020_2765_MOESM5_ESM.docx]

**Supplementary Material 5 –** *Frequency distribution figures UGT SNPs*


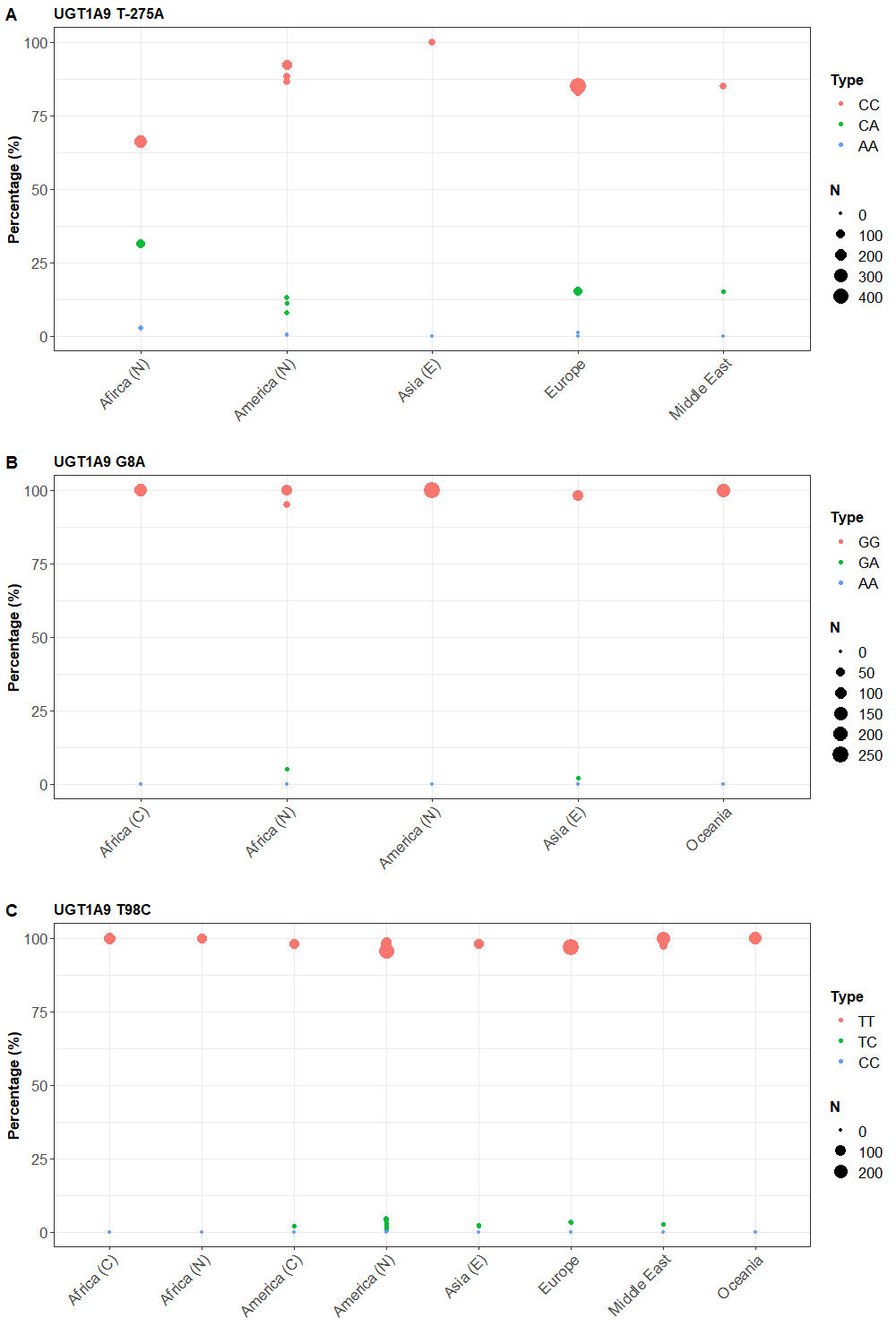


*Figure* S5.1 *Frequencies of the UGT1A9 SNPs T-275A (A), G8A (B), and T98C (C) genotypes in various ethnic groups. C= Central; N = North; E = East*
